# CTOS Board of Directors 1999–2000

**DOI:** 10.1080/13577140120048935

**Published:** 2001-03


*Sarcoma* (2001) **5**, 35–79

### CTOS Board of Directors 1999–2000

Brian O'Sullivan, MD (President)
Jaap Verweij, MD (Vice President)
Lee Helman, MD (Secretary)
Mark Gebhardt, MD (Treasurer)
Nicola Baldini, MD (past President)
Thor Alvegard, MD, PhD (Medical Oncology & Radiotherapy, 1997–2000)
Robert S. Benjamin, MD (Medical Oncology, 1997–2000)
J. Sybil Biermann, MD (1998–2001)
Vivien Bramwell-Wesley, MD (1998–2001)
Franco Gherlinzoni, MD (1999–2002)
Michael Simon, MD (1999–2002)
Ian Judson, MD (1998–2001)
Scott Nelson, MD (1999–2002)
Martine van Glabbeke MD (1999–2002)
Claude Turc-Carel, M.D (1998–2001)
Frits Van Coevorden, MD (1998–2001)
Sharon Weiss, MD (Pathology, 1997–2000)

1357–714X print/1369–1643 online/01/010035–43 © 2001 Taylor & Francis Ltd
DOI: 10.1080/13577140120048935

### 6th Annual Scientific Meeting, 2–4 November 2000, Hotel Okura, Amsterdam, The Netherlands

**Program Committee:**

Ole S. Nielsen (Chairman)
Claude S. Turc-Carel (Basic Science Program Chairman)
Mark C. Gebhardt (past Chairman)
Karen Antman (next Chairman)
Brian O'Sullivan (President)

**Scientific Committee:**

Thor A. Alvegård (Sweden), radiation oncology
Nicola Baldini (Italy), biology
Robert Benjamin (USA), medical oncology
Robert Bell (Canada), surgery
Vivien Bramwell (Canada), medical oncology
Charles Catton (Canada), radiation oncology
Frits van Coevorden (The Netherlands), surgery
George Demetri (USA), medical oncology
Christopher Fletcher (USA), pathology
Allen M. Goorin (USA), pediatric oncology
Lee Helman (USA), pediatric oncology
Peter R. Hohenberger (Germany), surgery
Ian Judson (UK), medical oncology
Jonathan J. Lewis (USA), surgery
Ole Steen Nielsen (Denmark), radiation oncology
Brian O'Sullivan (Canada), radiation oncology
Piero Picci (Italy), medical oncology
Peter Pisters (USA), surgery
Jaap Verweij (The Netherlands), medical oncology

### Program Schedule

**Thursday, 2 November 2000**

1:00–5:00 p.m. **Registration, Set Up Posters — Hotel Lobby**
6:00–7:00 p.m. **Welcome Reception — Meerman Room**

**Friday, 3 November 2000**

7:00 a.m. Registration, breakfast for meeting attendees — **Foyer Okura**
8:00 a.m. Welcome, Opening Announcements — **Heian Room**
8:15 a.m. **CASE DISCUSSION: 'Treatment of Gynecological Sarcomas'**
**Moderators & discussants:** Vivien Bramwell (London, Ont.) & Nicholas Reed (Glasgow)
9:00 a.m. **NINA AXELRAD KEYNOTE LECTURE:**
Supported by Nina Axelrad Sarcoma Fund
**Introduction/moderator:** Brian O'Sullivan (Toronto)
Murray Brennan (Memorial Sloan-Kettering Cancer Center, New York): 'Soft Tissue Sarcoma: 25 Years of Achievements, Failures and Challenges'.

9.45 a.m. **Coffee Break, Poster Viewing — Foyer Okura, Foyer Amstel & Otter, Esperance Room**

10:15 a.m. **PROFFERED PAPERS: 'Surgical Treatment of Sarcomas' — Heian Room**

**Moderators:** Antonie Tamineau (Leiden) & Frits van Coevorden (Amsterdam)

*10:15 a.m.* Chondrosarcoma of bone: analysis of factors related to prognosis in 108 cases with a minimum of two years follow-up — *Michelle Ghert*
*10:25 a.m.* The effect of re-resection in extremity soft tissue sarcoma — *Jonathan Lewis*
*10:35 a.m.* Classification of positive margins after resection of extremity soft tissue sarcoma predicts the risk of local recurrence — *Craig Gerrand*
*10:45 a.m.* Combined modality management of retroperitoneal sarcomas: phase 1 trial of pre-operative doxorubicin-based concurrrent chemoradiation surgical resections, and intraoperative electron-beam radiation therapy (IORT) — *Peter Pisters*
*10:55 a.m.* Peritoneal sarcomatosis treated by cytoreductive surgery and intraperitoneal hyperthermic perfusion — *Marcello Deraco*
*11:05 a.m.* Frits van Coevorden: 'Review of session related posters'
*11.15 a.m.* Antonie Tamineau: 'Review of session (state of the art)'

11:30 a.m. **MINI SYMPOSIUM: 'Local Recurrence of Soft Tissue Sarcomas'**
**Moderators:** Jonathan Lewis (New York) & Martin Robinson (Sheffield)

*11:30 a.m.* Surgery — *Murray Brennan*
*11:38 a.m.* Radiation Oncology — *Martin Robinson*
*11:46 a.m.* Medical Oncology — *Robert Benjamin*
*11:54 a.m.* 'General discussion'

12:15 p.m. **Lunch, Poster Viewing — Foyer Okura, Foyer Amstel & Otter, Esperance Room**

1:15 p.m. **PROFFERED PAPERS: 'Radiation Oncology' — Heian Room**
**Moderators:** Charles Catton (Toronto) & Thor Alvegaard (Lund)

*1:15 p.m.* Complete resection and intra-operative radiation therapy improve outcome of retroperitoneal sarcomas — *Jean-Pierre Pierie*
*1:25 p.m.* Radiation morbidity two years post treatment: results from a randomized trial of pre- versus post-operative radiotherapy — *Aileen Davis*

*1:35 p.m.* Real-time radiotherapy review of a randomized trial of pre-and post-operative radiotherapy for localized soft-tissue sarcoma of the extremity — *Charles Catton*
*1:45 p.m.* Thor Alvegaard: 'Review of session related posters'
*1.55 p.m.* Charles Catton: 'Review of session (state of the art)'

2:10 p.m. **PROFFERED PAPERS: 'Basic Science/Biology'**
**Moderators:** Claude S. Turc-Carel (Nice) & Jay S. Wunder (Toronto)

*2:10 p.m.* Differential expression of EZRIN in a high and low metastatic osteosarcoma model — *Chand Khanna*
*2:20 p.m.* Molecular cloning of putative oncogene and antioncogen involved in the development of osteosarcoma — *Junya Toguchida*
*2:30 p.m.* Clinico-pathological and biological analyses of the chop-related fusion genes in myxoid liposarcomas — *Taisuki Hosaka*
*2:40 p.m.* Cytokine levels in serum of the patients with soft tissue sarcomas and their relationship to alterations of routine blood tests — *Piotr Rutkowski*
*2:50 p.m.* Wild-Type P53 sensitizes soft tissue sarcoma cells to doxorubicin by downregulation of MDR1 expression — *Raphael Pollock*
*3:00 p.m.* Jay Wonder: 'Review of session related posters'
*3:10 p.m.* Claude Turc-Carel: 'Review of session (state of the art)'

3:25 p.m. **Coffee Break, Poster Viewing — Foyer Okura, Foyer Amstel & Otter, Esperance Room**

3:55 p.m. **CTOS YOUNG INVESTIGATOR AWARD PRESENTATIONS — Heian Room**
**Moderator:** Nicola Baldini (Bologna)

*3:55 p.m.* Nicola Baldini: 'Short introduction'
*4:00 p.m.* Award Presentation 1: Regulation of osteosarcoma (OS) metastasis by IGF-I receptor signaling: a novel therapeutic target — *Kristy Weber*
*4:20 p.m.* Award Presentation 2: Results of two consecutive phase II studies and interim analysis of a phase III intergroup study of neoadjuvant treatment including regional hyperthermia (RHT) in high risk soft tissue sarcoma (HR-STS) — *Clemens Wendtner*

4:40 p.m. **PROFFERED PAPERS: 'Medical Oncology'**
**Moderators:** Robert Benjamin (Houston) & Ian Judson (London)

*4:40 p.m.* Expression of the trail receptor dr4 in human soft tissue sarcomas — *Rudy Komdeur*
*4:50 p.m.* Dominant negative IkBa Potentiates in anti-tumor activity of doxorubicin in a rat hind limb isolated perfusion model — *Robert Davidson*
*5:00 p.m.* A pilot study of short course intensive multiagent chemotherapy for poor risk osteosarcoma — *Jim Janinis*
*5:10 p.m.* A phase 1 trial of intraperitoneal hyperthermic chemotherapy for the treatment of sarcomatosis — *Malcolm Bilimoria*
*5:20 p.m.* Ian Judson: 'Review of session related posters'
*5:30 p.m.* Robert Benjamin: 'Review of session (state of the art)'

5:45 p.m. **Adjourn**

6:15 p.m. **Reception and Boat Tour**

7:15 p.m. **Dinner Banquet — Ballroom I & II**

**Saturday, 4 November 2000**

7:00 a.m. Registration, breakfast for meeting attendees — **Foyer Okura**

8:00 a.m. **WORKSHOP: 'Management of osteosarcomas'**
**Moderators:** Piero Picci (Bologna) & Robert Grimer (Birmingham)

*Suggested topics:*

*8:00 a.m.* Histopathological problems — *Pancras Hogendoorn*
*8:20 a.m.* Surgical aspects. New developments — *Robert Grimer*
*8:40 a.m.* Role of high dose Methotrexate — *Kirsten Sundby Hall*
*8:55 a.m.* Treatment of high risk patients — *Piero Picci*
*9:15 a.m.* Late effects of the treatment of bone tumors — *Juliet Hale*
*9:30 a.m.* Long-term problems after surgery — *Per-Ulf Tunn*
*9:45 a.m.* 'General discussion'

10:00 a.m. **Coffee Break, Poster Viewing — Foyer Okura, Foyer Amstel & Otter, Esperance Room**

10:30 a.m. **COOPERATIVE GROUP SESSION: 'How do we improve Intergroup collaboration?' — Heian Room**
**Moderators:** Peter Pisters (Houston) & Peter R. Hohenberger (Berlin)

*10:30 a.m.* How to moderate intergroup studies — *M. van Glabbeke*
*10:45 a.m.* **Panel discussion:** 'How to improve International Collaboration and Research?'

*Panel members:*

Peter Pisters: 'ACOSOG' & 'RTOG'
Lee Helman: 'Pediatric Coop. Group'
John Edmonson: 'ECOG'
Ian Judson: 'EORTC'
Bihn N Bui: 'French SG'
Piero Picci: 'Italian SG'
Vivien Bramwell: 'NCIC-CTG/CSG'
Thor Alvegaard: 'SSG'

*11:45 a.m.* Peter Pisters & Peter R. Hohenberger: 'Summary of discussion and conclusions (how to proceed?)'

12:00 noon **Lunch, Poster Viewing — Foyer Okura, Foyer Amstel & Otter, Esperance Room**

1:00 p.m. **PROFFERED PAPERS: 'Pediatric Oncology' — Heian Room**
**Moderators:** Lee Helman (Bethesda) & Axel Le Cesne (Paris)

*1:00 p.m.* CD99 engagement: an effective therapeutic strategy for Ewing tumors — *Katia Scotlandi*
*1:10 p.m.* Induction of chemoresistence to doxorubicin in cells carrying a P53 germline mutation detected in a Li–Fraumeni family — *Luca Sangiorgi*
*1:20 p.m.* Study of age as major prognostic factor in localised Ewing's sarcoma — *Nicole Delepine*
*1:30 p.m.* Lee Helman: 'Review of session and session related posters'

1:50 p.m. **PROFFERED PAPERS: 'Diagnostic Imaging/Pathology'**
**Moderators:** Pancras Hogendoorn (Leiden) & Laurence Baker (Ann Arbor)

*1:50 p.m.* Monitoring the effect of isolated limb perfusion in soft tissue sarcoma with dynamic contrast-enhanced MR imaging — *C.S.P. van Rijswijk*
*2:00 p.m.* Upregulation of PTHrP and BCL-2 expression characterizes early malignant transformation of osteochondroma towards peripheral chondrosarcoma and is a late event in central chondrosarcoma — *JVMG Bovee*

| | |
|---|---|
| *2:10 p.m.* | Does the histologic subtype of high-grade central osteosarcoma influence the response to treatment with chemotherapy and does it affect overall survival — *E. Hauben* |
| *2:20 p.m.* | Round-cell and myxoid liposarcoma of the extremities. A clinicopathologic study of 102 cases — *Andrea Pellacani* |
| *2:30 p.m.* | Laurence Baker: 'Review of session related posters' |
| *2:40 p.m.* | P. Hoogendoorn: 'Review of session (state of the art)' |

3:05 p.m. **Coffee break — Foyer Okura, Foyer Amstel & Otter, Esperance Room**

3:35 p.m. **MINI SYMPOSIUM: 'New Drugs in Sarcomas' — Heian Room**
**Moderators:** George Demetri (Boston) & Jaap Verweij (Rotterdam)

| | |
|---|---|
| *3:35 p.m.* | Troglitazone and newer PPAR-gamma receptor ligands in liposarcomas — *George Demetri* |
| *3:50 p.m.* | ET-743 in soft tissue sarcomas— *Jaap Verweij* |
| *4:05 p.m.* | New emerging concepts — *Ian Judson* |
| *4:20 p.m.* | 'General discussion' |

4.30 p.m. **Closing. Summary of Scientific Meeting. Next Meeting.**
**Reviewer/presenter:** Karen Antman (New York)

4:45 p.m. **Member's Business Meeting**

6:00 p.m. **Adjournment**

### Proffered Papers — Surgical Treatment of Sarcomas

### Chondrosarcoma of Bone: Analysis of Factors Related to Prognosis in 108 Cases With a Minimum of Two Years Follow-up

Rizzo M, Ghert MA, Harrelson JM, Scully SP
*Duke University Medical Center, Durham, NC 27710, USA*

*Introduction:* Chondrosarcoma is unresponsive to existing adjuvant therapies and is primarily a surgical disease. There is an established relationship between the histologic grade of these tumors and prognosis. The purpose of this study was to review our institution's experience with chondrosarcoma and assess factors related to prognosis and outcome.
*Methods:* The medical records of 108 patients were retrospectively reviewed. Data was evaluated with respect to patient demographics, tumor location, histologic grade, tumor size, surgical margins, metastases and recurrence. The tumors were sub-classified based on histologic grade with grade 1 lesions defined as low-grade, and grade 2 and 3 lesions (as well as dedifferentiated lesions) defined as high-grade. All patients were followed for a minimum of 2 years. Statistical analysis was performed using univariate, multivariate, and Kaplan–Meier survival analysis.
*Results:* There were 68 males and 40 females with a mean age at presentation of 53 years (range, 26–70 years). Clinical follow-up averaged 97 months (range, 3–314). The most common tumor locations included the femur (46), pelvis (22) and humerus (10). There were 31 low-grade and 77 high-grade chondrosarcomas. One hundred and one patients underwent surgical resection. Wide margins were achieved in 78 patients, 11 underwent marginal resection and 12 tumor resections had positive margins (intralesional). Seventy-two patients remained alive at the time of this study with no evidence of disease, 23 have died of disease, six died without disease and seven remain alive with recurrent disease. The high-grade tumors had a significantly increased rate of death due to disease ($p < 0.01$), development of metastases ($p < 0.01$), and local recurrence ($p < 0.01$). There was a significant relationship between local recurrence and positive margins ($p < 0.03$), and between metastases and positive margins ($p < 0.03$). Patient demographics, tumor location and size did not correlate significantly with outcome.
*Conclusions:* This study supports previous findings that tumor grade in chondrosarcoma has prognostic significance, and that adequate surgical margins are essential to maximize survival. As chondrosarcoma does not respond to standard chemotherapy or radiation protocols, our findings emphasize the need for molecular markers and novel biologic adjuvant therapies.

### The Effect of Re-resection in Extremity Soft Tissue Sarcoma

Lewis JJ, Leung D, Espat J, Woodruff JM, Brennan MF
*Memorial Sloan-Kettering Cancer Center, New York, NY 10021, USA*

*Introduction:* This study was undertaken to determine if re-resection impacts on disease-specific survival in patients with inadequately resected, primary extremity soft tissue sarcoma. We analyzed two groups of patients: those who underwent a single definitive radical resection at a specialist cancer center versus those who underwent an incomplete excisional resection in the community, followed by a second definitive radical re-resection at a specialist cancer center.
*Methods:* Patients who underwent treatment for primary tumors (from July 1982 to June 1999) at a single institution were the subject of study. Two groups of patients were analyzed: those who underwent one definitive radical resection (one operation) and those who were previously resected and then referred for subsequent radical re-resection (two operations). Survival was determined with the Kaplan–Meier actuarial method. Statistical significance was evaluated using log-rank testing and Cox model stepwise regression.
*Results:* During this time, we resected 1092 patients with primary extremity soft tissue sarcoma. Of these, 685 underwent definitive radical resection and 407 underwent re-resection after undergoing excisional resection elsewhere. The median follow-up was 4.8 years. The 5-year disease-specific survival of the definitive resection (one operation) group was 68% and that of the re-resection (two operations) group was 84% ($p = 0.0001$). On multivariate analysis, re-resection was adjusted and controlled for age, grade, depth, size, histology and margins. Re-resection (two operations) remained a significant predictor of improved disease-specific survival ($p = 0.003$), even after these adjustments. In order to further determine whether this difference was stage or referral biased, we divided the patient population by AJCC stage. In all stages there was a trend to improved outcome, and this was most marked and statistically significant ($p = 0.005$) for those with AJCC Stage III disease (> 5 cm, high-grade and deep).
*Conclusions:* These data suggest that patients with extremity soft tissue sarcoma who undergo re-resection with two 'primary' operations have an improved survival compared with those who undergo one operation. The most plausible explanation, referral and selection bias is questionable given the significance of re-resection as a variable, even after adjusting for stage and other high-risk factors. This suggests that where possible, re-resection (two operations) should be liberally applied in patients with primary extremity soft tissue sarcoma.
(*Ann Surg*, in press). Paper will be presented by Murray F. Brennan, and we would like consideration for Jonathan J. Lewis (non-member) to present.

### Classification of Positive Margins After Resection of Extremity Soft Tissue Sarcoma Predicts The Risk of Local Recurrence

Gerrand CH, Wunder JS, Griffin A, Kandel RA, O'Sullivan B, Catton CN, Bell RS, Davis AM
*Mount Sinai Hospital, Toronto, Ont., Canada M5G 1X5, and Princess Margaret Hospital, Toronto, Ont., Canada M5G 2C1*

*Introduction:* Local failure after combined treatment of soft tissue sarcoma by surgery and radiotherapy is highly associated with a positive resection margin. We *a priori* hypothesized that patients who have a positive margin can be classified on the basis of their clinical features into groups that are at low or high risk of local recurrence. Four groups were defined.
Group 1, low grade liposarcomas (low risk). The positive margin followed an intentionally marginal excision of a low-grade liposarcoma.
Group 2, planned positive margin against a critical structure (low risk). In order to preserve a functional extremity, a positive margin was accepted against a critical structure (nerve, vessel or bone).
Group 3, prior unplanned excision (high risk). An intralesional unplanned excision was performed prior to referral. A positive margin was found after resection of the residual tumour in our centre.
Group 4, unplanned positive margin (high risk). A positive margin was unexpectedly found during primary resection of tumour, usually following surgical error.
*Methods:* To test this hypothesis, we used a prospectively collected database, containing 537 patients who underwent surgical excision of an extremity soft tissue sarcoma in our hospital and had the potential for 3 years of follow-up. There were positive margins in 112 cases: 87 of these had undergone a standard treatment regime of surgery and adjuvant radiotherapy. Twenty-five patients were excluded because they did not receive radiotherapy (13), they received chemotherapy (7), or the procedure was not intended to be curative (5). Mean follow-up was 4.0 years (0.2–9.3). Patients were assigned to groups by one investigator who reviewed the clinical records and was blinded to outcome.
*Results:* Group 1 tumours were low grade by definition. Groups 2, 3 and 4 did not differ from each other significantly by histological grade, length of follow-up, patient age, gender, or anatomical location.

| | Group 1 | Group 2 | Group 3 | Group 4 |
|---|---|---|---|---|
| Number of patients | 24 | 28 | 19 | 16 |
| Number of local recurrences | 1 (4%) | 1 (4%) | 6 (32%) | 6 (38%) |

The rate of local recurrence in group 2 was significantly less than group 3 ($p = 0.01$) and group 4 ($p = 0.01$). There was no significant difference between groups 3 and 4. Twenty-two patients died or were lost to follow-up before 2 years. When these patients were excluded from the analysis, the differences in local recurrence between the groups remained significant.
*Conclusion:* Planned positive margins against critical structures by experienced surgical oncologists represent a low risk for tumour recurrence. Classifying patients with positive margins into groups according to clinical setting provides a useful indication of the risk of local recurrence following local treatment of soft tissue sarcoma.

**Combined Modality Management Of Retroperitoneal Sarcomas: Phase I Trial Of Pre-operative Doxorubicin-based Concurrent Chemoradiation, Surgical Resection, And Intraoperative Electron-beam Radiation Therapy (Iort).**

Pisters PWT, Patel SR, Crane C, Feig BW, Hunt KK, Burgess MA, Papadopoulos NE, Plager C, Benjamin RS, Pollock RE, Janjan NE
*University of Texas M.D. Anderson Cancer Center, Houston, TX 77030, USA*

*Background:* Patterns of failure for patients with retroperitoneal sarcomas (RPS) demonstrate that the majority of patients develop local recurrence. Strategies to enhance to efficacy/intensity of local therapies are needed. One approach involves the use of pre-operative chemoradiotherapy (chemoXRT). This protocol explores the use of pre-operative doxorubicin given by protracted venous infusion (PVI) with concurrent external beam radiotherapy (EBRT). This approach takes advantage of the benefits of pre-operative radiotherapy, the activity of doxorubicin in STS, and the radiosensitizing properties of doxorubicin. When chemo XRT is combined with surgical resection and intraoperative electron-beam radiation therapy (IORT), local therapy is maximized. Objectives of this phase I trial included: (1) define the toxicities of pre-operative PVI doxorubicin and concurrent EBRT followed by surgical resection with IORT; and (2) establish the maximum tolerated dose (MTD) of EBRT when combined with PVI doxorubicin.
*Methods:* Patients with localized, resectable, grade II or III primary or recurrent RPS are eligible. Pre-operative continuous infusion doxorubicin is administered (4 mg/m$^2$ over 24 hours x 4 days/week for 4 weeks) with concomitant escalating doses of EBRT (1.8 Gy/fraction). The dose escalation scheme (and number of patients treated) for successive cohorts of patients has been: 18 Gy (three patients), 30.6 Gy (three patients), 36 Gy (three patients), 41.4 Gy (three patients), 46.8 Gy (12 patients), and 50.4 Gy (two patients).
*Results:* Twenty-six patients have been treated. The median tumor size was 12 cm (range, 6–31 cm). Histologies included leiomyosarcoma ($n = 8$), liposarcoma ($n = 5$), malignant fibrous histiocytoma ($n = 5$), unclassified soft tissue sarcoma ($n = 5$), and other RPS ($n = 3$). The MTD has not yet been defined. Only one patient experienced grade IV neutropenia at 18 Gy and there were no episodes of febrile neutropenia. Grade III gastrointestinal toxicities have included diarrhea (one patient each at 18, 30.6, and 50.4 Gy) and nausea (one patient each at 46.8 and 50.4 Gy); no patients have experienced grade IV gastrointestinal toxicities. Twenty-one patients have undergone surgical resection. IORT (15 Gy) was provided to 15 patients with no identifiable toxicities. No major wound complications have been observed.
*Conclusions:* (1) Doxorubicin-based concurrent chemoradiation can be given to a dose of 46.8 Gy with minimal grade III/IV toxicities; (2) MTD has not yet been defined; and (3) no identifiable toxicities are attributed to IORT. This combined modality approach appears to have an acceptable overall toxicity profile and capitalizes on all of the advantages of pre-operative/intraoperative treatment to enhance the therapeutic ratio of surgery and radiotherapy in the management of RP STS.

**Peritoneal Sarcomatosis Treated By Cytoreductive Surgery And Intraperitoneal Hyperthermic Perfusion**

Deraco M, Gronchi A, Pennacchioli E, Baratti D, Bertulli R, Casali PG, Rasponi A, Dileo P, Pilotti S, Vaglini M, Azzarelli A
*Istituto Nazionale Tumori, Milan, Italy*

*Intervention:* Peritoneal sarcomatosis (PS) is a very aggressive condition with a poor prognosis. We propose to investigate the effect of an aggressive surgery followed by intra peritoneal drugs delivery and local hyperthermia.
*Patients and methods:* In a phase II clinical study, 21 patients (eight men and 13 women) with PS were treated by cytoreductive surgery (CRS) and intraperitoneal hyperthermic perfusion (IPHP). The median age was 52.3 years (range, 29–74 years). The mean follow-up was 15.6 months (range, 2–44 months). Twelve patients (57%) presented retroperitoneal sarcomas and nine (43%) patients had visceral ones. Nine, three and nine patients presented grade 1, 2 and 3, respectively. Nine out of 21 (43%) and four out of 21 (19%) patients were pre-treated with systemic chemotherapy and radiotherapy, respectively. According to the Japanese classification of intraperitoneal disease extension for gastric cancer, two (10%), 11 (52%) and eight (38%) cases presented P1, P2, and P3 dissemination, respectively. Eighty percent of the patients were rendered optimally cytoreduced (cc-0/cc-1). The IPHP was carried out with the closed abdomen technique, using a preheated polysaline perfusate containing CDDP + MMC or CDDP + DX through a heart-lung pump at a mean flow of 700 ml/min for 60 minutes from the hyperthermic phase (42.5°C).
*Results:* The overall treatment toxicity and surgical morbidity rates were 14 and 15%, respectively. The treatment related mortality was 0%. Median survival and median progression free survival were 26 and 6.7 months, respectively. Median time to local progression was 16.3 months.
*Conclusions:* The results of our study are promising and a randomised controlled clinical trial should be addressed for confirmation.

**Proffered Papers — Radiation Oncology**

**Complete Resection And Intra-operative Radiation Therapy Improve Outcome Of Retroperitoneal Sarcomas**

Pierie JPEN, Betensky RA, Choudry U, Willett CG, Souba WW, Ott MJ
*Massachusetts General Hospital Cancer Center, Boston, MA 02114, USA*

*Objective:* The assessment of long-term outcomes of patients with retroperitoneal sarcomas (RS) undergoing resection and external beam radiation therapy (EBRT) with or without intra-operative electron beam radiation therapy (IOERT).

*Summary and background data:* Despite improved imaging, surgical techniques, and technical innovations in radiation therapy, the survival of patients with RS is still poor. Survival might be enhanced with improved local control, when IOERT is added to the treatment regimen.

*Methods:* One hundred and three consecutive patients treated for primary RS were studied. The median follow-up was 27 months (range, 1–193 months). Demographic features, clinical presentation, stage and histology of the tumor, the type of surgical treatment, and the addition of EBRT and IOERT were analyzed to determine their impact on survival and recurrence.

*Results:* The mean age at presentation was 55 ± 17 years (range, 10–93 years), with a slight female preponderance (56:47). Sixty-six percent of the patients presented with of pain or discomfort, 30% with a palpable mass, 23% with distant disease, and 5% with lymph node metastases. The mean tumor size was 15 ± 6 cm (range, 3–34 cm). The most common histologic type was leiomyosarcoma (27%) with predominately high-grade tumors (86%). Complete gross resection of the tumor was possible in 61% of patients and this increased survival versus both debulking (hazard ratio [HR] = 0.30, $p = 0.0005$) and biopsy (HR = 0.22, $p < 0.0001$). The 5- and 10-year survival rates were 62 and 52%, respectively, for those with complete resection versus 29 and 20% after incomplete resection.

In all 103 patients, IOERT plus EBRT enhanced survival as compared with EBRT alone (HR = 0.40, $p = 0.058$). Five- and 10-year survival rates were 70% after the use of IOERT. In a multivariate model including all 103 patients, male gender, increasing size of the tumor, a more advanced stage of the tumor, the resection of more than one organ, a histology of malignant Schwannoma, incomplete resection of the tumor and the absence of IOERT, were associated with a decreased survival.

Among the 62 patients undergoing a complete resection, there was a trend for IOERT to further augment survival as compared with EBRT alone (HR = 0.38, $p = 0.13$), leading to 5- and 10-year survival rates of 77%. IOERT increased the time to both local and distant recurrence as compared with EBRT alone (HR = 0.27, $p = 0.036$) in this group.

*Conclusions:* Complete gross resection remains the most effective treatment for retroperitoneal sarcomas. The addition of IOERT to EBRT is more effective than EBRT alone in increasing survival and decreasing both local and distant recurrence after complete tumor resection.

**Radiation Morbidity Two Years Post Treatment: Results From A Randomized Trial Of Pre- Versus Post-operative Radiotherapy**

Davis AM, O'Sullivan B, Catton CN, Chabot P, Hammond A, Benk V, Turcotte R, Bell RS, Wunder JS, Goddard K, Day A, Sadura A, Pater J, and Zee B

*Canadian Sarcoma Group and the National Cancer Institute of Canada-Clinical Trials Group, Canada*

*Purpose:* The objectives of this study were: (1) to determine if there was a difference between patients treated with pre-operative (pre-op) versus post-operative (post-op) radiotherapy in the secondary endpoints of the SR.2 trial, specifically RTOG skin and subcutaneous tissue, bone toxicity, joint stiffness and oedema; and (2) to evaluate the relationship of these endpoints to function as measured by the Musculoskeletal Tumor Rating Scale (MSTS) and the Toronto Extremity Salvage Score (TESS), at 2 years post-treatment.

*Methods:* The sample analyzed included a subgroup of 113 patients from the SR.2 study who were randomized to pre-op versus post-op radiotherapy and who had primary wound closure. The morbidity endpoints dichotomized at a score of $< 2$ or $\geq 2$ were evaluated by treatment arm using the Chi-square test. Multivariate step-wise logistic regression was used to evaluate the effect of treatment arm, radiation field size, maximal radiation dose, MSTS and TESS scores on skin and subcutaneous tissue, bone toxicity, joint stiffness and oedema.

*Results:* There was no difference in skin, bone, or joint toxicity in the two treatment arms. Twenty-six of 46 in the post-op arm compared with 19 of 67 in the pre-op arm had grade 2 or greater subcutaneous fibrosis ($p = 0.003$). Oedema grade 2 or greater was more frequent in the post-op arm (11 of 46) versus five of 67 in the pre-op arm ($p = 0.014$). In univariate analysis, $d_{max}$ dose and TESS score were associated with subcutaneous fibrosis, bone and joint toxicity. In multivariate analysis, only field area was associated with skin toxicity ($p = 0.14$); field area ($p = 0.0002$) and maximum radiation dose ($p = 0.0563$) were associated with subcutaneous fibrosis (with treatment arm confounded by field area); bone toxicity was associated with the TESS score ($p = 0.004$); maximal radiation dose ($p = 0.0052$) and MSTS ($p = 0.0001$) were associated with joint toxicity; and, field area was associated with oedema ($p = 0.0054$).

*Summary and conclusions:* Patients treated with post-op radiotherapy have greater subcutaneous tissue toxicity and oedema. However, these effects are confounded by larger radiation field area and maximum dose in the post-op treatment arm. Bone and joint toxicity have a significant impact on patient function.

**Real-time Radiotherapy Review Of A Randomized Trial Of Pre- And Post-operative Radiotherapy For Localized Soft-tissue Sarcoma Of The Extremity**

Catton CN, Goddard K, O'Sullivan B, James K

*Departments of Radiation Oncology, The Princess Margaret Hospital, Toronto, and The Cancer Control Agency of British Columbia. National Cancer Institute of Canada (NCIC) Clinical Trials Group, Kingston, Ont., Canada*

*Purpose:* To evaluate the process and results of real-time radiotherapy (RT) review of the NCIC SR-2 randomized trial of pre- and post-operative RT for localized extremity soft-tissue sarcoma (STS).

*Material and methods:* The trial opened in 1994 and closed in 1997 after the planned interim analysis showed a significant difference in outcome for the primary endpoint between the two treatment arms. Ten centers entered 189 patients. Review of RT plans was required within three fractions from the start of therapy. Copies of simulator films, isodose distributions, prescription and dose calculations, set-up photo and diagnostic images were required for the review, and were to be couriered to the review center before treatment. Plans were evaluated for compliance to dose and fractionation, and that the clinical target volume (CTV) margins met minimum requirements, and were covered by the 95% isodose line, and that dose distributions were homogeneous to ± 5%. A report of protocol compliance or non-compliance with recommendations for plan modification was faxed to the submitting center and mailed to the central trial office (CTO). The final decision for any changes was left to the treating oncologist. Records of the reviews and completed treatments were analyzed to determine protocol compliance for RT given during the trial, and to determine the effectiveness of the real-time review process.

*Results:* Five patients did not receive RT, leaving 184 for analysis. The trial review rate was 161/184 (87%), as 23 eligible cases were not reviewed. One hundred and fifty-three of 161 treatments reviewed (95%) met protocol standards, including five plans modified because of the review. Eight cases (5%) were not modified as recommended. Reasons were: unknown (five cases), treatment completed before the review (two cases), disagreement about the location of the gross tumour volume (GTV) (one case). Review was completed within the first three fractions in 90/161 cases (56%). Reasons for review not performed in real time were: sufficient data, but submitted late, 56/71 (78%); incomplete data submitted, but updated late, 6/71 (8%); reviewer late in submitting review, 9/71 (12%). Review was not performed because data was not submitted, or was lost *en route* (15/23) or incomplete data was never subsequently updated (8/23).

*Conclusions:* Extremity STS is one of the most difficult sites to plan for RT, often requiring complex, and individualized plans, and RT review was an essential quality control component for the SR-2 trial. The trial achieved 87% of treatments reviewed, with 95% of these meeting protocol requirements. The real-time review rate was only 56%. Failure was usually due to the short time available for the collection and couriering of data between simulation and treatment. For future trials, on-line data transmission should improve efficiency and reduce the risk of data being lost in transit. Thirteen percent of cases were not reviewed, and notification from CTO to reviewers and centers of upcoming reviews might lessen the rate of forgotten and lost submissions. CTO should ascertain that non-compliant plans are modified to meet protocol, or that an explanation for non-compliance is recorded.

### Proffered Papers — Young Investigator

### Regulation Of Osteosarcoma (Os) Metastasis By Igf-i Receptor Signaling: A Novel Therapeutic Target

Weber K, Tsan R, Doucet M, MacEwen E, Radinsky R
*MD Anderson Cancer Center, Houston, TX 77030, USA*

Patients with metastatic OS continue to have a survival of ~20% at 5 years despite aggressive chemotherapy and multiple thoracotomies. Increased understanding of the biology of OS progression, metastasis, and its resistance to chemotherapy will uncover new molecular targets. OS overexpresses insulin-like growth factor I receptor (IGF-I-R), and recent data suggests that OS progression and metastasis requires a functional IGF-I-R. The purpose of this study was to abolish IGF-I-R activity in highly metastatic OS cells, and to test its effects on growth and metastatic properties *in vitro* and in an orthotopic nude mouse model. Cellular proliferation assays of the human SAOS-2 and metastatic variant SAOS-LM2 OS cells revealed a 40% increase in proliferation subsequent to IGF-I treatment, compared with controls. A 30% inhibition of growth was observed following treatment with IGF-BP-3, a negative regulator of IGF-I. A chemoresistance/survival advantage was also observed for SAOS-LM2 OS cells in the presence of IGF-I as shown by reduction in doxorubicin-induced apoptosis from 62% (doxorubicin alone) to 21% (doxorubicin + IGF-I) ($p < 0.05$). Enforced expression of a dominant negative truncated IGF-I-R (952-STOP) in these cells resulted in a 50–85% decrease in IGF-I-R autophosphorylation compared with controls following IGF-I treatment. There was also a corresponding decrease in downstream AKT and MAP kinase activity. OS 952-STOP clones had a 30% longer doubling time under anchorage-dependent conditions, and failed to form colonies under anchorage-independent conditions versus controls. *In vivo* experiments in an orthotopic nude mouse model are ongoing to test the contribution of IGF-I-R to OS growth, metastasis and response to therapy. Preliminary results show that mice injected with the parental OS cells form lung metastasis, whereas those injected with the dominant negative IGF-I-R transfectant cells do not.

### Results Of Two Consecutive Phase Ii Studies And Interim Analysis Of A Phase Iii Intergroup Study Of Neoadjuvant Treatment Including Regional Hyperthermia (Rht) In High Risk Soft Tissue Sarcoma (Hr-sts) Patients

Wendtner CM, Abdel-Rahman S, Falk MH, Lang NK, Krych M, Hiddemann W, Issels RD
*Med. Klinik III/Grosshadern, Ludwig-Maximilians-University and KKG Hyperthermie, GSF, D-81377 Munich, Germany*

We report on 113 patients with HR-STS (non-resectable primary/S1, recurrent/S2, inadequately resected/S3) located within extremities, trunk or abdomen who were treated within a neoadjuvant phase II protocol (RHT-91 or RHT-95). HR-criteria were: tumor grade II/III + tumor size (> 8 cm for RHT-91; > 5 cm for RHT-95) + extracompartmental extension. The neoadjuvant RHT-91 protocol (59 patients) included four cycles of pre-operative chemotherapy (XT) plus RHT, followed by surgery and four cycles of adjuvant XT plus RHT. In addition, R1/R2-resected patients received radiation. The RHT-95 protocol (54 patients) was identical except that patients after surgery obtained XT without RHT and adequate radiation regardless of resection status. XT of both studies consisted of etoposide (125 mg/m$^2$) on day 1 + 4, ifosfamide (1500 mg/m$^2$) on day 1–4, and adriamycin (50 mg/m$^2$) on day 1 (EIA). RHT (1 h at 42.5°C) was given on day 1 + 4. Radiographic response in 52 evaluable patients of the RHT-91 (42%) and in 36 assessable patients of the RHT-95 study (33%) included 1 + 2 CR, 8 + 6 PR, 13 + 4 MR, 17 + 10 SD and 13 + 14 PD, respectively. Among 74 patients undergoing surgery, the amputation rate was < 15%. After different median observation times (RHT-91, 58 months/RHT-95, 30 months), probability of overall survival (42% versus 48%; $p = 0.392$) and distant progression free survival (51% vesrus 64%; $p = 0.357$) are quite similar for both studies. Subgroup analysis (S3 versus S1, S2) for overall survival revealed also no significant difference (47% versus 48%; $p = 0.616$). Interestingly, local relapse free survival was in favour of the RHT-91 study, which included pre- and post-operative RHT (58% vesrus 57%; $p = 0.021$).
Based on these results, a randomized prospective phase III intergroup study (EORTC 62961/ESHO RHT-95) with transatlantic participation is ongoing comparing EIA ± RHT in previously defined (S1–S3) risk groups, and includes pre- and post-operative RHT in the experimental treatment arm with local relapse free survival as the main study endpoint. Since July 1997, more than 100 patients have been randomized. Feasibility of pre-operative XT was 95% (270 of 284 cycles in 71 patients evaluable) and that of post-operative XT 74% (115 of 156 cycles in 39 patients evaluable), respectively. In all patients assessed so far, no grade IV hematological toxicity was observed. Three patients died of acute non-hematological toxicity (4%). Feasibility of pre-operative RHT was 90% (133 of 148 cycles in 37 patients evaluable) and of post-operative RHT 59% (45 of 76 cycles in 19 patients evaluable), respectively, while almost no severe reactions directly attributed to hyperthermia were reported. Taken together, treatment within this international phase III protocol is a feasible and safe approach for HR-STS patients, while impact on local disease control and survival has to be awaited.

### Proffered Papers — Basic Science/Biology

### Differential Expression Of Ezrin In A High And Low Metastatic Osteosarcoma Model

Khanna C, Prehn J, Khan J, Nguyen P, Trepel J, Meltzer P, Helman L
*Pediatric Oncology and Medicine Branches, National Cancer Institute, and Cancer Genetics Section, National Human Genome Research Institute, National Institutes of Health, Bethesda, MD 20892, USA*

Osteosarcoma (OSA) is the most common primary tumor of bone. Despite successful control of the primary tumor and adjuvant chemotherapy, relapse of OSA in the lungs occurs in over 30% of patients within 5 years. In order to understand the complex process of metastasis from appendicular tumors to the lungs, we have used cDNA microarray to define differences in gene expression between clonally related murine model variants of osteosarcoma that differ in pulmonary metastatic potential. The murine osteosarcoma models are characterized by orthotopic growth at appendicular sites in balb/c mice, spontaneous pulmonary metastasis to the lungs, and clonally related variants (K7M2 and K12) that differ in pulmonary metastatic potential.

A 4000 gene cDNA microarray was used to compare gene expression between the primary tumors of the more aggressive (K7M2) and less aggressive (K12) models. Differentially expressed genes were defined by a red to green ratio not equal to 1.0 with 99% confidence (> 1.0 or < 1.0 ± 99% confidence interval), a mean maximum signal intensity greater than 2000, and concordant differential expression in two separate microarray hybridizations. Eighty genes were defined as differentially expressed between K7M2 and K12. Forty-two genes were over-expressed in K7M2 compared with K12, and 38 genes were over-expressed in K12 compared with K7M2.

A functional approach to the analysis of the differentially expressed genes was taken, by assigning each gene to six non-mutually exclusive metastasis-associated categories (using the PubMed and OMIM databases): proliferation and apoptosis, motility and cytoskeleton, invasion, immune-surveillance, adherence and angiogenesis. The high and low metastatic variants were then evaluated within each of these metastasis-associated processes. Functional studies demonstrated significant differences in motility, adherence, and angiogenesis that favored the aggressive behavior in K7M2 compared with K12. For this reason, the differentially expressed genes with motility, adherence, and angiogenesis associated functions were considered more likely to explain differences in the metastatic behavior of K7M2 and K12 than genes associated with proliferation and apoptosis, invasion and immune-surveillance.

This approach brought attention to ezrin, a motility and adherence gene not previously described in OSA, that was found overexpressed in K7M2 compared with K12 tumors. We have confirmed differential expression of ezrin by Northern analysis. Immunocyostaining for ezrin has confirmed increased levels of ezrin protein and demonstrated the enhanced localization of ezrin at the cell membrane in K7M2 compared with K12 cells. Using Northern analysis, we have demonstrated the expression of ezrin in dogs with naturally occurring osteosarcoma and in 5/5 human osteosarcoma cell lines.

Ongoing work will attempt to define the biological role of ezrin in osteosarcoma and the relevance of ERM proteins (ezrin, radixin, and moesin) in human cases of osteosarcoma.

### Molecular Cloning Of Putative Oncogene And Antioncogene Involved In The Development Of Osteosarcoma

Toguchida J. Murakami H, Nakamata T, Nakayama T. Nakamura T

*Institute for Frontier Medical Sciences and Department of Orthopaedic Surgery, Kyoto University, Kyoto, Japan*

Mutations of the Rb and p53 genes were found in approximately 60% of osteosarcomas, and hereditary mutations of either genes predispose individuals for the risk of osteosarcomas, suggesting the major role of these tumor suppressor genes in osteosarcoma. However, it is not yet clear whether mutations of both genes will be sufficient or other genetic alterations will be necessary for the development of osteosarcoma. To address this issue, we have undertaken the *in vitro* transformation experiment. First, we have established an osteoblast-like cell line, MMC2, from the p53 (–/–) mice. MMC2 showed several phenotypes as differentiated osteoblasts such as the ability to produce calcified nodules *in vitro*. To inactivate the Rb gene in MMC2, HPV16E7 was introduced by the retrovirus vector, and one cell line was established and designated as MMC2-E7. MMC2-E7 *per se* seemed to be a non-transformed cell line, because MMC2-E7 failed to make colonies in the soft agar and no constant tumor formation was observed *in vivo*. These results suggested that genetic alterations other than the mutation of the p53 and Rb genes will be involved in the development of osteosarcoma. After a prolonged latent period, however, MMC2-E7 produced tumors *in vivo*, and a cell line (MMC2-TC) was established from tumor tissues, which showed several phenotypes as a fully transformed cell. To isolate the genes responsible for the final transformation step, the differential display method was performed and two fragments, designated DDM23 and DDM36, were isolated. The expression of DDM23 was detected only in MMC2-TC, but not in MMC2 or MMC2-E7, and therefore it was considered to be a new oncogene involved in the process of malignant transformation. The expression of the other fragment, DDM36, was lost during the progression from MMC2-E7 to MMC2-TC, suggesting that it was a candidate for the new tumor suppressor gene in osteosarcoma. Functional and mutational analyses of these putative osteosarcoma-involved genes will provide the clue to understand the molecular mechanism of osteosarcoma development.

### Clinico-pathological And Biological Analyses Of The Chop-related Fusion Genes In Myxoid Liposarcomas

Hosaka T[1,2], Kanoe H[1,2], Nakamura T[2], Toguchida J[1,2]

*[1]Institute for Frontier Medical Science and [2]Department of Orthopaedic Surgery, Kyoto University, Kyoto 606-8507, Japan*

The characteristic t(12;16)(q13;p11) or t(12;22)(q13;q12) chromosomal translocations, which lead to gene fusions that encode the TLS-CHOP or EWS-CHOP chimeric protein, respectively, are associated with human myxoid/round cell liposarcomas. However, the role of those chimeric proteins in the development of liposarcomas still remains to be solved. To address this issue, we have performed the mutation analysis using clinical specimens, and also conducted the transformation experiment using fusion genes detected in the mutation analysis.

First, we have performed a reverse transcription-polymerase chain reaction to detect the TLS-CHOP or EWS-CHOP fusion transcripts in 26 liposarcomas that were diagnosed as either myxoid or round cell type. TLS-CHOP fusion transcrips were detected in 17 cases with four different subtypes, EWS-CHOP fusion transcrips were detected in three cases with two different subtypes, and no fusion transcript was detected in six cases. There was no significant correlation between any clinical or pathological findings and the subtypes of TLS-CHOP fusion transcripts. However, two cases with a novel type of EWS-CHOP fusion transcript, which was created by the fusion between EWS exon 10 and CHOP exon 2, demonstrated enormously huge tumors at the diagnosis, and both tumors were treated effectively by chemotherapy. As *in vitro* studies, we have cloned entire cDNAs of four different TLS-CHOP transcripts and introduced them to 3T3-L1 preadipocytes by retroviral transduction. These cells had no apparent growth advantage, and no colonies were found in the soft agar. However, the adipogenic differentiation was unable to be induced *in vitro*. DOL54, one of the downstream targets of TLS-CHOP genes, was upregulated in these cells. These results suggested that the function of TLS-CHOP gene in the development of liposarcomas is other than growth progression.

### Cytokine Levels In Serum Of The Patients With Soft Tissue Sarcomas And Their Relationship To Alterations Of Routine Blood Tests

Rutkowski P[1], Kaminska J[2], Rysinska A[2], Steffen J[3], Ruka W[1]

*[1]Soft Tissue/Bone Sarcomas Department, [2]Tumor Markers Department, and [3]Department of Immunology, MSklodowska-Curie Memorial Cancer Center-Institute, Roentgena 5, Warsaw, Poland*

*Introduction:* It has been demonstrated that several cytokines may be synthesized and released by many lymphoid and epithelial malignant tumors, and that raised serum level of various cytokine may affect the prognosis. It has been also reported that cytokine levels may influence some changes in the blood tests, which was also found as negative prognostic factors in patients with malignancy. There are only a few reports about cytokine production in such a rare group of malignant solid tumors as soft-tissue sarcomas. The aim of the study was to evaluate the profile of the selected cytokines in serum of untreated patients with malignant soft-tissue tumors. Then we attempted to correlate these results with the pretreatment routine blood tests (RBC, hemoglobin level (HGB), WBC, platelets count, white blood differential count — neutrocyte count, lymphocyte count (LY), monocyte count).

*Materials and Methods:* One hundred and forty-four patients (74 males, 70 female; mean age, 50.1 ± 16.9 years) with histologically proven soft-tissue sarcomas before treatment were enrolled into the study. In the study, we evaluated serum level of 13 types of cytokines and their soluble receptors (IL-IRA, IL-2SR, IL-6, IL-8, IL-10, EL-6SR, TNFRI, TNFRII, TNFA, GCSF, MCSF, bFGF, VEGF) with an ELISA method. Peripheral blood samples from 45 healthy volunteers were collected as controls. Statistical analysis was performed using Kolmogorov–Smirnov and Mann–Whitney U tests ($p < 0.05$).

*Results:* Significant differences in 11/13 cytokine productions (IL-IRA, 11-2SR, IL-6, IL-8, IL-10, TNFRI, TNFRII, TNFA, MCSF, bFGF, VEGF) ($p < 0.001$) were observed between malignant soft tissue tumors and the healthy subjects group. Elevated serum level of a complex of several cytokines (particularly, IL-IRA, IL-2SR, IL-6, IL-8, EL-10, MCSF, TNFRI) correlated significantly ($p < 0.001$) with the most frequently founded alterations in the blood tests: neutrophilia (29.5% of cases), decreased HGB (22.3%), monocytosis (22.3%) and thrombocytosis (14.5%). In 10% of patients, we found lymphocytopenia (LY < 1.0), which demonstrated the relationship to serum levels of IL-6, IL2SR, MCSF. Additionally, it was detected that increased sera several cytokin levels and also blood test abnormalities correlated significantly with more advanced stage of the primary tumor (size and grade).

*Conclusions:* The results of this study suggest that cytokine production is probably involved in soft tissue sarcoma progression, what is implied by their increased sera levels in soft-tissue sarcomas versus controls. The results of the analysis may also suggest that important biological effects and immunological implications of the particular cytokines can be reflected in routine blood test abnormalities. Sera levels of selected cytokines may be useful matker in the diagnosis of soft tissue sarcomas.

**Wild-type P53 Sensitizes Soft Tissue Sarcoma Cells To Doxorubicin By Downregulation Of Mdr1 Expression**

Zhan M, Yu D, Lang A, Li L, Pollock RE
*MD Anderson Cancer Center, Houston, Texas, USA*

*p53* mutations occur in almost one-half of all soft issue sarcomas (STS) and may be a contributing factor in their chemoresistance to most of chemotherapeutic agents including Doxorubicin (Dox), the most active single agent in this disease. To examine whether introduction of wild-type (wt) *p53* might increase chemosensitivity of STS cells harboring *p53* mutations, two wt *p53* stable transfectants of SKLMS-1 sarcoma cells, SKp53-1 and SKp53-3, and a *p53* temperature-sensitive mutant transfectant of SKLMS-1 cells, SKAla-14, were used and compared with parental cells SKLMS-1 for their sensitivity to Dox. MTT assays showed that the IC50 of Dox decreased from 2.5 mm for SKLMS-1 to 0.25 mm for SKp53-1 and 0.18 mm for SKp53-3 cells. Clonogenetic assays showed that the IC50 decreased from 27.5 mg/ml for SKLMS-1 to 2.4 mg/ml for SKp53-1 and SKp53-3 cells. In tumorigenic assays, cells were injected subcutaneously (s.c.) into SCID mice. When the resulting tumor volume reached 62.5 $mm^3$, mice were given Dox (1 mg/kg) s.c. weekly. Consequently, Dox treatment inhibited tumor growth more effectively in mice bearing SKp53-1 and SKp53-3 tumors than in mice bearing SKLMS-1 tumors. Western blot, northern blot, and immunohistochemical analyses showed that the mdr1 gene encoded p-glycoprotein expression decreased in wt *p53* transfectants compared with SKLMS-1 cells. Higher levels of intracellular accumulations of Dox were found in wt *p53* transfectants than that in SKLMS-1 cells. No difference in DNA fragmentation, Bax or Bcl-2 expression was detected. Taken together, these results suggest that introduction of wt *p53* into STS cells harboring *p53* mutation can enhance their chemosensitivity to Dox by inhibiting mdr1 expression. These results also suggest that combination of *p53* gene therapy and chemotherapy might increase the therapeutic efficacy in the treatment of STS.

**Proffered Papers — Medical Oncology**

**Expression Of The Trail Receptor Dr4 In Human Soft Tissue Sarcomas**

Komdeur R, de Jong S, Hoekstra HJ, Molenaar WM, Plaat BEC, Van den Berg E, Van der Graaf WTA
*University Hospital Groningen, 9700 RB, The Netherlands*

*Introduction:* Nowadays, limb salvage is feasible in more than 75% of patients with a primarily irresectable soft tissue sarcoma (STS) of the extremity after treatment with hyperthermic isolated limb perfusion with TNFα and melphalan (HILP-TM). The TNF related apoptosis inducing ligand (TRAIL) is a recently described member of the TNF family of cytokines that rapidly induces apoptosis in tumor cells. TRAIL appears to be non-toxic to normal tissues when administered in non-human primates. Six distinct receptors for TRAIL have been identified, of which only DR4 and DR5 can initiate cell-death. Adding TRAIL to the treatment schedule might further improve limb salvage rate for locally advanced extremity STS. HILP offers the unique possibility to introduce TRAIL in humans since unforeseen toxicity would be limited. The objectives of the current study are: (1) to investigate the expression of DR4 in human STS, and (2) to determine whether the expression of DR4 is changed after HILP-TM.

*Patients and methods:* Twenty-five patients with a primarily irresectable extremity STS underwent HILP with TNFα (3–4 mg) and melphalan (10–13 mg/l limb volume), followed by a delayed resection. The median period between HILP and resection of the tumor remnant was 61 days (range, 12–81 days). Tumor samples were obtained from the diagnostic pre-HILP specimen and from the post-HILP resection specimen. Immunohistochemical detection of DR4 was scored as described in Table 1.

*Results:* Of the 25 samples obtained before HILP-TM, 16 scored positive for DR4 expression (64%) (Fig. 1). After HILP, 18 samples were evaluable: 12 scored positive (76%) (Figure 2). Statistical analysis of paired (pre- and post-HILP) samples revealed no significant change in DR4 expression. Interestingly, all synovial sarcomas ($n = 4$) were scored negative before HILP-TM, whereas three were positively stained afterwards.

*Conclusions:* The majority of these human STS expresses the DR4 receptor. Of interest, all four synovial sarcomas scored DR4-negative before HILP; three of them were positive afterwards. DR4 expression did not significantly change in paired samples after HILP. However, acute effects may be missed due to the long period between HILP and tumor resection. The high percentage of initial DR4 positive tumors make TRAIL an interesting agent for STS when it becomes available for clinical studies.

**Dominant Negative IkBa Potentiates In Anti-tumor Activity Of Doxorubicin In A Rat Hind Limb Isolated Perfusion Model**

Davidson R, Lu X, Chiao P, Pollock R, Feig B
*University of Texas M.D. Anderson Cancer Center, Houston, TX 77030, USA*

*Introduction:* Inhibition of NF-kB activity has been shown to potentiate apoptotic killing secondary to chemotherapeutic and biologic agents. We hypothesize that direct gene transfer of the dominant negative inhibitor (IkBaM) would potentiate the *in vivo* tumor response to doxorubicin delivered via isolated perfusion in a rat hind limb fibrosarcoma model.

*Methods:* Viable tumors were established in the hind limb of Fisher rates weighing 150–250 g using a methylcholanthrene induced rat fibrosarcoma (RFS). When tumors reached 5–10 mm in greatest dimension, direct intra-tumoral injection with 6 x 1010 pfu of either Ad5IkBaM, empty Ad5 vector (EV) or a similar volume of phosphate buffered saline (PBS) was performed serially for 3 days. Rats were then perfused with doxorubicin 1.0 mg/g body weight/cm$^3$ for 10 minutes at a perfusion rate of 2.4 cm$^3$/min. Animals were subsequently observed for 21 days with serial recording of tumor volumes.

*Results:* There was a reduction in tumor volume from baseline to day 21 in the Ad5IkBaM treated group (–21%) as opposed to continued tumor growth in the EV (+58%) and PBS (+457%) treated groups. Overall, ANOVA was significant for the three groups ($p < 0.001$). The graph below shows the percent volume change for the three groups.

*Conclusions:* The addition of Ad5IkBaM potentiates the effect of doxorubicin in this isolated lower extremity perfusion model in the rat. We believe that inhibition of NF-kB activity leads to increased apoptotic sarcoma cell killing in concert with doxorubicin or other effectors that lead to apoptosis. IkBaM may represent an important adjunct to therapies based on the induction of apoptosis in the future.

### A Pilot Study Of Short Course, Intensive, Multiagent Chemotherapy For Poor Risk Osteosarcoma

Janinis J, McTiernan A, Cassoni AM, Whelan JS
*The London Bone and Soft Tissue Tumour Service, Meyerstein Institute of Oncology, Middlesex Hospital, London W1N 8AA, UK*

*Aim:* To assess the feasibility, toxicity and response to short course, multiagent chemotherapy culminating in peripheral stem cell supported high-dose chemotherapy (HDC) in patients with poor risk osteosarcoma (OS).

*Patients and methods:* Between April 1995 and April 1999, 30 patients entered the study. Median age, 24 years (range, 9–46 years). Median age for extremity OS, 17 years; for pelvic/axial OS, 30 years. Male to female ratio, 1.7:1. Primary site: extremity OS, 14; pelvic OS, 12; other, 4. Metastases at presentation, 15/30. Chemotherapy consisted of five blocks given consecutively. Block 1: 100 mg/m$^2$ cisplatin, 75 mg/m$^2$ q 14 days x 2 doxorubicin. Block 2 (x 1): 50 mg/m$^2$ cisplatin, 4 g/m$^2$ ifosfamide, and 500 mg/m$^2$ etoposide (stem cell harvest post Block 2). Block 3: 18 g/m$^2$ q 21 days x 2 ifosfamide. Block 4: 12 g/m$^2$ q 7–10 days x 3 methotrexate. Block 5 (administered around week 21): AUC8 carboplatin and 400 mg/m$^2$ etoposide on days –8, –6, and –4, 60 mg/kg cyclophosphamide on days –6 and –4. On day 0, patients underwent reinfusion of their harvested stem cells.

*Results:* A total of 226 cycles of chemotherapy (blocks 1–5) were administered. A significantly higher number of patients with extremity OS received more than 75% of the intended dose of chemotherapy or the intended number of cycles for blocks 2, 3, and 4 compared with those with pelvic/axial OS. HDC was administered to 11 patients (10 with extremity OS and one with a pelvic OS). Grade 3 or 4 toxicity (blocks 1–4): neutropenia, 49% of cycles; thrombocytopenia, 26%. There were 59 episodes of febrile neutropenia. There were two treatment-related deaths: one post-HDC from sepsis, and one during surgery. Responses (30 patients evaluable): PR, 30%; mR, 17%; SD, 47%; PD, 6%. Histologic response (22/26 evaluable patients) > 90% tumor necrosis, 23%. Twenty-seven patients underwent primary surgery. Limb salvage operation, 20. Eight patients underwent pulmonary metastasectomy. The median survival time for the whole group was 16 months (95% CI, 13–19 months). The 2-year survival rate for the whole group of patients was 33% (50% for extremity tumors and 19% for pelvic/axial tumors); median follow-up, 16 months (range, 7–57 months).

*Conclusion:* Dose intensive multiagent chemotherapy is feasible in the group of patients with extremity OS but not those with pelvic/axial primaries. A number of factors may account for this such as higher age group and poor performance status. Inferior survival rates in the pelvic/axial group are attributed to poor local tumor control by surgery and less dose intensive treatment.

### A Phase I Trial Of Intraperitoneal Hyperthermic Chemotherapy For The Treatment Of Sarcomatosis

*Bilimoria MM, Feig B, Mansfield P, Pisters PWT, Pollock RE, Patel S, Plager C, Benjamin R, Burgess M, Chase J, Murphy A, Griffin J, Mirza, N, and Hunt K*

*UT MD Anderson Cancer Center, Houston, TX 77030-4095, USA*

The appropriate therapeutic interventions for patients with intra-abdominal disseminated sarcoma (sarcomatosis) remain unclear. We have previously reported that these patients have a median survival of 13 months irrespective of the current adjuvant therapy available (CTOS abstract #0002, 1999). A phase I study using tumor debulking coupled with hyperthermic peritoneal perfusion with cisplatin was initiated to determine the toxicity, operative complications, and effects on time to tumor progression.

*Methods:* A total of 25 patients were enrolled in the study, of which 19 underwent complete tumor debulking followed by intraperitoneal hyperthermic perfusion with cisplatin. Patients with low volume liver metastases were eligible for perfusion. The dose of cisplatin used was modified from an initial 150 to 90 mg/m$^2$. Perfusate time was modified from 120 to 90 minutes. Seventeen patients received a 90 mg/m$^2$ dose with a perfusion time of 90 minutes. Inlet temperature of perfusate was decreased from 44 to 41°C. All changes were secondary to toxicity.

*Results:* Two patients were treated with the initial parameters (150 or 120 mg/m$^2$ cisplatin; 44°C inlet temperature; 120 minute dwell time). A total of 17 patients were treated with the modified dose of cisplatin (90 mg/m$^2$), the modified perfusate time (90 minutes), and the modified inlet temperature (41°C). The median age of the patients studied was 52 years (range, 24–77 years). The median number of separate tumor nodules removed was 100 (range, 6–1000+). Median time on mechanical intubation was 1 day (range, 1–39 days) with a median hospital stay of 15 days (range, 9–69 days). Median platelet nadir was 85 K (range, 9–176 K) necessitating a median of 0 (range, 0–83) platelet transfusions in these patients. The median hemoglobin nadir was 8.2 g/dl (range, 7.1–13.4 g/dl), although the median number of perioperative RBC transfusions was 5 U (range, 0–34 U). Four patients experienced major complications (24%) in this group. One patient experienced adult respiratory distress syndrome associated with sepsis, another patient experienced pulmonary edema requiring prolonged intubation, and two others experienced renal failure requiring temporary hemodialysis (both patients received an initial dose of cisplatin of 150 or 120 mg/m$^2$). There was one reoperation for post-operative bleeding and there were no perioperative deaths. Three patients died as a result of metastases to the liver 7, 11, and 16 months after the procedure. Four patients are alive with no evidence of recurrence 4–8 months following the procedure. The remaining 12 patients are alive with recurrent disease (five with recurrent peritoneal disease and seven with primary liver metastases). The median time to local recurrence was 5 months (range, 2–9 months), while the median time to distant recurrence was 4 months (range, 1.5–12 months).

*Conclusions:* This pilot study of tumor debulking and intraperitoneal hyperthermic perfusion with cisplatin in patients with sarcomatosis reveals that the procedure can be performed without mortality and with significant morbidity in only one-quarter of the patients. Although the median time to local recurrence was 5 months, 24% of the patients remain disease-free at a median of 6 months follow-up. All patients who died following the procedure died of metastases to the liver suggesting that, although the procedure can control peritoneal disease, patients are still at risk for failure from liver disease. A phase II investigation of this aggressive therapy is needed better define response rates and progression-free in a larger cohort of patients.

### Proffered Papers — Pediatric Oncology

### CD99 Engagement: An Effective Therapeutic Strategy For Ewing Tumors

Katia Scotlandi[1], Nicola Baldini[1], Vanessa Cerisano[1], Maria Cristina Manara[1], Stefania Benini[1], Massimo Serra[1], Pier-Luigi Lollini[2], Patrizia Nanni[2], Giordano Nicoletti[3], Ghislaine Bernard[4], Alain Bernard[4], Piero Picci[1]
*[1]Laboratorio di Ricerca Oncologica, Istituti Ortopedici Rizzoli, Bologna, Italy, [2]Istituto di Cancerologia, Universitá di Bologna, Italy, [3]IST, Istituto Nazionale per la Ricerca sul Cancro-Genova, Unitá Satellite di Biotecnologie, Bologna, Italy, and [4]Unitø INSERM 343, Hôspital de l'Archet, Nice Cedex 3, France*

CD99, a cell surface protein encoded by the MIC2 gene, is broadly distributed on many cell types, with a particularly strong expression on immunopoietic cells and on Ewing's sarcoma cells. Within the hemopoietic system, CD99 appears to have a role in the differentiation process of T lymphocytes, by mediating adhesion properties and apoptosis of immature thymocytes and proliferation of mature T cells. In Ewing's sarcoma, CD99 has long been considered only as an important marker for the diagnosis of these lesions. In this paper, we demonstrate that engagement of CD99 significantly inhibits the *in vitro* and *in vivo* growth ability of Ewing's sarcoma cells. In particular, ligation of CD99 with specific MAbs resulted in a significant induction of apoptosis and of the homotypic adhesion potential of these cells as well as in a significant inhibition of the ability of Ewing's cells to grow in semisolid medium and in athymic mice. The growth inhibition was significantly related with the level of expression of CD99 on the surface of the cells and was time and dose dependent. Analysis of apoptosis and of the proliferation rate revealed that CD99 engagement significantly induced apoptosis of Ewing's sarcoma cells but lacked to affect their proliferation ability. Moreover, we show that anti-CD99 MAbs may be advantageously used in association with conventional anticancer agents. These results provide a novel entry site for therapeutic intervention, which may have application in the care of patients with Ewing tumor, and warrant further studies to clarify the molecular mechanisms activated by CD99 engagement.

### Induction Of Chemoresistence To Doxorubicin In Cells Carrying A P53 Germline Mutation Detected In A Li-fraumeni Family

Sangiorgi L[1], Cerone MA[2], Soddu S[2], Gobbi G[1], Lucarelli E[1], Brach del Prever A[3], Picci P[1], Helman LJ[4]
*[1]Rizzoli Orthopedic Institute, Bologna, [2]Regina Elena Cancer Institute, Rome, [3]University of Turin, Turin, Italy, and [4]NCI, NIH, Bethesda, MD, USA*

A phenotypic Li–Fraumeni family (mother died of breast carcinoma at the age of 35, brother died of rabdomyosarcoma at the age of 3, two sisters died of osteosarcoma at the ages of 10 and 14) was investigated for the presence of germline p53 mutations. SSCP for exons 4–11 of the gene on tumor DNA from the two sisters revealed an abnormal conformer in exon 6. DNA sequence analysis showed a transversion from adenine to cytosine at codon 220 (amino acid change from tyrosine to serine). The same pattern was observed on microdissected paraffin-embedded tissue of both the mother and the brother. We generated, by site directed mutagenesis, a plasmid encoding the p53SER220 mutation (pLp53-S220). We transfected fibroblasts from p53 –/– mice (F10) with pLp-S220 and pLp53-H175 (a plasmid encoding the p53His175 mutant). The presence of the two mutations did not increase the proliferation rate of F10 fibroblast. Drug sensitivity (assessed by $IC_{50}$ value) of the fibroblasts carrying the mutations was evaluated for doxorubicin, cisplatin and 5-florouracil. Fibroblasts carrying the p53SER220 and p53His175 mutations showed a selective resistance for doxorubicin with, respectively, a 3.5- and a 2.9-fold increase. These data were confirmed by the evaluation of the clonogenic ability of the fibroblasts carrying the different transfectants. In conclusion, the p53SER220 germ-line mutation seems to induce a gain of function in term of chemoresistency for the mutated p53 protein.

### Study Of Age As Major Pronostic Factor In Localised Ewing's Sarcoma

Delepine F, Delepine N, Guikov E, Delepine G
*Oncologic Paediatric Unit, Avicenne University Hospital, 125 Bd de Stalingrad, 93009 Bobigny Cedex, France*

*Introduction:* In Ewing's sarcoma, the prognostic value of age is debated. Most early monocentric studies published disease free survival rate between 10 and 30% for adult patients compared with 20–60% for children. But other multicentric trials (IESS, CESS or SFOP) did not find such a difference. We imagined that the observed differences could be correlated with the given drug intensities, and analysed our data to prove it.
*Material:* From January 1986 to January 1999, 48 patients with localised Ewing's sarcoma of bone have been treated by our team. There were 29 males and 19 females with a median age of 18 years (range, 5–35 years). Chemotherapy started with a short bi-drug induction (6 weeks of cyclophosphamide–doxorubicin) surgery in all cases (*en bloc* resection when feasible, curettage for vertebral and sacral locations). Post-operative chemotherapy used five or six drugs (Vincristine–Dactinomycine–Ifosfamide–Cyclophosphamide–Doxorubicin or Etoposide–Cisplatinium) for 10 months.
All patients have been followed-up with physical examination, plain RX-rays, bone scan, computed tomographies of the lungs and primary site, every 3 months for 2 years, then every 6 months for 2 years, and yearly then after.
*Results:* With a median follow up of 7 years and 6 months, 37 (77 %) patients are event-free survivors.
In this series, the site of the tumor and the tumoral volume had no impact on disease free survival but only age, body surface area and response to pre-operative chemotherapy.
The life expectancy of patients under 19 years is 96% (24/25), but only 56% (13/23) for patients aged 19 or older ($p < 0.001$). The disease-free survival of patients with body surface area under 1.3 $m^2$ is 100% compared with 55% for patients with larger surface ($p < 0.001$).
The univariate analysis shows that received drug intensities of vincristine and dactinomycin are the only independent therapeutic prognostic factors (both correlated with age and body surface area). With the total dose limit of 2 mg vincristine and 2 mg dactinomycin, patients with larger surface area (> 1.3 $m^2$) received less drugs per square meter than younger patients.
In multivariate analysis, age had no prognostic value, but only the received drug intensities of Vincristine and Dactinomycin.
*Conclusion:* In Ewing's sarcoma, ageis not an independent prognostic factor, but only underlines the importance of given dose intensities of vincristine and dactinomycin.

### Proffered Papers — Diagnostic Imaging/pathology

### Monitoring The Effect Of Isolated Limb Perfusion In Soft Tissue Sarcoma With Dynamic Contrast-enhanced Mr Imaging van Rijswijk CSP[1], Smid-Geirnaerdt MJA[2], van Coevorden F[3], Kroon BBR[3], Peterse JL[4], Tollenaar RAEM[5], Taminiau AHM[6],Hogendoorn PCW[7], Bloem JL[1]
*[1]Department of Radiology, [5]Department of Surgery, [6]Department of Orthopedic Surgery, and [7]Department of Pathology, Leiden University Medical Center, Leiden (2300 RC), The Netherlands, and [2]Department of Radiology, [3]Department of Surgery, and [4]Department of Pathology, Netherlands Cancer Institute, Antoni van Leeuwenhoek Hospital, Amsterdam (1066 CX), The Netherlands*

*Purpose:* To assess whether magnetic resonance (MR) imaging, with the emphasis on dynamic contrast-enhanced MR, can determine tumor response after isolated limb perfusion with recombinant tumor necrosis factor-alpha in order to plan the moment of resection.
*Material and methods:* We prospectively included a pilot of eight patients, with proven high-grade soft tissue sarcoma, who were treated with isolated limb perfusion prior to resection. T1- and T2-weighted, static and dynamic contrast-enhanced MR images were acquired prior to and following isolated limb perfusion (immediately before surgery).
We evaluated tumor volume, signal intensity, and start and progression of tumor enhancement. Early and rapidly progressive enhancing areas versus late or non-enhancing areas seen on dynamic contrast-enhanced images were correlated with the histopathologic findings of the resected specimens, except for one patient in which tumor resection was postponed because of metastatic disease.
Pathologic response was defined as complete response (CR) if 100% tumor necrosis was present, partial remission (PR) if 50% necrosis was present, and no change (NC) if < 50% tumor necrosis was present.
*Results:* None of the patients showed pathologic CR, four of eight patients showed pathologic PR, and three of eight patients showed pathologic NC. In one patient, only clinical examination was available, which showed NC.
Tumor volume response did not correlate with pathologic response.
On dynamic contrast-enhanced images, early and rapidly progressive enhancing areas corresponded to residual viable tumor. Late and gradual enhancing areas or non-enhancing areas corresponded to (therapy-induced) necrosis, degeneration or fibrosis.
*Discussion:* Dynamic contrast-enhanced MR imaging seems accurate in classifying response to isolated limb perfusion in soft tissue sarcoma. Its potential role in planning the moment of resection of high-grade soft tissue sarcoma of the extremities merits further evaluation.

### Upregulation Of Pthrp And Bcl-2 Expression Characterizes Early Malignant Transformation Of Osteochondroma Towards Peripheral Chondrosarcoma And Is A Late Event In Central Chondrosarcoma

Bovee JVMG, Van den Broek LJCM, Cleton-Jansen AM, Hogendoorn PCW
*Department of Pathology, Leiden University Medical Center, Leiden, The Netherlands*

Chondrosarcomas are malignant cartilage-forming tumors arising centrally in bone (central chondrosarcoma), or within the cartilaginous cap of osteochondroma (peripheral chondrosarcoma). For hereditary multiple osteochondromas, two responsible genes, *EXT1* and *EXT2*, have been cloned. Their recently elucidated role in heparan sulfate biosynthesis and Hedgehog (Hh) diffusion leads to the hypothesis that *EXT* inactivation affects fibroblast growth factor (FGF) and Indian Hedgehog (IHh)/PTHrP signaling, two important pathways in chondrocyte proliferation and differentiation. We investigated the immunohistochemical expression of molecules involved in IHh/PTHrP (PTHrP, PTHrP-receptor, Bcl-2) and FGF (FGF2, FGFR1, FGFR3 and p21) signaling in osteochondromas ($n$ = 24), and peripheral ($n$ = 29) and central ($n$ = 20) chondrosarcomas. IHh/PTHrP and FGF signaling molecules are mostly absent in osteochondromas. Although no somatic *EXT* mutations were found in sporadic osteochondromas, the putative EXT downstream targets are affected similarly in sporadic and hereditary tumors. In chondrosarcomas, re-expression of FGF2, FGFR1, PTHrP, Bcl-2 and p21 is found. Expression levels increase with increasing histological grade. Upregulation of PTHrP and Bcl-2 characterizes malignant transformation of osteochondroma since PTHrP and Bcl-2 expression is significantly higher in borderline and grade I peripheral chondrosarcomas as compared with osteochondromas. In contrast, upregulation of PTHrP and Bcl-2 seems to be a late event in central cartilaginous tumorigenesis since expression is mainly restricted to high grade central tumors.

### Does The Histologic Subtype Of High-grade Central Osteosarcoma Influence The Response To Treatment With Chemotherapy And Does It Affect Overall Survival? A Study Based On The Material Of Two Consecutive Trials Of The European Osteosarcoma Intergroup (Eoi) Consisting Of 570 Patients

Hauben E, Weeden S, Pringle J, Van Marck E, Hogendoorn PCW
*University of Antwerp, Antwerp, B-2560 Belgium, and Leiden University Medical Centrum, 2300 RC Leiden, The Netherlands*

Large randomised trials are mandatory when one wants to examine the effect of different aspects (clinical, treatment modality or other) of pathological condition on overall outcome. This is especially true when studying a disease in which there is a multi-factorial influence on progression and outcome.
The data on 570 patients with biopsy proven primary central osteosarcoma of an extremity, included in two consecutive studies of the European Osteosarcoma Intergroup, were analysed to evaluate: if the histological subtype of the biopsy specimen correlates with the subtype of the resected specimen, if there is a relation between histological subtype and overall survival, and if there is a relation between histological subtype and histological response to chemotherapy.
High-grade osteosarcoma, as defined by established criteria, was subtyped as either conventional, chondroblastic, teleangiectatic, small cell, fibroblastic, osteoclast rich, anaplastic and sclerotic/osteoblastic well differentiated.
A panel of experienced pathologists was appointed to review the histological diagnosis and to assess the tumour response to chemotherapy on the resected specimen of each patient entered into the trials.
Subtyping on the biopsy specimen proved to be highly representative for the subtype of the whole tumour. In 102 patients for which subtyping was performed on the biopsy and the resected specimen, there were only two discrepancies. Of the 568 patients for whom subtype is available, 404 (71%) were of the common type, 54 (10%) were chondroblastic, 53 (9%) had fibroblastic tumours, and the remaining consisted of rare subtypes.
Good response to pre-operative chemotherapy was defined as greater than 90% necrosis. The proportion of patients responding well to chemotherapy differed significantly between subtypes (chi-square test statistics = 11.44, $p$ = 0.01 on 3 degrees of freedom (df)). In comparison with the common subtype, there was a higher proportion of good responders in the fibroblastic group and a lower in the chondroblastic. Good responders had significantly better survival than patients who responded poorly to pre-operative chemotherapy (log-rank statistic = 25.20, $p < 0.01$ on 1 df). Survival did not differ significantly according to subtype (log-rank statistic = 2.72, $p = 0.44$ on 3 df), although there is a clear suggestion that patients with chondroblastic tumours experience better long-term survival.
This large set of prospectively collected data provides important information on the relationship between pathological subtype, histological response and survival. Histological response has a known prognostic effect on survival, and we have shown that rates of response differ by subtype. There is limited evidence that some histological subtypes experience better survival. Despite this large multi-institutional study, we have insufficient numbers of non-common tumours to examine this unambiguously.

**Round-cell And Myxoid Liposarcoma Of The Extremities. A Clinicopathologic Study Of 102 Cases**

Pellacani A, Pignatti G, Bertoni F, Giunti A, Baldini N
*Istituti Ortopedici Rizzoli, 40136 Bologna, Italy*

We evaluated the clinical and pathologic features of 102 patients with biopsy proven myxoid or round cell liposarcoma examined at a single institution between 1977 and 1999. Routine hematoxylin and eosin stained slides of all cases were reviewed. Morphologic variables evaluated included percent of round cell differentiation, percent of lipoblast differentiation, and presence of tumor necrosis. Clinical follow-up was available for all patients. Flow cytometry for determination of DNA ploidy was performed on fresh tissue available from 28 cases. Survival was determined by the Kaplan–Meier test using the approximate chi-square statistic for the log-rank test.
Age at diagnosis ranged from 12 to 78 years (median, 46 years), with a significant difference between males (median, 35 years) and females (median, 66 years). The single most common location was the thigh (70 cases). Histologically, round cell differentiation was present in 35 cases. Spontaneous tumor necrosis was noted in 11 cases. By flow cytometry, 27 tumors were diploid and one was aneuploid. Twenty-four patients developed metastases at a mean of 22 months after diagnosis.
With statistical analysis, age (> 45 years), male sex ($p < 0.05$), percent of round cell differentiation (> 25%; $p < 0.05$), and the presence of spontaneous tumor necrosis were associated with a poor prognosis. No correlation was observed between DNA ploidy and percent of round cell differentiation or clinical outcome.

### Posters — Surgical Treatment Of Sarcomas

**025 The Predictive Value Of Tissue Tranglutaminase In Malignant Fibrous Histiocytoma**

Heiner JP, Hughes EM, Aeschlimann D, Hafez R, Kehoe R
*University of Wisconsin, Madison, WI 53792, USA*

**041 Pathologic Fracture In Osteosarcoma: Prognostic Significance And Treatment Implications**

Ghert MA, Zurakowski D, Gebhardt MC, Thompson RC, Scully SP
*Duke University Medical Center, Durham, NC 27710, USA*

**044 Thallium 201 Uptake In Chondroid Lesions**

Templeton KT, Raveill T, Tawfik O
*University of Kansas Medical Center, Kansas City, KS 66160, USA*

**045 Complications And Morbidity Of Adjuvant Therapy For Soft Tissue Sarcoma**

Templeton KT
*University of Kansas Medical Center, Kansas City, KS, USA*

**046 Osteochondroma Of The Femoral Neck**

Siebenrock KA, Ganz R
*Orthopaedic Surgery, University of Berne, 3010 Berne, Switzerland*

**047 Surgical Margins Influence Local Recurrence Of Soft Tissue Sarcomas In The Thigh, But Not Survival In 152 Patients**

Vraa S, Keller J, Nielson O, Jurik AG, Jenson OM
*Center for Bone and Soft-Tissue Sarcomas, University Hospital of Aarhus, DK-8000 Arhus C, Denmark*

**048 Soft Tissue Sarcomas After 80 Years Of Age. Study Of A Series Of 22 Patients**

Turcotte R, Barabas D, Isler M, Doyon J, Normandin D
*Division of Orthopaedic Surgery, Maisonneuve-Rosemont Hospital, Montreal, Que., Canada H1T 4B3*

**049 Primary Leiomyosarcoma Of Bone. A Clinicopathologic Study Of 10 Patients**

Barabas D, Turcotte R, Isler M, Doyon J, Normandin
*Division of Orthopaedic Surgery, Maisonneuve-Rosemont Hospital, Montreal, Que., Canada H1T 4B3*

**050 Giant Cell Tumor Of The Pelvis And Sacrum: Review Of Seventeen Cases And Meta-analysis**

Leggon RE, Berry BH, Zlotecki R, Reith J, Wood D, Vlak R, Scarborough MT
*University of Florida, Gainesville, FL 32610-0246, USA*

**051 Recent Experience With The Compress® Device For Compliant Fixation Of Oncologic Endoprostheses**

O'Donnell RJ[1,2], Martin DL[2], Silverstein RM[2], Johnston JO[1]
*[1]Department of Orthopaedic Surgery, Orthopaedic Oncology Service, University of California, San Francisco, 1701 Divisadero St., Suite 280, San Francisco, CA 94115-1652, USA, and [2]Department of Musculoskeletal Oncology, The Permanente Medical Group, Inc., 1200 El Camino Real, South San Francisco, CA 94080-3299, USA*

**052 How Could The Treatment Of Soft Tissue Sarcoma Be Improved On A Regional Basis?**

Glencross J[1], Silcocks P[2], Robinson MH[3]
*[1]Leicestershire Health Authority, [2]Trent Cancer Registry, and [3]YCR Department of Clinical Oncology, Weston Park Hospital, Sheffield, S10 2SJ, UK*

**053 Indications And Advantages Of Free Flaps In The Management Of Soft Tissues Sarcomas: A Retrospective Study Of 22 Cases**

Bonvalot S, Mamlouk K, Kolb F, Le Pœchoux C, Le Cesne A
*Department of Surgery, Institut Gustave Roussy, Villejuif, France*

**054 Aggressive Fibromatosis. A Retrospective Study Of 72 Patients**

Sørensen A, Keller J, Nielsen OS, Jensen OM
*Centre for Bone and Soft Tissue Sarcomas, University Hospital of Aarhus, Aarhus, DK-8000, Denmark*

**055 Adult Pelvic Sarcomas**

Dekker C[1], Houtkarnp R[1], Peterse JL[2], van Coevorden F[1]
*[1]Department of Surgical Oncology and [2] Department of Pathology, The Netherlands Cancer Institute/Antoni van Leeuwenhoek ziekenhuis, The Netherlands*

**056 Liver Resection For Metastatic Soft-tissue Sarcomas**

van Ruth S, Mutsaerts E, van Coevorden F
*Department of Surgery, Netherlands Cancer Institute / Antoni van Leeuwenhoek Hospital, Amsterdam, The Netherlands*

**057 Surgery For Sarcoma Of The Spine: Outcome Analysis Of 59 Patients Over A 12-year Period**

Patrick Boland, Mark Bilsky, Murray Brennan, Robert Woodruff, John Healey
*Memorial Sloan Kettering Cancer Center, NY, NY, USA*

**063 The Use Of Cemented Allografts For Reconstruction Of Segmental Bone Defects After Tumour Resection**

Gerrand CH, Griffin AM, Davis AM, Wunder JS, Bell RS, Gross AE
*University Musculoskeletal Oncology Unit, Mount Sinai Hospital, Toronto, Ont., Canada M5G 1X5*

**068 Treatment Of Non-resectable Soft Tissue Sarcoma Of The Limbs By Isolated Limb Perfusion**

Vaglini M, Gronchi A, Pennacchioli E, Deraco M, Baratti D, Bertulli R, Casali PG, Lozza L, Rasponi A, Dileo P, Pilotti S, Azzarelli A
*Istituto Nazionale Tumori, Milan, Italy*

**069 Soft Tissue Sarcomas Of The Hand And Foot**

Lin PP, Guzel VB, Pisters PWT, Zagars GK, Yasko AW
*University of Texas M.D. Anderson Cancer Center, 1515 Holcombe Blvd., Houston TX 77030, USA*

**070 Long-term Follow-up Of Giant Cell Tumor Of The Sacrum Treated With Selective Arterial Embolization And Other Modalities**

Lin PP, Guzel VB, Moura MF, Morello FA Jr, Benjamin RS, Gokaslan ZL, Weber KL, Yasko AW
*University of Texas MD Anderson Cancer Center Box 106, Houston, TX 77030, USA)*

**071 Survivorship Of Segmental Prosthetic Arthroplasty For Limb Salvage Following Bone Sarcoma Resections**

Yasko AW, Lin PP, Weber KL
*The University of Texas M. D. Anderson Cancer Center, Houston, TX 77030, USA*

**072 Pathologic Fractures In Osteosarcoma**

Saghieh SS, Lin PP, Weber KW, Jaffe N, Patel SR, Benjamin RS, Yasko AW
*The University of Texas M. D. Anderson Cancer Center, Houston, TX 77030, USA*

**080 Combined Surgery, Brachytherapy And External Irradiation Of Locally Advanced Soft Tissue Sarcomas: A Feasible Approach**

Pedersen JG[1], Krarup-Hansen A[2], Daugaard S[3], Höjlund B[4], Rosendal F[2], Larsen jS[2], Lund B[1], Engelholm SA[2]
*[1]Department of Orthopaedic Surgery, [2]Department of Radiation Oncology, [3]Department of Pathology, and [4]Department of Radiology, National University Hospital (Rigshospitalet), DK-2 100 Copenhagen, Denmark*

**065 Combined Therapy For Retroperitoneal Soft Tissue Sarcomas: Pre-operative Radiation And Surgery**

Gronchi A, Azzarelli A, Baratti D, Lombardi F, Gandola L, Navarria P, Bertulli R, Casali PG, Pennacchioli E, Rasponi A, Dileo P, Pilotti S
*Istituto Nazionale Turnori, Milan, Italy*

**066 Chordoma: Natural History And Treatment Results In 55 Patients**

Gronchi A, Azzarelli A, Baratti D, Lozza L, Bertulli R, Casali PG, Pennacchioli E, Rasponi A, Dileo P, Pilotti S
*Istituto Nazionale Tumori, Milan, Italy*

**087 PERIOSTEAL CHONDROSARCOMA: EXPERIENCE OF THE ISTITUTO ORTOPEDICO RIZZOLI**

Fabbri N, De Paolis M, Vanel D, Mercuri M, Picci P
*Department of Musculoskeletal Oncology, Istituto Ortopedico Rizzoli – Bologna, Italy*

**088 Desmoplastic Fibroma: Experience Of The Istituto Ortopedico Rizzoli**

Fabbri N, Trentani F, Vanel D, Mercuri M, Picci P, Bertoni F
*Department of Musculoskeletal Oncology, Istituto Ortopedico Rizzoli – Bologna, Italy*

**089 HEMANGIOPERICYTOMA OF BONE: THE RIZZOLI EXPERIENCE**

Campanacci L, Fabbri N, Vanel D, Mercuri M
*Department of Musculo Skeletal Oncology, Istituto Ortopedico Rizzoli, Bologna, Italy*

**090 SOFT-TISSUE SARCOMA (STS) — ADHERENCE TO GUIDELINES**

Nijhuis PHA[1], Schaapveld M[2], Otter R[2], Hoekstra HJ[1]
*[1]Department of Surgical Oncology, Groningen University Hospital, Groningen, The Netherlands, and [2]Comprehensive Cancer Center North-Netherlands, Groningen, The Netherlands*

**091 Pre-operative Radiation Therapy In The Treatment Of Soft Tissue Sarcomas**

Virkus, Mollabashy A, Reith JD, Berrey HB, Zlotecki RA, Scarborough MT
*University of Florida, Gainesville, FL 32610, USA*

**095 Outcome Following Local Recurrence In Osteosarcoma**

Grimer RJ
*The Royal Orthopaedic Hospital Oncology Service, Bristol Road South, Birmingham, UK*

**107 Combined Surgery, Brachytherapy And External Irradiation Of Locally Advanced Soft Tissue Sarcomas: A Feasible Approach**

Pedersen JG[1], Krarup-Hansen A[2], Daugaard S[3], Hojlund B[4], Rosendal F[1], Larsen JS[2], Lund B[1], Engelholm SA[2]
*Departments of [1]Orthopaedic Surgery, [2]Radiation Oncology, [3]Pathology, and [4]Radiology, National University Hospital (Rigshospitalet), DK-2100 Copenhagen, Denmark*

**109 Unplanned Excision For Soft Tissue Sarcoma: Identification Of A Subgroup With Bad Prognosis**

van Geel AN, Eggermont AMM, Schmitz PIM
*Department of Surgical Oncology and Statistics, University Hospital Rotterdam/Daniel den Hoed Cancer Center, Rotterdam, The Netherlands*

### Radiation Oncology

**029 Potential Impact Of High Technology Radiation Treatment For Sarcomas To Improve Patient Outcome**

Herman Suit, Karen Doppke, Jong Kung, J. Michael Collier, Ira Spiro, Thomas Delaney
*Department of Radiation Oncology, Massachusetts General Hospital, Harvard Medical School, Boston, MA, USA*

**031 Effects Of Irradiation And Radioprotectant On Rat Growth Plate Morphology**

Damron TA, Spadaro JA, Farnum CE, Margulies BS, Strauss J
*Upstate Medical University Department of Orthopedic Surgery, Syracuse, NY 13224, USA*

**032 Bone Density Effects Of Radiation And The Radioprotectant Drug Amifostine In A Rat Model**

Morgan H, Damron TA, Margulies B, Spadaro J
*Upstate Medical University Department of Orthopaedic Surgery, Syracuse, NY 13224, USA*

**033 Pattern Of Local Recurrence After Conservative Surgery And Radiotherapy For Soft Tissue Sarcoma**

Cleator SJ, Cottrill C, Harmer CL
*Sarcoma Unit, Royal Marsden Hospital NHS Trust, London SW3 6JJ, UK*

**036 Treatment Outcome In Patients Radiated For Spinal And Paraspinal Tumors**

DeLaney TF, Ancukiewicz M, Hornicek FJ, Spiro IJ, Harmon DC, Suit HD
*Massachusetts General Hospital, Boston, MA 02114, USA*

**038 Pre-operative Radiotherapy In Adult Soft-tissue Sarcomas**

Morysinski T, Ruka W, Rutkowski P
*Soft Tissue/Bone Sarcomas Department, M. Sklodowska-Curie Memorial Cancer Center Institute, Warsaw, Roentgena 5, Poland*

**098 Malignant Peripheral Nerve Sheat Tumors (Mpnst): A Retrospective Analysis On 125 Patients**

Voican D, Billard C, Terrier P, Bonvalot S, Vanel V, Le Pøchoux C, Missenard G, Le Deley MC, Le Cesne A
*Soft Tissue Sarcoma Unity, Institut Gustave Roussy (IGR), Villejuif 94805, France*

**099 Is Adjuvant Radiation Therapy (Rt) Indicated After Optimal Resection For Soft Tissue Sarcoma (Sts) Of The Extremities?**

Le Cesne A, Khanfir K, Terrier P, Alzieu L, Bonvalot S, Vanel D, Le Pøchoux C
*Soft Tissue Sarcoma Unity, Institut Gustave Roussy, Villejuif 94805, France*

### Basic Science/biology

**001 Insulin And Igfs Signaling In Soft Tissue Sarcomas: Role Of Irs-1 And Irs-2 Proteins**

Bogetto L, Cervi M, Spessotto P, Mucignat MT, Canzonieri V, De Paoli A, Perris R, Colombatti A
*Centro di Riferimento Oncologico — National Cancer Institute, Aviano 33081, Italy*

**002 Ecm Interactions And Production Of Proteolytic Enzymes In Malignant Fibrous Histiocytomas**

Spessotto P, Mucignat MT, Wasserman B, Burino I, Bogetto L, Cigana A, Frustaci S, Perris R, Colombatti A
*Centro di Riferimento Oncologico, National Cancer Institute, Aviano 33081, Italy*

**008 Doxorubicin And Valspodar (Psc 833) Combined Therapy In Canine Osteosarcoma According To P-glycoprotein Expression**

Cagliero E[1], Scotlandi K[2], Morello E[3], Manara MC[2], Buracco P[3], Baroetto R[1], Ferracini R[1]
*[1]Istituto per la Ricerca e la Cura del Cancro, Candiolo 10060, Italy, [2]Istituti Ortopedici Rizzoli, Bologna 40136, Italy, and [3]Dipartimento di Patologia Animale,Torino 10126, Italy*

**104 P16INK4A Gene And Osteosarcoma**

Benassi MS, Molendini L, Gamberi G, Merli M, Ragazzini P, Magagnoli G, Picci P
*Laboratory of Cancer Research, Rizzoli Institute, 40100 Bologna, Italy*

**Intramural Oxygen Status In Soft Tissue Sarcoma: Prognostic Value With Respect To Different Treatment Modalities**

Peter Hohenberger, Mathias Schlafke, Birger Bida
*Divison Of Surgery and Surgical Onocolgy, Charite, Humboldt University of Berlin, Germany*

**Medical Oncology**

**010 Results Of A T10-derived Protocol Based On Pre-operative High-dose Methotrexate With Individual Dose Adjustment In The Treatment Of Primitive Osteosarcoma**

Duffaud F, Digue L, Volot F, Bouvier C, Curvale G, Poitout D, Favre R
*Hôpital La Timone, Marseille 13385, France*

**013 Preliminary Results Of A Phase 2 Trial Of Oral Piritrexim In Patmients With Malignant Fibrous Histiocytoma (Mfh)**

Patel SR, Jenkins J, Hays C, Beech J, Burgess MA, Papadopoulos NE, Plager C, Pisters PWT, Feig BW, Hunt K, Pollock RE, Benjamin RS
*The Sarcoma Center, University of Texas M.D. Anderson Cancer Center, 1515 Holcombe Blvd., Houston, TX 77030, USA*

**014 Extraskeletal, Adult Ewing Family Of Tumors (Eft): Combined Treatment In 42 Patients**

Casali PG, Bertulli R, Santoro A, Lombardi F, Spreafico C, Dileo P, Gandola L, Navarria P, Gronchi A, Quagliuolo V, Valente M, Brega Massone P, Mastore M, Rolfo CD, Rasponi A, Cataldo I, Pilotti S, Azzarelli A
*Istituto Nazionale Tumori, Milan, Italy*

**015 Aggressive Fibromatosis: A Conservative Approach To Inoperable Disease Based On Low-dose Chemotherapy With Methotrexate + Vinblastine**

Azzarelli A, Gronchi A, Bertulli R, Casali PG, Lozza L, Baratti D, Pennacchioli E, Rasponi A, Dileo P, Rolfo CD, Pilotti S
*Istituto Nazionale Tumori, Milan, Italy*

**016 Metastatic Angiosarcoma – Recent Treatment Results**

Harmon DC
*Massachusetts General Hospital, Boston, MA 02114, USA*

**078 Expression Of Fas Ligand And Fas In Human Soft Tissue Sarcomas Before And After Hyperthermic Isolated Limb Perfusion With Tnfα And Melphalan**

Komdeur R, Molenaar WM, de Jong S, Hoekstra HJ, Plaat BEC, Van den Berg E, Van der Graaf WTA
*University Hospital Groningen, 9700 RB, The Netherlands*

**085 Influence Of Locally Advanced And Recurrent Disease On Survival Of Patients With Advanced Soft Tissue Sarcomas. A Retrospective Analysis Of The Eortc Soft Tissue And Bone Sarcoma Group (Stbsg)**

Reichardt P, Van Glabbeke MM, Mouridsen HT, Verweij J, Radford J, Rodenhuis S, Azzarelli A, Le Cesne A, Buesa J, Keizer HJ, van Oosterom A, Kirkpatrick AL, Nielsen OS
*EORTC Soft Tissue and Bone Sarcoma Group and EORTC Data Center, Brussels, Belgium*

**086 Chemotherapy For Metastatic Chondrosarcoma Of Bone: Single Institution Experience In 14 Patients**

Pink D, Tilgner J, Dörken B, Reichardt P
*Department of Hematology, Oncology and Tumorimmunology, Robert-Rössle-Klinik, Universitätsklinikum Charitø, Humboldt-University, 13122 Berlin, Germany*

**097 Osteosarcoma Over The Age Of 40**

Grimer RJ
*on behalf of the European Musculoskeletal Oncology Society, The Royal Orthopaedic Hospital Oncology Service, Bristol Road South, Birmingham B31 2AP, UK*

**110 Feasibility Of Pre Or Post-operative Combined Chemotherapy And Radiation-therapy In Adult Soft Tissue Sarcomas Of Extremities**
Frustaci S[1], Buonadonna A[1], Boz G[1], Berretta M[1], Rupolo M[1], Bertola G[1], Innocente R[1], Gherlinzoni F[2], De Paoli A[1]
*[1]CRO-IRCCS, National Cancer Institute, Aviano, and [2]Orthopedic Division, General Hospital, Gorizia*

### Pediatric Oncology

**081b Strategy Of Treatment In Desmoid Tumors (Aggressive Fibromatosis In Children And Adults): To Avoid Absolutely Radiotherapy**
Delepine N, Alkallaf S, Markowska R, Cornifie H, Belarbi L, Delepine G
*Oncologic Paediatric Unit, Avicenne University Hospital, 125 Bd de Stalingrad, 93009 Bobigny Cedex, France*

**081 Treatment Of Pelvic Ewing's Sarcoma With Multidisciplinary Treatment**
Delepine N[1], Delepine G[1], Delepine F[2], Alkallaf S[1], Cornille H[1], Sokolov T[3]
*[1]Oncologic Paediatric Unit, Avicenne University Hospital, 125 Bd de Stalingrad, 93009 Bobigny Cedex, France, and [2] Bone Tumor Center, Sofia, Bulgarie, and [3]CHU de Rouen, Orthopaedic Department, France*

### Diagnostic Imaging/pathology

**024 Biological Characterisation Of Soft Tissue Sarcomas**
Nielsen MM[1], Hansen O[1], Starklint H[2], Knoop A[1], Rose C[3]
*[1]Department of Oncology, Odense University Hospital, DK-5000 Odense C, Denmark, [2]Department of Pathology, Vejle Hospital, and [3]Department of Oncology, Lund University Hospital, Sweden*

**017 Good Pathological Response To Neoadjuvant Chemotherapy In Patients Showing Clinical Or Radiological Progression Of Disease – 3 Clincal Cases**
Plummer R, Lucraft H, Murray S, Malcolm A, Dildey P, Grainger A, Baudouin C, Verrill M
*North of England Bone and Soft tissue Tumour Service, Freeman Hospital, Newcastle Upon Tyne, NE7 7DN, UK*

**018 Malignant Fibrous Histiocytoma Arising Within A Solitary Osteochondroma**
Carluke I, A Grainger, Murray SA, Malcolm AJ

**The North of England Bone and Soft Tissue Tumour Service, The Freeman Hospital, Newcastle upon Tyne NE7 7DN, UK**

**019 Sacrococcygeal Soft Tissue Ependymoma**
Carluke I, Murray SA, Malcolm AJ
*The North of England Bone and Soft Tissue Tumour Service, The Freeman Hospital, Newcastle upon Tyne NE7 7DN, UK*

**020 Tissue Microarray Blocks: An Efficient Method For Screening Soft Tissue And Bony Sarcomas**
Thomas D, Baker L, Giordano T
*University of Michigan Comprehensive Cancer Center, Ann Arbor, MI 48109, USA*

**021 Myofibroblast Modulation In Desmoid Tumors**
Baldini N, Barbanti-Brodano G, Zini N, Bertoni F, Giunti A
*Istituti Ortopedici Rizzoli, 40136 Bologna, Italy*

**023 Her2/neu Expression In Osteosarcomas And Other Bony Sarcomas**
Thomas D, Giordano T, Sanders D, Arrowsmith P, Baker L
*University of Michigan Comprehensive Cancer Center, Ann Arbor, M1 48109, USA*

**074 Matrix Gene Expression And Cellular Phenotyping In Chondroid Chordoma Reveals Focal Maturation Of Neoplastic Chordoid Cells Mimicking Histogenesis Of Developing Nucleus Pulposus**
Gottschalk D, Fehn M, Patt S, Saeger W, Kirchner T, Aigner T
*Institute of Pathology, FAU Erlangen-Nürnberg, Germany*

**093 Host Immune Response In Osteosarcoma**
Casanova J, Reith JD, Berrey BH, Enneking WF, Scarborough MT
*University of Florida, Gainesville, FL 32610, USA*

**094 Molecular Predictors Of Outcome In Patients With Osteosarcoma**
Reith JD, Casanova J, Berrey BH, Enneking WF, Scarborough MT
*University of Florida, Gainesville, FL 32610, USA*

**103 A Case Of Gastro Intestinal Sarcoma Tumor (Gist) Revealed By A Mediterranean Kaposi's Sarcoma In An Hiv-negative Patient: Casual Association Or Not?**
Vincent Baty[1], Isabelle Ray-Coquard[1], Eric Fontaumard[1], Dominique Ranchere-Vince[2], David Tavan[1], Jean-Yves Blay[2]
*[1]Clinique Eugene Andre, and [2]Centre Leon Berard, Lyon, France*

### Oncology Research

**009 Hypoxia In Human Soft Tissue Sarcomas: Adverse Impact On Survival And No Association With P53 Mutations**
Nordsmark M[1,2], Alsner J[1], Keller J[3], Nielsen OS[2], Jensen OM[4], OvergaardJ[1]
*[1]Department of Experimental Clinical Oncology, [2]Department of Oncology, [3]Department of Orthopaedic Surgery and [4]Department of Pathology, Danish Cancer Society, Aarhus University Hospital. Nörrebrogade 44, bldg 5, DK-8000 Aarhus C, Denmark*

**101 The Functional And Local Results Of Limb Sparing Procedures In Upper Girdle Neoplasms Treatment**

Dardzinski R, Ruka W, Koziol A, Rutkowski P Maria
*Sklodowska–Curie Memorial Cancer center, Warshaw, Poland*

**102 Lymphonpenia (Ly) As An Independent Prognostic Factor For Survival In Advanced Soft Tissue Sarcomas As Well As In Lymphomas, And Metastatic Breast And Renal Carcinoma**

Blgy J, Ray-Coquard I, van Glabbeke M, Nøgrier S, Saghatchian M, Verweij J, Sebban C, Escudler B, Guastalla J-P, Judson I, Nielsen OS, Biron P, Le Cesne A, Centre L. Børard, H.Ed. Herriot, Lyon, I.G. Roussy, Villejulf, France, and EORTC STSSG
*Bruxelles, Belgium*

**Posters — Surgical Treatment Of Sarcomas**

**025 The Predictive Value Of Tissue Tran-glutaminase In Malignant Fibrous Histiocytoma**

Heiner JP, Hughes EM, Aeschlimann D, Hafez R, Kehoe R
*University of Wisconsin, Madison, WI 53792, USA*

*Introduction:* Transglutaminase is a family of calcium dependent enzymes that act by catalyzing the cross-linking of proteins during the formation of *N*-glutamyl-lysyl bond between two peptide chains, and through G protein mediated activation in normal tissues. Great interest in tTGase has come about because of its implicated role in apoptosis, cell adhesion, tumor growth and differentiation and invasive/metastatic behavior.

Malignant fibrous histiocytoma (MFH) is thought to be the most common soft tissue sarcoma of late adult life. Although several other factors have also been investigated, prognosis for these tumors has been largely based on size of tumors and grade. Initial treatment is typically wide surgical resection and radiation therapy. The aim of our study was to determine if tTGase has predictive value for metastatic disease or local recurrence in MFH to better identify those patients that may benefit from adjuvant chemotherapy.

*Methods:* This is a retrospective study using pathologic specimens previously obtained from 1988–1999 in patients diagnosed with MFH. Twenty-three patients in this time period for which we have adequate non-irradiated tissue are included in the study. Thirteen subjects were male, nine were female. Paraffin-fixed pathologic specimens were collected, underwent deparafination and immunohistochemical staining as previously described by Aeschliman.[1] After staining with the monoclonal antibody was complete, the stains for each tumor were graded on a 0–3 scale by two separate observers, blinded to each other's results.

A retrospective review was performed of patients' charts as well as through telephone contact for those patients that had limited follow-up. Information on size of tumor, histologic grade, dates of recurrence of metastatic disease and death were recorded. Biostatistical analysis was performed to evaluate the relationship of size, grade, age and tTGase average rating to disease free survival. This was performed in a multivariate and univariate fashion utilizing Kaplan–Meier regression curves.

*Results:* Average follow-up was 40 months (range, 4–126 months). Eight out of 23 had no local recurrence or metastatic disease, 2/23 had local recurrence, and 14/23 had metastatic disease. Univariate analysis demonstrated no statistically significant difference for disease free survival based on age or histologic grade. Disease free survival for those with a size of 10 cm was significantly shorter ($p < 0.01$) with a median disease free survival of ~3 months versus ~3 years for those < 10 cm. Additionally, those with a higher (> 1) tTGase average score had a significantly shorter ($p \sim 0.02$) disease free survival of ~4 months versus 2 years for those ≤ 1. However, when tTgase was corrected for tumor size, a trend ($p \leq 0.09$) was seen for tTgase score in multivariate analyses.

*Discussion:* Tissue transglutaminase has been shown to be involved in programmed cell death, cell matrix stabilization and cell adhesion. We found that a higher tTgase ($p \sim 0.02$) may be associated with a poorer outcome. These people may benefit from adjuvant chemotherapy but otherwise would not have previously been identified to be in a higher risk group. A larger study with more statistical power is needed to determine if the association found here is significant.

**041 Pathologic Fracture In Osteosarcoma: Prognostic Significance And Treatment Implications**

Ghert MA, Zurakowski D, Gebhardt MC, Thompson RC, Scully SP
*Duke University Medical Center, Durham, NC 27710, USA*

*Introduction:* The presence of a pathologic fracture in an osteosarcoma has been considered a poor prognostic factor and an indication for immediate amputation in many tumor centers. The purpose of this study was to determine, in the current era of neoadjuvant chemotherapy, if the presence of a pathologic fracture in an osteosarcoma has prognostic significance, and if limb salvage can be safely performed in these patients without compromising clinical outcome.

*Methods:* In a cooperative effort of the Musculoskeletal Tumor Society (MSTS), members from eight institutions provided retrospective data on 58 patients treated for a pathologic fracture in an osteosarcoma, and 55 patients treated for an osteosarcoma without pathologic fracture. The two groups were matched for tumor location and grade. The following variables were analyzed: age, gender, stage of disease, anatomic location, tumor size, pathological fracture, fracture union, fracture displacement, tumor management (limb salvage versus amputation), chemotherapy, surgical margin, vascular invasion and percent necrosis. Survival analysis was performed with the Kaplan–Meier product-limit method with variables having a $p$ value < 0.10 based on the log-rank test entering into the multivariate Cox proportional-hazards regression model. Outcomes examined were survival, disease-free survival, local recurrence and distant metastasis. Multiple stepwise logistic regression analysis was used to compare the outcome of limb salvage versus amputation with respect to local recurrence.

*Results:* The median age of the 113 patients was 17.4 years (range, 2–69 years). Mean follow-up was 4.0 years (range, 6 months–12.7 years). Ninety-five percent of the patients presented with MSTS stage IIB disease. All but eight patients underwent induction chemotherapy. Of the 58 patients with a pathologic fracture, 32 were managed with immobilization, six with internal fixation, and 16 with amputation. Overall mortality was 35%, with 14 patients (12%) suffering a local recurrence. All patients who experienced a local recurrence died of the disease process. The presence of a pathologic fracture was a significant univariate factor associated with decreased overall survival ($p = 0.03$), disease-free survival ($p = 0.03$), increased local recurrence ($p = 0.005$) and distant metastasis ($p = 0.03$). Multivariate analysis revealed that the presence of a pathologic fracture was a significant risk factor for local recurrence ($p = 0.003$). In the group with a pathologic fracture, 10/41 (24%) treated with limb salvage and 1/17 (6%) treated with an amputation suffered a local recurrence. Stepwise multiple logistic regression revealed that limb salvage was a multivariate risk factor for local recurrence in the pathologic fracture group, with those patients undergoing limb salvage estimated to be nine times more likely to develop local recurrence than those undergoing amputation (odds ratio, 9.0; 95% confidence interval, 5.0–35.5; $p = 0.01$).

*Discussion and conclusion:* The presence of a pathologic fracture in an osteosarcoma is an independent predictor of local recurrence with limb salvage significantly increasing the risk. Given the fact that recurrence in an osteosarcoma has a uniformly fatal outcome, our data indicate that the presence of a pathologic fracture may be a contraindication to limb salvage in these patients.

**044 Thallium 201 Uptake In Chondroid Lesions**

Templeton KT, Raveill T, Tawfik O
*University of Kansas Medical Center, Kansas City, KS 66160, USA*

Heterogeneity seen in cartilagenous lesions can make grading and surgical planning difficult. Low-grade lesions may potentially be treated with less aggressive surgery. However, if higher-grade lesions are missed, the local recurrence rate can be high. In conjunction with standard imaging modalities, thallium 201 scans have been utilized in the evaluation of tumors to distinguish benign from malignant lesions. In chondroid lesions, thallium 201 may help to differentiate low- from high-grade lesions.
*Methods:* Thallium 201 scans were utilized to evaluate 25 patients (15 females, 10 males) who had cartilagenous lesions noted on standard radiographs. Four patients were felt to have low-grade lesions on open biopsy. Thallium 201 scans were obtained to evaluate for the presence and location of higher-grade areas in these lesions. thallium 201 (5 mCi) was injected, with images obtained early (20 minutes) and late (3 hours). SPECT imaging was used for pelvic lesions. Eighteen patients underwent either surgical excision/wide resection if they had positive scans or curettage if their scans were negative but they remained symptomatic. The remainder of the patients was followed with routine clinical and radiographic exams.
*Results:* The areas noted on histologic exam of the operated lesions to be benign or grade I malignancies demonstrated an average thallium 201 uptake (compared with the normal contralateral side) of $1.005 \pm 0.125$ (range, 0.90–1.10). This was unchanged on delayed images. All have been followed radiographically and clinically for over 12 months, without evidence of recurrence. In comparison, those patients found to have grade II or III lesions histologically had mean uptake ratios of $1.485 \pm 0.255$ (range, 1.26–1.64) cts/pixel compared with an equivalent anatomic location on the contralateral normal side ($p = 0.0001$). All four patients who were felt to have low-grade lesions on initial biopsy had thallium 201 ratios of greater than 1.3 in some area of their tumors. All underwent wide resection and were found to have grade II–III lesions. In addition, seven other patients with chondroid lesions diagnosed radiographically and having negative thallium scans have been followed with serial clinical exams and roentgenograms. All of these patients remain asymptomatic and with no change in their radiographs (follow-up, 4 months–5 years).
*Conclusion:* Thallium 201 uptake, mediated through the Na-K ATPase dependent pump, is dependent not only on blood flow, but also on tumor cell viability and activity. A greater degree of uptake is seen in more active regions of tumors. With the heterogeneity seen in chondroid lesions, Thallium 201 may help elucidate those tumors, or regions within tumors, that are more aggressive and, therefore, need more aggressive surgical management.

**045 Complications And Morbidity Of Adjuvant Therapy For Soft Tissue Sarcoma**

Templeton KT
*University of Kansas Medical Center, Kansas City, KS, USA*

Although adjuvant therapy, combined with surgery, can lead to local tumor control and salvage of extremity, post-operative complications can delay further treatment and, potentially, lead to loss of the extremity. This study was designed to analyze those factors that may contribute to post-operative morbidity.
*Methods:* The records of 45 patients presenting with extremity soft tissue sarcomas were reviewed retrospectively. Five patients were unable to undergo limb salvage, leaving 40 patients that form the basis of this study. These patients all underwent surgical resection of their tumors in combination with radiation (pre- or post-operatively) and/or chemotherapy. They were then followed for at least 3 months to evaluate for post-operative complications. The complications were defined as major (i.e. requiring further surgery) or minor (managed non-operatively).
*Results:* There were 24 female and 16 male patients with an average age of 55 years. The most common diagnoses were MFH and liposarcoma. The most common locations for the tumor were thigh and forearm. Thirty-one patients received radiotherapy, 21 pre-operatively (six with a post-operative boost) and 10 post-operatively only. There were four minor complications, usually slough of a skin graft, and 16 major complications. The most frequent major complications were seromas, primarily in those patients treated initially with a free tissue transfer into the resection bed, and dehiscence, seen in those patients closed primarily. Two free tissue transfers underwent necrosis, presumably due to the anastomoses being performed into abnormally large vessels adjacent to the tumor, which eventually decreased in caliber. No complication resulted in an amputation. Patient factors that were analyzed and found not to be related to major wound complications were age, gender, obesity (measured by body mass index), nutrition (measured by white cell count and serum albumin), and smoking. Treatment factors that were not related to complications were the use of chemotherapy, radiation dose, and interval between pre-operative radiation and surgery. However, those receiving pre-operative radiation had significantly more complications than those treated with post-operative RT alone. Furthermore, those with a larger estimated volume of resection, especially if treated with pre-operative RT, had a higher incidence of complications. Other surgical factors that correlated with wound complications were the width of resection (most likely affecting the vascularity of the operative bed), estimated volume of resection (although modified if free tissue transfer was performed), and estimated blood loss. Total operative time was not significant. Another independent risk factor for complications was an incisional or excisional biopsy performed at another institution prior to presentation (56% complications versus 29%). This was not due to increased resection volume as the volume of tissue ultimately resected was lower (944.7cm$^3$ versus 1888 cm$^3$) for those initially treated elsewhere.
*Conclusions:* Post-operative morbidity appears to be relatively independent of the patient factors analyzed herein. Morbidity is more likely to be determined by treatment and tumor-related factors, such as tumor and therefore, resection volume and width, estimated blood loss, and prior surgery. These factors may lead to relative hypoxia in the resection bed.

**046 Osteochondroma Of The Femoral Neck**

Siebenrock KA, Ganz R
*Orthopaedic Surgery, University of Berne, 3010 Berne, Switzerland*

Osteochondromas of the femoral neck represent rare intraarticular lesions. The difficulty to obtain adequate surgical access, especially when located at the inferior and posterior femoral neck, has been expressed already in the literature. The authors have approached these lesions with a new, versatile approach that offers the advantage of complete tumor visualization, circumferential access to the femoral neck combined with the possibility of entire hip joint inspection, and intraoperative control of adequate tumor resection. The surgical approach includes a digastric trochanteric osteotomy and subluxation or even dislocation of the femoral head, thereby respecting the crucial blood supply to the femoral head. This approach has been used in four patients with osteochondroma of the femoral neck presenting with pain, restricted range of motion and limping. Femoro-acetabular impingement of the bulky osteochondroma against the acetabular rim could be verified in all cases. In two patients, labral lesions were found at the impingement site. All four patients had complete or significant relief of symptoms at a mean follow-up of 34 months (18–48 months) without signs of progressive osteoarthritis or avascular necrosis.

### 047 Surgical Margins Influence Local Recurrence Of Soft Tissue Sarcomas In The Thigh, But Not Survival In 152 Pateents

Vraa S, Keller J, Nielson O, Jurik AG, Jenson OM
*Center for Bone and Soft-Tissue Sarcomas, University Hospital of Aarhus, DK-8000 Arhus C, Denmark*

In a 19-year period (1979–1998), 152 consecutive patients with soft tissue sarcomas in the thigh were surgically treated in the Sarcoma center in Aarhus. Prognostic clinicopathologic factors for local recurrence and survival were studied by use of multivariate statistical analysis. Twenty-seven patients (18%) had a low-grade tumor, 26 (17%) an intermediate-grade tumor, and 99 (65%) a high-grade tumor.[1] Twenty-seven patients (18%) were amputated and 125 (82%) had local resection. Twenty-one patients (14%) underwent a marginal resection, 82 patients (54%) had a wide resection, and 49 (32%) had a compartmental resection. Fourteen patients (9%) developed local recurrence. All patients but one had the local recurrence surgically removed. The patients with local recurrence had a significantly poorer survival compared with patients without local recurrence. Forty-nine patients (32%) developed distant metastases. The 5-year recurrence-free rate was 91%. The multivariate analysis selected marginal resection (versus wide and compartmental resection) and histological-high grade (versus intermediate and low-grade) as unfavorable prognostic factors for local recurrence. The 5-year survival rate was 68%. High age (versus age less than median age) and hitological high grade (versus intermediate and low-grade) were selected as unfavorable prognostic factors for survival in a multivariate analysis. In the present study, surgical margin influenced local recurrence but not the overall survival in sarcomas in the thigh.

#### Reference

1. Jensen OM, *et al*. Histopathologica grading of soft tissue tumours, prognostic significance in a prospective study of 278 consecutive patients. *Pathology* 1991; 163:19–24.

### 048 Soft Tissue Sarcomas After 80 Years Of Age. Study Of A Series Of 22 Patients

Turcotte R, Barabas D, Isler M, Doyon J, Normandin D
*Division of Orthopaedic Surgery, Maisonneuve-Rosemont Hospital, Montreal Que., Canada H1T 4B3*

*Aim of the study:* The management of older patients who have soft tissue sarcomas (STS) is one of the most challenging problems in oncology. The aim of this retrospective study was to report the results of the treatment for the STS in 22 patients older than 80 years of age.
*Patients and methods:* Twenty-two patients (10 men and 12 women) with STS treated between 1991 and 1999 in our department were studied. The histologic appearance and grade were correlated with subsequent treatment and clinical behavior.
Based on the type of therapy, four groups were analyzed: in the first group, patients were treated exclusively by surgical resection (four patients); in the second group, patients were treated only by radiotherapy (two patients); in the third group, surgical resection was completed by radiotherapy (12 patients); and, finally, the fourth group patients without therapy (four patients).
*Results:* The patients age at diagnosis ranged from 80 to 91 years (average, 84.8 years). Tumors were located in the lower limb in 14 patients, in the upper limb in seven patients, and in the back in one patient. The histologic types of tumor were the following: malignant fibro-histiocytoma (10), leiomyosarcoma (7), liposarcoma (3), synovia-sarcoma (1), and malignant schwannoma (1). Nineteen patients had a histologically high-grade tumor (grade III 15/22 and grade IV 4/22). Three patients had a low-grade tumor (grade II). The mean survival of patients after diagnosis was 26.3 months (range, 1–113 months). Ten patients died during the follow-up, four deaths were related to the disease and six were not. Metastases were found in six patients, in four of them at the time of diagnosis. In the first group (surgically treated patients), the mean survival was 15.5 months and one patient died of unrelated cause. Local recurrence occured in one patient. In the second group (two patients with radiotherapy only), the mean survival was 5.5 months, both patients developped diffuse metastases and both died. In the third group (12 patients with surgical resection and radiotherapy), the mean survival was 31.3 months. Six patients died: one of lung metastasis and five death were not related with the tumor. Three local reccurrences occurred. In the fourth group (four patients without treatment), all patients died; three of them related to the disease and one of unrelated cause. The mean survival was 3 months.
*Conclusion:* Patients older than 80 years with STS remain a high-risk group. Nevertheless, age is not a limitating factor in the surgical treatment of STS. These data suggest that an aggressive approach including surgical resection and radiotherapy is appropriate in management of older patients with STS.

### 049 Primary Leiomyosarcoma Of Bone. A Clinicopathologic Study Of 10 Patients

Barabas D, Turcotte R, Isler M, Doyon J, Normandin D
*Division of Orthopaedic Surgery, Maisonneuve-Rosemont Hospital, Montreal, Que., Canada H1T 4B3*

*Backround and objectives:* Primary leiomyosarcoma of bone (PLMSB) is a very rare malignant tumor with uncertain pathogenicity. In order to improve our understanding of pathological behaviour, prognostical predictors and treatment of this entity, we proposed to review all PLMSB surgically treated in our department.
*Methods and results:* We retrospectively reviewed 10 patients (five men and five women) with PLMSB treated between 1991 and 1999 in our department. The mean age was 57.2 years (30–79 years). The long bones were preferentially affected; tumor was localized in the lower limb in eight patients and in the upper extremity in two patients. At histology high-grade tumor was found in six patients. Surgical resection was performed in all patients, a limb salvage surgery being possible in nine patients. Surgical resection was completed by pre- and post-operative chemotherapy in two patients and by post-operative radiotherapy in three patients. One patient had pre-operative radiotherapy. The mean follow-up was 31.3 months (12–72 months). Five patients are alive with no evidence of disease, four patients died with disease and one patient is alive with lung metastasis. Metastases occured in four patients, mean time to metastasis was 16 months after surgery. In the four patients who died, survival averaged 25 months.
*Conclusions:* Our experience concerning the diagnosis of PLMSB is similar to the reported data. There is an unclear relationship between histology and prognosis. If metastases occur early, they are a common cause of death. Survival at 2 years is 60%. Surgical treatment is the therapy of choice. Multiagent chemotherapy and radiotherapy did not seem to provide an improved prognosis over a simple ablative procedure.

**050 Giant Cell Tumor Of The Pelvis And Sacrum: Review Of Seventeen Cases And Meta-analysis**

Leggon RE, Berry BH, Zlotecki R, Reith J, Wood D, Vlasak R, Scarborough MT
*University of Florida, Gainesville, FL 32610-0246, USA*

Seventeen patients with giant cell tumor (GCT) of the pelvis and sacrum were reviewed. The mean patient age was 33 years and average follow-up was 8.0 years. Sixteen of 17 lesions were stage 3 (benign, aggressive). Symptom duration averaged 12 months. The average largest tumor dimension was 11 cm, and one developed benign pulmonary metastases. The treatment was radiation therapy (RT) alone in nine patients, surgery with intralesional margins alone (S(IL)) in two, and surgery with wide margins S (W) in four. Primary RT in eight sacral lesions resulted in one local recurrence of tumor, four cases in which the tumor involuted and the bone reformed, and three cases that were stable but without ossification. Wide surgical margins for pelvic lesions resulted in no local recurrences. Two of 12 patients treated with RT developed radiation-induced sarcoma (RIS). Local recurrence occurred in 23% of patients in this series.

Meta-analysis yielded 239 lesions (73 pelvis, 166 sacrum). Recurrence rates were 49% for RT (32 of 65 patients), 47% for S(IL) (32 of 68 patients), 46% for S(IL)–RT (38 of 83 patients), 60% for S(IL) + cryosurgery (six of 10 patients), and 0% for S(W) (0 of 23 patients). Benign lung metastases developed in 6%, secondary malignancies in 2%, perioperative death in 2%, and multicentricity in < 1%. The average dose of RT was 4780 cGy. Radiation dosage studies of 122 cases, including 34 cases of primary RT alone, were reviewed. Increasing doses of RT (< 4500 cGy versus 4500–5500 cGy versus > 5500 cGy) did not appear to decrease the rate of local recurrence. RIS developed in 11% of patients with > 5-year follow-up. There did not appear to be a benefit to RT in addition to intralesional surgery. Disease status was worse for sacral GCT (ANED, 90%; AWD, 1%; DOD, 6%; DOC, 3%) at an average follow-up of 8.7 years.

Surgery with wide margins should be considered for GCT of the ilium, pubis, or lower sacrum to decrease the chance of local recurrence and the potential for radiation-induced sarcoma. Treatment of acetabular lesions must be individualized. Primary RT alone is most strongly indicated for large lesions of the sacrum.

**051 Recent Experience With The Compress® Device For Compliant Fixation Of Oncologic Endoprostheses**

O'Donnell RJ[1,2], Martin DL[2], Silverstein RM[2], Johnston JO[1]
[1]*Department of Orthopaedic Surgery, Orthopaedic Oncology Service, University of California, San Francisco, 1701 Divisadero St., Suite 280, San Francisco, CA 94115-1652, USA, and* [2]*Department of Musculoskeletal Oncology, The Permanente Medical Group, Inc., 1200 El Camino Real, South San Francisco, CA 94080-3299, USA*

*Purpose:* To determine the early survivorship of the ComPreSs® (CPS) prosthesis for compliant endoprosthetic fixation of massive oncologic defects.

*Methods:* A retrospective review of CPS implants was undertaken. Results with respect to prosthetic failure, local recurrence, metastatic disease, and death were studied. Radiographs were analyzed to ascertain evidence of prosthetic loosening.

*Results:* Twenty-eight consecutive cases comprising the entire experience of a single surgeon with the ComPreSs® prosthesis were reviewed. Follow-up averaged 1.43 years (range, 0.25–3.92 years). There were 18 male and 10 female patients with average age of 30 years (range, 12–68 years). There were 21 primary oncologic cases, six revision oncologic cases, and one post-traumatic case. Osteosarcoma (12 cases) was the most common diagnosis. Prosthetic locations included: distal femur (18), proximal tibia (5), distal humerus (3), and proximal femur (2).

There was only one (1/28, 3.6%) mechanical distal femoral prosthetic failure at 3 months leading to successful revision CPS surgery. There was one deep infection treated with debridement, antibiotics, and prosthetic retention. There were two local recurrences (osteosarcoma, dedifferentiated chondrosarcoma with pathologic fracture) requiring amputation at 13 and 20 months post-operatively. Both implants were firmly fixed; both patients developed metastatic disease. There were no other metastases. There were no deaths. All remaining prostheses have continuous radiographic evidence of stable osteointegration.

*Discussion:* The ComPreSs® implant has proven to be versatile, durable, and effective for primary and revision oncologic indications. The CPS design attempts to avoid problems of loosening and stress shielding encountered with both cemented and porous-coated stems conventionally used in limb salvage reconstructions. Rigid fixation at the prosthetic–bone interface prevents motion, allowing osteointegration and sealing the canal from particulate debris. High compliance afforded by the washers works to avert stress shielding. This is expected to enhance long-term prosthetic survival. A multi-center FDA IDE study of the distal femoral CPS prosthesis is now underway.

**052 How Could The Treatment Of Soft Tissue Sarcoma Be Improved On A Regional Basis?**

Glencross J[1], Silcocks P[2], Robinson MH[3]
[1]*Leicestershire Health Authority,* [2]*Trent Cancer Registry, and* [3]*YCR Department of Clinical Oncology, Weston Park Hospital, Sheffield, S10 2SJ, UK*

*Background:* Bone and soft tissue sarcomas are a rare and heterogeneous group of tumours, rarely seen by most clinicians during their careers. Early recognition, appropriate and timely investigations, and treatment are shown to markedly affect outcomes. In the Trent Region in the UK, a soft tissue sarcoma interest group had developed outline consensus based guidelines for investigation and management. The Trent Cancer Registry was asked to undertake a retrospective baseline audit of the care of such patients in Trent.

*Results:* Soft tissue sarcomas registered with Trent Cancer Registry in 1995–1997 and meeting ICD-O criteria for site and morphology were selected. Data were collected from clinical records against a set of criteria outlined in the protocol in all registering hospitals. Data included: recording of clinical details, specialities involved in management, diagnostic and staging investigations, completeness of surgery, histological reporting, use of adjuvant therapy and outcome (death or known recurrence)

From 257 registrations, 204 cases were included. Ten percent of notes were unobtainable; 50% were initially referred to general surgeons, although definitive surgery was performed by surgeons (40%) and orthopaedic surgeons (33%); 74% saw an oncologist; 55% had a diagnostic CT or MRI scan. Only 52% had adequate staging investigations; 63% had a biopsy; but 13% were 'shelled out' at biopsy. Thirty-five percent had wide or adequate excision; in 48%, primary surgery was incomplete or marginal. Outcome was poorer in these patients. Histological reporting was variable. No consistent grading system was used; details on tumour margins were available for 66% of cases; tumour size stated in 73% of cases; 52% received adjuvant therapy; 10% being considered for EORTC trial entry. Follow-up was generally by a specialist oncology team, although 10% had no recorded follow-up.

A comparison of patients referred to a sarcoma 'expert' prior to surgical exploration with those not referred was undertaken. From the patients having surgery, the following pre-operative investigations were made: CT or MRI before definitive surgery: expert, 23/25 (92%); non-expert, 51/135 (37%); CT scan to include metastases: expert,18/25 (72%); non-expert,72/179 (40.2%); Biopsy taken: expert, 24/25 (96%); non-expert, 98/179 (55.9%); No record of tumour size in notes: expert, 7/25 (28%); non-expert, 37/179 (20.7%).
*Conclusions:* The audit demonstrates that optimal care as defined by the soft tissue sarcoma group in the Trent Region was not provided over this period within the region. All clinicians should follow management guidelines and proposals to set up specialist referral centres should be considered.

**053 Indications And Advantages Of Free Flaps In The Management Of Soft Tissues Sarcomas: A Retrospective Study Of 22 Cases**

Bonvalot S, Mamlouk K, Kolb F, Le Pøchoux C, Le Cesne A
*Department of Surgery, Institut Gustave Roussy, Villejuif, France*

*Objective:* The medical charts of patients from the same institution presenting locally advanced soft tissues sarcomas of the limbs and trunk and reconstructed with free flaps were reviewed.
*Patients and methods:* From October 1997 to March 2000, 22 patients presenting locally advanced soft tissue sarcomas were treated with large local resection and free flap reconstruction. A neoadjuvant chemotherapy was instituted in 10 cases and post-operative radiation was performed in 16 cases. The average age of patients was 46 years (SD, 17). There were 14 females and eight males. Fifteen tumors were located on the limbs and seven on the trunk. The tumor mean size was 11.5 cm with five grade I, five grade II, and 12 grade III according to the FNLCC classification. Two patients presented radio-induced sarcomas. The treatment was primary in 12 cases and 10 patients presented a recurrent tumor, two of whom had previous external beam radiation. Free tissue transfer to cover the excision site was necessary after: large skin defects secondary to direct superficial extension in 16 cases or dictated by an aberrant previous incision in two cases, recurrence after pedicled flap in two cases, filling of dead space in one case, and exposition of major vessels in irradiated areas in two cases. Major vessels and nerves resections were performed concomitantly in five cases. A total of 23 free flaps were performed with 19 latissimus dorsi and four transversal rectus abdomini musculo-cutaneous flaps. To insure the vitality of the free tissue transfer when a vital organ exposition was programmed, such as in trans-thoracic resection, four flaps were elevated in two stages according to the 'chausson aux pommes' technique advocated by Servant.
*Results:* Mean follow-up was 14 months. Margin's quality was: R0 in 17 cases (≥ 5 mm, $n = 11$; < 5 mm, $n = 6$), three of whom developed pulmonary metastasis; R1 in four cases, two of whom developed local recurrence after 5 and 6 months; and R2 in one patient who had no local control post-operatively.
Flap failure was experienced in two cases (one in a 78-year-old patient and one in a previously irradiated field; a successful second free flap was performed in this latter patient). Twenty patients (91%) had an uneventful healing that allowed undelayed adjuvant radiotherapy when indicated.
*Conclusion:* Free flap transfer is a safe and reliable technique (with a 91% success rate in our study), which enables broadening of limb salvage indications, full thickness thoracic resection and improvement of margin status.
It provides well-vascularized tissue to cover exposed vital structures and allow precocious adjuvant radiotherapy.
The use of a distant donor site does not compromise the function of an already damaged limb as would a local flap and may enhance motor results in cases of neuro-muscular reanimation.
Tissue transferred from a distant site of the excision area decreases local dissection and the potential area of local recurrence.
Performing flap transfer before tumor excision allows safe procedure in the case when vital structures exposition is programmed.

**054 Aggressive Fibromatosis. A Retrospective Study Of 72 Patients**

Sørensen A, Keller J, Nielsen OS, Jensen OM
*Centre for Bone and Soft Tissue Sarcomas, University Hospital of Aarhus, Aarhus, DK-8000, Denmark*

*Introduction:* Aggressive fibromatosis (or desmoid) has a high recurrence rate as a result of a strong infiltrative growth pattern. The purpose of this study was to evaluate outcome and influence of possible prognostic factors following surgical treatment of aggressive fibromatosis, and in an immunohistochemical project to evaluate expression of estrogen receptors.
*Materials and methods:* Seventy-two primary tumours treated in the period January 1970–September 1998 were analysed; 19 men and 53 women. Median age was 31 years (range, 1 month–77 years). Fifty patients had an extra abdominal tumour and 22 an abdominal. Median tumour size was 4 cm (range, 1–27 cm). Log-rank and Cox regression analyses were used for statistical analyses. For immunohistochemical determination, deparaffinized slides were used.
*Results:* The overall and relapse-free 5-year survival were 98 and 73%, respectively. A univariate analysis identified age, tumour size and surgical compartmentalisation as prognostic factors for local recurrence. Furthermore, radiotherapy was found to have an effect in extra abdominal tumours. A multivariate analysis identified age > 31 years, tumour size ≤4 cm and intracompartmental location as independent positive prognostic factors for local recurrence. No tumours expressed estrogen receptors.
*Conclusions:* Aggressive fibromatosis has a high local recurrence rate. Age, tumour size and surgical compartmentalisation seem to affect the local recurrence rate. The tumours do not express estrogen receptors.

**055 Adult Pelvic Sarcomas**

Dekker C[1], Houtkarnp R[1], Peterse JL[2], van Coevorden F[1]
*[1]Department of Surgical Oncology and [2] Department of Pathology, The Netherlands Cancer Institute/Antoni van Leeuwenhoek ziekenhuis, The Netherlands*

*Introduction:* Soft tissue sarcomas in the pelvis, excluding uterine sarcomas, are rare malignancies. In a retrospective study in our national cancer referral center, 33 patients (21 males and 12 females) were seen in a 25-year period. In 26 patients, either a leiomyosarcoma (18) or a rhabdomyosarcorna (8) was seen. Thirty-one out of the 33 sarcomas were intermediate or high grade.
*Recurrent disease:* Unlike soft tissue sarcomas in extremities, a high rate of local and distant recurrence (66%) was seen. In 7/22 (32%) of cases with recurrent disease, only local recurrence was seen, while in the other 15 cases (68%) recurrence presented as distant disease, synchronous with local recurrence in only one of these 15. At first presentation, nine patients already had metastatic disease. Unlike in extremity sarcoma, metastatic disease presented as pulmonary metastases in 5/22 patients (23%) only.
*Survival:* All patients with primary metastatic disease eventually died of disease, as did eight of the other 24 patients. Four of the 24 have evidence of disease, one died of unrelated causes and the remaining 11 patients are alive and well. Adults (adolescents) with rhabdomyosarcoma have a 25% NED survival versus those with leiomyosarcoma (33%). Among the others, patients with liposarcoma did best.
In this retrospective study, multimodality approach of these tumors did not appear to be more effective than single modality treatment.
*Conclusion:* Pelvic sarcomas differ from most other sarcomas in presentation and outcome. In the management of these pelvic sarcomas, these particular characteristics should be taken into consideration.

**056 Liver Resection For Metastatic Soft-tissue Sarcomas**

van Ruth S, Mutsaerts E, van Coevorden F
*Department of Surgery, Netherlands Cancer Institute / Antoni van Leeuwenhoek Hospital, Amsterdam, The Netherlands*

*Introduction:* Involvement of the liver as a metastatic site of soft-tissue sarcomas (STS) is found in less than 10% of the all STS patients. Usually it concerns visceral or retroperitoneal STS. Untreated, the median survival is about 12 months. Chemotherapy, especially for GIST tumors, shows disappointing results. In the literature, resection of STS liver metastases shows prolonged survival. Five-year survival is 10–20%. We selected the data of patients undergoing surgery in our institute for metastatic STS.
*Aims of the study:* The aims of this study are to evaluate the outcome of liver resection performed on patients with STS liver metastases and compare them with results of colorectal and other non-colorectal liver metastases. Endpoints are perioperative mortality and morbidity, post-operative hospital stay, disease free and overall survival.
*Patients and methods:* From our prospective hepatic surgery database, the files of patients with metastatic disease were selected. Pathological and surgical reports of the STS cases were analyzed. Of all patients, follow-up data were obtained. Survival was calculated according to the Kaplan–Meier method.
*Results:* Six patients with a mean age of 59 years (range, 45–65 years) underwent hepatic resection and one patient underwent repeated metastasectomy for recurrent hepatic disease 4 years later. Primary localization of the STS was the lower extremity ($n$ = 1, pleomorphic sarcoma), intestines (stomach ($n$ = 1, GIST), rectum ($n$ = 3, GIST)) and retroperitoneurn ($n$ = 1, leiomyosarcoma). The median disease free interval between primary STS and liver metastases was 48 months (range, 0–144 months). Surgical procedures included right hemiliepatectomy ($n$ = 1), right hemiliepatectomy combined with segment I resection ($n$ = 1) and five segmental resections (V/Vl, IV, IV, VII, VIII). All metastasectornies had clear margins. There was no mortality. One major complication occurred: relaparotomy was necessary because of p.o. hemorrhage. The median post-operative hospital stay was 14 days (range, 8–24 days). The median post resection survival was 32 months (range, 9–68 months). Two patients died of their malignancy (new hepatic metastases, $n$ = 2, one of them with concomitant extrahepatic metastases), four patients are still alive, two with evidence of disease (hepatic metastases, $n$ = 1; extrahepatic metastases, $n$ = 1) and two of them without evidence of disease, one only but after repeated hepatic surgery. The survival data was well comparable with the survival data for resection of colorectal metastases ($n$ = 120), but better than the survival of other noncolorectal metastases in this database ($n$ = 17).
*Conclusion:* Liver resection of metastases of soft tissue sarcomas can be performed safely and may prolong survival in patients with STS and reach at least as good overall survival figures as in patients with colorectal carcinomas.

**057 Surgery For Sarcoma Of The Spine: Outcome Analysis Of 59 Patients Over A 12-year Period**

Patrick Boland, Mark Bilsky, Murray Brennan, Robert Woodruff, John Healey
*Memorial Sloan Kettering Cancer Center, NY, NY, USA*

*Introduction:* Sarcomas may present in the spine, arising as primary tumors in the bone or neural elements, or as metastatic disease. While sarcomas are uncommon tumors relative to carcinomas, patients with these tumors more often come to surgery for spine disease because of their relative resistance to irradiation and chemotherapy. Surgery is often employed in order to preserve neurologic function, achieve spinal stability and improve local disease control. This retrospective study examines the role of surgery in patients with primary, metastatic or recurrent sarcoma of the spine.
*Methods:* Fifty-nine patients underwent 99 surgical procedures for sarcomas involving the spine between 1985 and 1997. The most common histologic diagnoses were leiomyosarcoma (14) and osteogenic sarcoma (9). Fifty-three tumors resected were high grade. The indications for surgery were: neurologic compromise (69), oncologic (25), and mechanical instability (5). The initial type of surgery included laminctomy (29), vertebrectomy (17), and spondylectomy (17). Three patients underwent enbloc vertebrectomy. Fifty-one patients underwent radiation therapy and 39 underwent chemotherapy.
*Results:* The mean survival from the time of initial diagnosis in all patients was 45 months. Greater than 90% of patients maintained the ability to ambulate and 30% of patients regained the ability to ambulate.
*Conclusions:* Spine surgery for sarcoma is valuable as a palliative measure to preserve neurologic function and improve quality of life. *En bloc* spondylectomy should be undertaken in patients with no evidence of metastatic disease in whom there is a potential for cure.

**063 The Use Of Cemented Allografts For Reconstruction Of Segmental Bone Defects After Tumour Resection**

Gerrand CH, Griffin AM, Davis AM, Wunder JS, Bell RS, Gross AE
*University Musculoskeletal Oncology Unit, Mount Sinai Hospital, Toronto, Ont., Canada M5G 1X5*

*Introduction:* Sterilization of allograft bone by irradiation may increase the risk of fracture. We routinely reinforce large-segment irradiated allograft bone with pressurized intramedullary cement. The purpose of this study was to review our experience of this technique and the outcomes and complications associated with it.
*Methods:* The prospectively collected records of all patients who underwent reconstruction of an extremity after tumour resection with a cement-reinforced allograft and potential for 3 years follow-up were reviewed.
*Results:* Thirty-nine patients met the criteria for inclusion. The diagnosis was primary bone sarcoma in 25 cases, metastatic disease in 10, soft-tissue sarcoma involving bone in two, multiple myeloma in one, and osteofibrous dysplasia in one. The allograft was intercalary in 17 cases, osteochondral in 14 and used for arthrodesis in eight cases.
Twenty-three patients were alive at a mean of 5.4 years (range, 3.0–10.1 years). Fifteen patients died at a mean of 2.3 years. One case was lost to follow-up. There were fractures of three allografts, three became infected and seven patients required secondary bone grafting for non-union. Five allografts were removed. There were two amputations for local recurrence.
*Discussion and conclusion:* Intramedullary cementing may improve the mechanical properties of irradiated bone and is associated with an acceptably low fracture rate. If cement is excluded from the graft–host junction and the junction is supplemented with autograft, the non-union rate is comparable or better than that reported elsewhere. Intramedullary cement fixation may be particularly useful with irradiated allografts since cementing likely reduces some of the harmful mechanical effects of radiation.

**068 Treatment Of Non-resectable Soft Tissue Sarcoma Of The Limbs By Isolated Limb Perfusion**

Vaglini M, Gronchi A, Pennacchioli E, Deraco M, Baratti D, Bertulli R, Casali PG, Lozza L, Rasponi A, Dileo P, Pilotti S, Azzarelli A
*Istituto Nazionale Tumori, Milan, Italy*

*Intervention:* Isolated limb perfusion (ILP) is a sophisticated technique with theoric advantages. In difficult cases, it is one of the most effective neoadjuvant treatments, able to implement conservative surgery and local control. A real impact on survival has yet to be demonstrated.
*Patients:* From August 1982 to February 2000, we observed 949 patients affected by extremity soft tissue sarcoma (girdles excluded): 68 (36 males and 32 females; mean age, 48.3 years) were judged non-resectable and were then treated by ILP. The mean follow-up was 30.5 months (range, 0–174 months). Site, histology and grade were normally represented. The ILP was carried out with antiblastic drug alone in 45 (64%) patients and antiblastic + recombinant tumor necrosis factor α (rTNFα) in 25 (36%) patients. A delayed resection of the residual tumor was performed 1–2 months after ILP.
*Results:* There was no treatment related mortality. Regional toxicity, according to the Wieberdink scale, was observed in 25 (36%) patients grade II, seven (10%) grade III, five (7%) grade IV, and three (4%) grade V. The later group underwent amputation or disarticulation. Ten (14%) patients presented nerve injury. Systemic toxicity was observed in five (7%) cases. Limb salvage was achieved in 61/70 (87%). An overall (complete + partial) immediate response was obtained in 55 (79%) patients. After resection of the residual tumor, the pathological response rates was 24 (34%) CR, 31 (44.3%) PR, six (9%) NR, one (1%) PRO and eight (11%) data not available. Six (9%) no-responder patients underwent amputation or disarticulation. In the TNF group, CR was achieved in 64%, while in the no-TNF group CR was observed in 18% ($\chi^2$, $p < 0.05$). In the TNF group, the overall response (CR + PR) was achieved in 88%, while in the no-TNF group CR + PR was obtained in 73% ($\chi^2$, $p = 0.15$). Five-year OS in the TNF and no-TNF group were 65 and 46%, respectively ($p = 0.0047$).
*Conclusions:* ILP is an effective treatment with acceptable morbidity and toxicity. The addition of rTNFα seems to improve the rate of CR and subsequent survival. Nevertheless, the high limb salvage rate, observed in our experience, is still the main determinant to define this approach as first choice treatment for non-resectable or marginally resectable soft tissue limb sarcomas.

**069 Soft Tissue Sarcomas Of The Hand And Foot**

Lin PP, Guzel VB, Pisters PWT, Zagars G.K., and Yasko AW
*University of Texas M.D. Anderson Cancer Center, 1515 Holcombe Blvd., Houston, TX 77030, USA*

*Introduction:* Soft tissue sarcomas of the hand and foot pose a special surgical challenge because of the difficulty in obtaining wide surgical margins. This study was designed to evaluate event-free outcome in a cohort of patients with hand and foot sarcomas treated with predominantly limb-sparing approaches.
*Methods:* A retrospective study was performed on 115 patients with a soft tissue sarcoma of the hand or foot between 1980 and 2000 who were evaluated, treated, and followed at this institution. The medical records were reviewed to evaluate clinicopathologic prognostic factors, treatment, and event-free outcome. Kaplan–Meier analysis was used to assess survival, and the log-rank test was used to compare different curves.
*Results:* Most patients (95%) were treated after previous, pre-referral surgery at an outside institution. Most tumors (75%) were T1 lesions (< 5 cm), and most (80%) were intermediate or high grade. Patients who were treated by wide re-excision had a 10-year local relapse-free survival of 88%, and this was significantly better than the corresponding rate of 58% for patients who did not have re-excision ($p = 0.05$). Radiation improved local control for patients who did not undergo re-excision ($p = 0.02$). However, radiation did not improve local control for patients who had wide re-excision. The overall survival rates for patients with localized disease were 76 and 65%, respectively. Survival was significantly worse for patients who had regional metastasis. Radical amputation as initial surgical treatment did not decrease the likelihood of regional metastasis and did not improve survival.
*Conclusion:* Limb sparing surgery is possible in most patients with soft tissue sarcomas of the hand and foot. Aggressive, wide re-excision is an effective method of achieving a high rate of local control. Radiation seems appropriate when margins are close or positive. There does not appear to be a survival benefit to immediate radical amputation

**070 Long-term Follow-up Of Giant Cell Tumor Of The Sacrum Treated With Selective Arterial Embolization And Other Modalities**

Lin PP, Guzel VB, Moura MF, Morello FA Jr, Benjamin RS, Gokaslan ZL, Weber KL, Yasko AW
*University of Texas MD Anderson Cancer Center Box 106, Houston, TX 77030, USA*

*Introduction:* Giant cell tumor of the sacrum is difficult to manage. Standard treatment, including surgery and radiation, are associated with significant complications and high relapse rates. The goal of this study is to evaluate the long-term outcome of arterial embolization.
*Methods:* From 1975 to 2000, 21 patients were treated for giant cell tumor of the sacrum, of which 10 had failed previous treatment. Eighteen patients underwent selective arterial embolization, of which eight had bland embolization and 10 had chemoembolization with intra-arterial cis-platin. One patient did not have embolization because of hypovascularity. Two patients underwent total sacrectomy. The mean number of embolizations was 3.8 (range, 1–7). The median follow-up time was 105 months (range, 6–205 months).
*Results:* Of the patients who underwent embolization, four failed to show a positive radiographic response (decrease in vascularity and increase in peripheral bone formation). Three additional patients had late failure of treatment. In the most favorable model, the rate of local control with embolization was 64% at 5 years and 51% at 10 years by Kaplan–Meier analysis. Patients undergoing chemoembolization with intra-arterial cis-platin did not have significantly better local control than bland embolization without chemotherapy, but this was not randomized, and in certain cases a positive effect was noted with cis-platin. Radiation provided long-term control in two patients after failure of other treatments. Three patients developed radiation sarcomas. Aggressive surgical excision was effective in obtaining local control, but functional results varied widely.
*Discussion and conclusion:* The treatment of sacral giant cell tumor continues to be problematic. Both surgery and radiation can result in significant morbidity. Arterial embolization has some efficacy, but one-half of the patients will ultimately fail treatment. Nevertheless, it is a potentially useful modality. A minority of patients can have their disease controlled by embolization alone. Many patients show a partial response and this may make surgical excision safer and less morbid. A multimodality approach may provide optimal results.

**071 Survivorship Of Segmental Prosthetic Arthroplasty For Limb Salvage Following Bone Sarcoma Resections**

Yasko AW, Lin PP, Weber KL
*The University of Texas M. D. Anderson Cancer Center, Houston, TX 77030, USA*

*Introduction:* Prosthetic arthroplasty is the most common method of reconstruction of segmental bone defects following resection of bone sarcomas. The purpose of this study was to determine the survivorship of the reconstructions in short- and long-term follow-up.

*Methods:* A retrospective study was performed on all patients diagnosed with a bone sarcoma between 1980 and 1995 who were treated with a limb-sparing osteoarticular resection and prosthetic arthroplasty reconstruction. Prosthetic survival was calculated with endpoints of analysis based on any event, any prosthesis-related event and aseptic loosening of the prosthesis, which led to prosthetic revision, removal or limb amputation.
*Results:* A total of 237 reconstructions were performed involving the distal femur ($n$ = 111), proximal tibia ($n$ = 43), proximal humerus ($n$ = 47), and proximal femur ($n$ = 36). All implants were fixed with polymethylmethacrylate cement. A total 174/237 (73%) did not require a re-operation at last follow-up evaluation. Early complications (within 1 year post-operatively) developed in fewer than 2% of patients. Aseptic loosening occurred late and accounted for the majority of events resulting in prosthetic failures (51%). Amputations were performed in 10% of patients prosthesis-related complications or tumor recurrence.
Prosthetic Arthroplasty Survival (prosthetic-related events)

| | 5 year | 10 year | 15 year |
|---|---|---|---|
| Distal femur | 83 | 63 | 60 |
| Proximal tibia | 90 | 56 | 56 |
| Proximal femur | 91 | 76 | 76 |
| Proximal humerus | 94 | 87 | 87 |

*Conclusion:* The early outcome of prosthetic arthroplasty was extremely favorable, supporting this method of reconstruction following excision of high-grade bone sarcoma. Long-term survival of prosthetic arthroplasty can be anticipated following tumor resection about the shoulder and hip. Aseptic loosening continues to be the primary cause of prosthetic failure, especially about the knee.

**072 Pathologic Fractures In Osteosarcoma**

Saghieh SS, Lin PP, Weber KW, Jaffe N, Patel SR, Benjamin RS, Yasko AW
*The University of Texas M. D. Anderson Cancer Center, Houston, TX 77030, USA*

*Introduction:* The majority of patients with osteosarcoma can be treated with limb salvage surgery. However, those who present with a pathologic fracture may be at a higher risk for local recurrence and poorer survival.
*Method:* This is a retrospective study of 65 patients diagnosed with osteosarcoma associated with a pathologic fracture treated between 1981 and 1998. Medical records, pathology reports and radiologic studies were reviewed in all of these patients, and prognostic variables evaluated for local recurrence and overall survival.
*Results:* There were 37 males and 28 females. Twenty-seven were younger than 17 years, and 13 were older than 45 years. Fifty-two patients presented with localized and 13 with metastatic disease. The distal femur was the most frequent site (40%) of involvement. Limb salvage surgery was performed for 34 patients (52%). Amputation was performed for 31 (48%). The overall survival for patients with localized disease was 62% at 5 years. From all variables studied for patient survival, only localized disease (versus metastatic disease) and favorable chemotherapy response (versus poor response) were positive prognostic factors. Twelve (18%) local recurrences developed, nine (26%) after limb salvage and three (9%) after amputation. Of the prognostic variables analyzed for local recurrence, none reached statistical significance. However, limb salvage surgery for patients with localized disease who had a prior non-oncologic surgery resulted in a high percentage of local failures (4/5 patients (80%)).
*Conclusion:* Overall survival in patients with osteosarcoma who sustain a pathologic fracture is not compromised; however, local control can be jeopardized especially in patients who undergo extensive non-oncologic surgery prior to diagnosis and who subsequently are treated with limb salvage surgery. Although local control of the tumor must be individualized, amputation may be the surgery of choice for this high-risk group.

**080 Combined Surgery, Brachytherapy And External Irradiation Of Locally Advanced Soft Tissue Sarcomas: A Feasible Approach**

Pedersen JG[1], Krarup-Hansen A[2], Daugaard S[3], Höjlund B[4], Rosendal F[2], Larsen jS[2], Lund B[1], Engelholm SA[2]
*[1]Department of Orthopaedic Surgery, [2]Department of Radiation Oncology, [3]Department of Pathology, and [4]Department of Radiology, National University Hospital (Rigshospitalet), DK-2 100 Copenhagen, Denmark*

*Background:* Locally advanced disease of soft tissue sarcomas (STS) is a major surgical task: maximum local control versus minimum surgery and disability.
*Aim:* To reduce the amount of extensive surgery, to improve local control by immediate use of post-operative turnour (T) bed brachytherapy (brt) followed by external beam irradiation (RT), and to reduce morbidity.
*Materials and methods:* Sixteen patients (pts) (median age, 60 years; range, 29–75 years; female/male, 9/7) with a blank chest X-ray had an MR/CT scan and a diagnostic biopsy. Fifteen pts had a primary T larger than 5 cm, and one less than 5 cm; nine were located at the lower and four at the upper extremity, and three superficial at the thorax; two had a grade (gr) I T, 10 a grade II and three a grade III; and eight had liposarcoma, three myxofibrosarcoma, two leiomyosarcoma, one myogenic sarcoma, one synovial sarcoma, and one malignant haemangiopericytoma. All pts underwent surgical procedure (10 intralesional excision, four marginal, two wide) and intraopererative implantation of microSelectron (mSr) flexible applicators (median, 6; range, 3–18), each tube with a maximal loading length of 23 cm. The following day, the tubes were connected to the PDR-mSr treatment unit and a mean dose of 22 Gy (range, 20–30 Gy) was applied at a dose rate of 0.6 Gy/hour; one pulse per hour with a CT scan calculated 100% at reference points 5 mm from the catheters. The tubes were extracted immediately after finishing the brt. The volume implants were reconstructed from orthogonal radiographs and the 3D-dose distribution calculated in CADPLAN using a geometric optimisation algorithm for stepping source implants. The mean treatment volume was 104 ml (range, 45–200 ml). Conventional fractionated compartmental external beam RT was initiated at day 22 (range, 9–34 days) and a total cumulated mean dose of 49 Gy (range, 44–50 Gy) in 2-Gy fractions was given.
*Results:* Fifteen pts are alive, 14 with no sign of local recurrence at a median follow-up of 16 months (range, 12–49 months); one pt died of lung metastases at 27 months; one pt with intralesional excision had multi-focal local recurrence of dedifferentiated liposarcoma (grade II > grade III); one pt is alive with lung metastases. One pt (brt, 30 Gy; external RT, 50 Gy) had quite a severe RT-induced damage and needed skin-transplantation, after which the pt recovered. Brt was reduced to 20 Gy and no further events of RT damage were observed. Overall, only minimal disability was observed.
*Conclusion:* Combined modality treatment of locally advanced STS is a safe and feasible approach. This early report needs further confirmation in a long-time observation large-scale study.

**065 Combined Therapy For Retroperitoneal Soft Tissue Sarcomas: Pre-operative Radiation And Surgery**

Gronchi A, Azzarelli A, Baratti D, Lombardi F, Gandola L, Navarria P, Bertulli R, Casali PG, Pennacchioli E, Rasponi A, Dileo P, Pilotti S
*Istituto Nazionale Turnori, Milan, Italy*

*Intervention:* Surgery is the standard treatment of retroperitoneal soft tissue sarcomas (RSTS), but is affected by a high failure rate.

Adjuvant radiation may improve local control and possibly survival. We report the results of a phase I–II study on radiation therapy (RT) delivered in a pre-operative setting, to determine the treatment feasibility and its possible benefit on surgical quality.
*Patients and methods:* From September 1996 to December 1999, 41 patients affected by RSTS were referred to our institution. Twenty-one of them, affected by primary or recurrent disease, were eligible for the combined therapy. As regards to the histology, 14 were liposarcoma, five were leiomiosarcoma, one rhabdomiosarcoma and one abdominal gastro-intestinal stromal tumor. Seventeen were low grade and four high grade. A CT-based computerized treatment plan was obtained in all patients to determine treatment volume. Personalized shields were realized to confirm dose distribution. The scheduled dose was 50 Gy. Surgery was then planned between the 30th and the 60th day after the end of RT.
*Results:* One patient received only 32 Gy for gastro-intestinal intolerance. All the other patients completed the treatment without significant toxicity. One patient progressed soon after RT and was not operated. All the other 20 patients underwent surgery 32–46 days after the end of RT. A complete removal of the tumor was possible in 15 cases: resection was extended to viscera with 10 nephrectomies, seven colectomies, two distal pancreatectomies, one splenectomy, one gastric resection and one resection of the inferior vena cava. No major surgical difficulties were encountered. In the remaining five cases not completely resected, a sarcomatosis was found at laparotomy. One patient died 3 months later due to related abdominal complications. One patient died 12 months later due to local abdominal recurrence, and two died 22 and 32 months later, respectively, due to distant metastasis. The remaining 11 patients are all alive with a median follow-up of 25 months (range, 6–40 months), eight of whom free of disease.
*Conclusions:* Pre-operative radiation is feasible and safe. A controlled study with strict inclusion criteria is now recommended to investigate the possible benefit of the combined treatment on survival.

**066 Chordoma: Natural History And Treatment Results In 55 Patients**

Gronchi A, Azzarelli A, Baratti D, Lozza L, Bertulli R, Casali PG, Pennacchioli E, Rasponi A, Dileo P, Pilotti S
*Istituto Nazionale Tumori, Milan, Italy*

*Intervention:* Chordoma is a rare neoplasm with poor prognosis as already observed by several authors. Our experience reports one of the largest series in the literature and confirms the need of new adjuvant treatments.
*Patients:* We observed at our institution 55 patients affected by chordoma. The neoplasm was located in the sacrum in 49 cases, at the base of the skull in three and in the vertebral bodies in three. The median age at the time of the diagnosis was 58 years (range, 2–77 years); 34 patients were male and 21 female; median diameter was 13 cm (range, 2–30 cm). During the 1970s, advances in pre-operative staging, more reliable surgical techniques and a clear definition of oncological adequacy were the basis of a new rationale treatment of sacral chordoma. Since 1977, patients underwent surgery with intent of radicality. The technique of operation performed in 29 patients was high amputation of the sacrum by posterior approach, with the aim to perform an uncontaminated removal of the entire lesion and surrounding normal tissue. Radiotherapy was considered in case of marginal or intralesional operations.
*Results:* Surgical margins were rated as wide in nine patients, marginal in 17 and macroscopically intralesional in three. Post-operative radiation therapy was performed in nine patients with marginal/intralesional surgery; one patient underwent pre-operative RT. Sacral chordoma recurred in 17 out of these 29 recent operated patients. Local failure, mainly located in the ischiorectal area, developed in 13 cases, distant isolated metastases (lung) in one patient, and both local and distant failure in three, with involvement of lung in two cases and distant subcutaneous dissemination in one. As regard to resection margins definition, local ± distant failure developed in six out of nine (66.7%) patients with wide margins and in 11 out of 17 (64.7%) patients with marginal operation.
*Conclusions:* Although chordoma is considered a low-grade lesion, surgical treatment to obtain effective local control is difficult, due to late diagnosis and anatomical site. In the present study, wide surgical margins were obtained only in 31% of the operated patients and recurrence rate was high. Distant metastases, isolated or concurrent to local failure, were detected only in four out of 29 patients (13.7%). Therefore, strong effort in achieving complete surgical excision is mandatory, as well as in developing more effective adjuvant therapies.

**087 Periosteal Chondrosarcoma: Experience Of The Istituto Ortopedico Rizzoli.**

Fabbri N, De Paolis M, Vanel D, Mercuri M, Picci P
*Department of Musculoskeletal Oncology, Istituto Ortopedico Rizzoli – Bologna, Italy*

Periosteal chondrosarcoma is a rare primary malignant tumor of the bone surface. Due to its rarity, the differential diagnosis with other primary tumor of the bone surface can be difficult and the potential for metastasis remains unclear. Studies reporting series of patients homogeneously managed at the same institution are scant in the literature. From 24,855 bone tumors filed at our institution, 26 cases of periosteal chondrosarcoma were identified; two cases were excluded for incomplete imaging studies. A retrospective study of the remaining 24 cases was undertaken. Clinico-pathologic features were reviewed and analyzed along with follow-up in order to define diagnostic criteria and understand prognosis of this condition.
There were 18 males and six females, and average age was 33 years (range, 17–65 years). A palpable mass and dull pain averaging more than 18 months of duration were the most common symptoms at presentation.
Tumor location was the femur in 14 cases (12 distal, two proximal), humerus in seven cases (five proximal, two shaft), tibia in two cases (two proximal) and iliac wing in one case. Imaging studies showed a frequently lobulated juxtacortical mass, usually suggestive of cartilaginous matrix on both plain X-rays and CT or MRI scan. Although abnormalities of the cortex were present in all the cases, medullary involvement could be detected and histologically confirmed only in two cases.
Information regarding the surgical margin and a minimum follow-up longer than 3 years were available in 18 patients (average, 11 years; range 3–30 years). Surgical margin was adequate (wide) in 10 cases and inadequate (wide/contaminated or marginal) in the remaining eight cases. All the patients were disease free; three local recurrences were managed with further surgery. None of the patients developed metastasis. There were no local recurrences when the surgical margin was adequate. In our experience, prognosis is excellent after adequate surgical management and risk of metastasis is very low.

**088 Desmoplastic Fibroma: Experience Of The Istituto Ortopedico Rizzoli**

Fabbri N, Trentani F, Vanel D, Mercuri M, Picci P, Bertoni F
*Department of Musculoskeletal Oncology, Istituto Ortopedico Rizzoli – Bologna, Italy*

Desmoplastic fibroma is a rare benign tumor of bone. Despite its benign nature, a marked tendency for local recurrence has been reported in the past. However, studies reporting series of patients homogeneously managed at the same institution are scant in the recent literature.
From 24,855 bone tumors filed at our institution, 16 cases of desmoplastic fibroma were identified. A retrospective study of this group of patients was undertaken; clinico-pathologic features were reviewed and analyzed along with follow-up in order to define biologic aggressiveness and potential for local recurrence of this disorder.
There were nine males and seven females, and average age was 22 years (range, 9–36 years). Swelling and pain usually lasting more than 6 months were the most common presenting symptoms. Tumor location was the femur in five cases (three proximal, two distal), tibia in three cases (two proximal, one distal), humerus in two cases (one proximal, one shaft), fibula in two cases (one proximal, one distal), and calcaneus, periacetabular region, sacrum and L2 body in one case each.
Imaging studies constantly showed a purely lytic defect, sharply marginated towards the bone and contained by paper thin cortex towards the surrounding soft tissues. Staging of the tumor according to MSTS was stage 2 in 12 cases, stage 3 in three cases, and stage 1 in one case.
Information regarding the surgical margin and a minimum follow-up longer than 3 years were available in 14 patients (average, 5 years; range, 3–22 years). Surgical management consisted of wide resection in nine cases and intralesional curettage in five cases; in three cases, the use of phenol or liquid nitrogen was added to the curettage as a local adjuvant.
All the patients were disease free. None of the patients developed metastasis. In our experience, prognosis is excellent after adequate surgical management, either wide resection or aggressive curettage.

**089 Hemangiopericytoma Of Bone: The Rizzoli Experience**

Campanacci L, Fabbri N, Vanel D, Mercuri M
*Department of Musculo Skeletal Oncology, Istituto Ortopedico Rizzoli, Bologna, Italy*

Hemangiopericytoma is a rare malignant vascular tumor, originating from the perivascular cells. Usually arising in the soft tissues, it is extremely rare in the skeleton. We coud not find large series reports in the literature.
Treatment is surgery, sometimes associated with radiation and/or chemotherapy. At the Rizzoli Institute, since September 1900 to date, 24,855 cases of bone and soft tissue tumors and pseudotumoral lesions are filed. Out of 6434 cases of malignant bone tumors (excluding metastases), only 13 cases of hemangiopericytoma primary of the bone were observed (two of which were consultations).
There were four males and nine females, aged 8–79 years (average, 41 years). Pelvic bones and/or lumbo-sacral spine were involved in six cases, a rib in one, upper and lower limbs in three cases each (femur in two, fibula in one, and humerus in three). In two cases, the lesion involved two adjacent bones.
Symptoms were always present since a long period of time (from 6 to 60 months; average, 25.5 months). Radiologically, all cases presented as purely ostelitic, containing trabecular septae, with expanded cortical bone; in 10 cases, a soft tissue mass was also present. In two cases, small osteolitic images distal to the primary lesion were observed; two cases presented with a pathological fracture.
Among the 11 cases treated at our institution, five were managed with surgery alone, one with surgery and radiation-therapy; three patients were treated by chemotherapy and radiation therapy (surgery was not possible at presentation). One case (8-year-old girl) was cured with chemotherapy alone.
Six patients died of the disease (all developed lung metastases, one a local recurrence) after 5 months to 6 years; five are continuous disease free at 4–21 years of follow-up.
Hemangiopericytoma still represent a difficult issue for diagnosis and treatment, its course can be rapidly aggressive, and lung metastases can appear even after years; the role of chemotherapy is still uncertain in improving the outcome. Delaied diagnosis has an ominous prognosis.

**090 Soft-tissue Sarcoma (Sts) — Adherence To Guidelines**

Nijhuis PHA[1], Schaapveld M[2], Otter R[2], Hoekstra HJ[1]
*[1]Department of Surgical Oncology, Groningen University Hospital, Groningen, The Netherlands, and [2]Comprehensive Cancer Center North-Netherlands, Groningen, The Netherlands*

*Introduction:* Because many general surgeons are unfamiliar with these rare tumors, a reasonable number is found by chance, often after inadequate resection, leading to complex definitive treatment. Therefore, guidelines for diagnosis and treatment were developed. As the diagnostic management is essential for definitive treatment, adherence to these guidelines is important.
*Methods:* Primary STS, registered by the Comprehensive Cancer Center North-Netherlands from January 1989–January 1996, were analyzed retrospectively with regard to adherence to the diagnostic guidelines. Urogenital STS, gastro-intestinal STS, and Kaposi's sarcomas were excluded.
*Results:* Three hundred and fifty-one STS were analyzed. In the specialized center, 69% of patients were younger than 60 years, whereas in district hospitals 63% were older than 60 years. With increase of age, referral to the center declined in a linear fashion. For all guidelines, adherence was significantly better in the center (Table 1).
*Conclusions:* In many aspects of the diagnostic process of STS, existing guidelines were not followed, especially in community hospitals. Adherence to all individual guidelines was significantly better in the specialized center. In order to improve compliance with future STS guidelines, appropriate guideline development, dissemination, and implementation programs should be developed. Concentration of STS in a limited number of hospitals and intensified collaboration with specialized centers seem advisable. Special attention should be paid to older patients, which were significantly more often not referred to a specialized center.

**091 Pre-operative Radiation Therapy In The Treatment Of Soft Tissue Sarcomas**

Virkus, Mollabashy A, Reith JD, Berrey HB, Zlotecki RA, Scarborough MT
*University of Florida, Gainesville, FL 32610, USA*

This report presents the results of a cohort of patients with a soft tissue sarcoma treated with a standard protocol including pre-operative radiation therapy (PRT) and surgical resection, assessing wound complications, local recurrence, and oncologic outcome.
A standard protocol of PRT followed by *en bloc* resection was performed in 218 patients with a primary soft tissue sarcoma from 1984 to 1999. Wound complications were defined as minor (local wound care), moderate (operative I&D), major (STSG or flap), and amputation. Wound complications are reported for the entire patient population. Oncologic results are reported on a subset of patients that excluded patients who were stage III at the time of resection, or received chemotherapy. There were 114 males, 104 females, with an average age of 56 years (range, 8–86 years). Thirty-six patients received chemotherapy. At the time of resection, 13 patients were stage IA, 16 were stage IB, 63 were stage IIA, 111 were stage IIB, and 15 were stage III. Ten patients had an intralesional surgical margin, 91 marginal, 115 wide, and two radical. Primary wound closure was obtained in 187 patients, nine needed a split-thickness skin graft, 20 a rotational flap, and two a free flap for closure. Only six patients underwent primary amputation. Mean oncologic follow-up was 53 months.
The overall wound complication rate was 32.6% (minor, 10%; moderate, 13%; major, 7%; amputation, 1%). If minor wound problems treatable with short-term local wound care are excluded, the wound complication rate was 22.9%. When stage III and chemotherapy patients were removed, 172 patients remained for oncologic outcome analysis. Seventy-three (42%) developed late metastases, predominantly to the lungs (82%). Eighteen patients developed a local recurrence, which was 15.1% of those patients with a minimum of 2 years of follow-up. The recurrence rate in patients with prior surgery at an outside institution was 24%, versus 12% if initial surgery was at our institution. At latest follow-up, 80 patients (46%) were continuously disease free, six (3%) ANED, eight (4%) alive with disease, and 65 (38%) dead of disease. The 2 and 5-year survival rates were 77 and 66%, respectively.

The ideal timing of adjuvant radiation therapy remains to be determined. PRT allows for a smaller radiation dose and field to be delivered, theoretically decreasing the morbidity of radiation. Our local control rate is similar to that seen in other studies where a high percentage of adequate surgical margins is obtained. Twenty-two percent of our patients required a return to the operating room for wound complications. This is similar to the wound complication rates in studies where post-operative XRT or no XRT is used. Distant control continues to be a difficult problem in the treatment in soft tissue sarcomas.

**095 Outcome Following Local Recurrence In Osteosarcoma**

Grimer RJ

*The Royal Orthopaedic Hospital Oncology Service, Bristol Road South, Birmingham, UK*

*Purpose:* Local recurrence of osteosarcoma is generally thought to be a death sentence, with few patients surviving. This paper addresses this question and attempts to identify possible prognostic factors and treatment options for patients with local recurrence.

*Method:* All patients who had developed a local recurrence following treatment of a non-metastatic high-grade osteosarcoma were included in this review. Patients with metastases at the time of diagnosis were excluded. Eighty-one patients were found to fulfill the above criteria. LR arose at an average of 16 months (range, 3–49 months). Sixteen of the patients were already known to have lung metastases and a further 27 were found to have metastatses within 3 months of treatment of the LR. Another 25 developed metastases later, leaving only eight patients who did NOT subsequently develop metastases.

*Results:* Overall survival was most clearly related to whether or not metastases were present at the time of presentation with LR. In patients with known metastases or ones that appeared within 3 months, there were no survivors past 30 months. For those with no metastases within 3 months, the survival was 40% at 5 years from the time of LR. Even in those 25 who developed metastases later, the survival was 30% at 3 years and 23% at 5 years. Disease free interval was not significant.

*Conclusion:* LR accompanied by metastases is currently a death sentence. Experimental treatments for these patients may be justified. In patients with solitary LR, aggressive treatment is justified and has a similar outlook to that for lung metastases alone.

**107 Combined Surgery, Brachytherapy And External Irradiation Of Locally Advanced Soft Tissue Sarcomas: A Feasible Approach**

Pedersen JG[1], Krarup-Hansen A[2], Daugaard S[3], Hojlund B[4], Rosendal F[1], Larsen JS[2], Lund B[1], Engelholm SA[2]

*Departments of [1]Orthopaedic Surgery, [2]Radiation Oncology, [3]Pathology, and [4]Radiology, National University Hospital (Rigshospitalet), DK-2100 Copenhagen, Denmark*

*Background:* Locally advanced disease of soft tissue sarcomas (STS) is a major surgical task: maximum local control versus minimum surgery and disability.

*Aim:* To reduce the amount of extensive surgery, to improve local control by immediate use of post-operative turnour (T) bed brachytherapy (brt) followed by external beam irradiation (RT), and to reduce morbidity.

*Materials and methods:* Sixteen patients (pts) (median age, 60 years; range, 29–75 years; female/male, 9/7) with a blank chest X-ray had an MR/CT scan and a diagnostic biopsy. Fifteen pts had a primary T larger than 5 cm, and one less than 5 cm; nine were located at the lower and four at the upper extremity, and three superficial at the thorax; two had a grade (gr) I T, 10 a grade II and three a grade III; and eight had liposarcoma, three myxofibrosarcoma, two leiomyosarcoma, one myogenic sarcoma, one synovial sarcoma, and one malignant haemangiopericytoma. All pts underwent surgical procedure (10 intralesional excision, four marginal, two wide) and intraopererative implantation of microSelectron (mSr) flexible applicators (median, 6; range, 3–18), each tube with a maximal loading length of 23 cm. The following day, the tubes were connected to the PDR-mSr treatment unit and a mean dose of 22 Gy (range, 20–30 Gy) was applied at a dose rate of 0.6 Gy/hour; one pulse per hour with a CT scan calculated 100% at reference points 5 mm from the catheters. The tubes were extracted immediately after finishing the brt. The volume implants were reconstructed from orthogonal radiographs and the 3D-dose distribution calculated in CADPLAN using a geometric optimisation algorithm for stepping source implants. The mean treatment volume was 104 ml (range, 45–200 ml). Conventional fractionated compartmental external beam RT was initiated at day 22 (range, 9–34 days) and a total cumulated mean dose of 49 Gy (range, 44–50 Gy) in 2-Gy fractions was given.

*Results:* Fifteen pts are alive, 14 with no sign of local recurrence at a median follow-up of 16 months (range, 12–49 months); one pt died of lung metastases at 27 months; one pt with intralesional excision had multi-focal local recurrence of dedifferentiated liposarcoma (grade II > grade III); one pt is alive with lung metastases. One pt (brt, 30 Gy; external RT, 50 Gy) had quite a severe RT-induced damage and needed skin-transplantation, after which the pt recovered. Brt was reduced to 20 Gy and no further events of RT damage were observed. Overall, only minimal disability was observed.

*Conclusion:* Combined modality treatment of locally advanced STS is a safe and feasible approach. This early report needs further confirmation in a long-time observation large-scale study.

**109 Unplanned Excision For Soft Tissue Sarcoma: Identification Of A Subgroup With Bad Prognosis**

van Geel AN, Eggermont AMM, Schmitz PIM

*Department of Surgical Oncology and Statistics, University Hospital Rotterdam/Daniel den Hoed Cancer Center, Rotterdam, The Netherlands*

*Introduction:* Unplanned excision (UE) for soft tissue sarcoma (STS) is a frequent procedure. Unclear is the influence on prognosis.

*Material and methods:* From 1986 to 1993, 86 patients (32.7% of all referred patients) were treated after UE of STS elsewhere (subfascial; limbs, girdles and trunk, > 5 CMS). All patients had CT or MRI before definitive treatment; minimal follow-up was 5 years.

*Results:* Thirty-one patients (36.1%) had complications (hematoma) after UE. In 46 patients (53.5%), residual tumor was seen on CT/MRI and found at pathological examination in 66 patients (76.7%). In one-half of the patients, definitive treatment was considered to be more complex, resulting in increased morbidity.

*Conclusion:* After multivariate analysis, a group of patients (35 patients = 40%) with a significant bad prognosis was found: older than 60 years with a second treatment with increased complexity and morbidity independent of this treatment is radical or not. This

| Survival | Overall | Loc. rec. free | Dist. rec. free |
|---|---|---|---|
| 2 years | 86.0 | 85.8 | 84.6 |
| 5 years | 74.3 | 72.1 | 70.6 |

group can be considered as a high-risk group eligible for adjuvant chemo therapy trials.

### Posters — Radiation Oncology

### 029 Potential Impact Of High Technology Radiation Treatment For Sarcomas To Improve Patient Outcome

Herman Suit, Karen Doppke, Jong Kung, J. Michael Collier, Ira Spiro, Thomas Delaney
*Department of Radiation Oncology, Massachusetts General Hospital, Harvard Medical School, Boston, MA, USA*

The available radiation treatment techniques employed in the management of the mesenchymal tumors cause substantial quantities of non-involved tissues/structures to receive important dose levels. The consequence is that there is a non-negligible frequency of treatment associated morbidity. This is in the form of: excess fibrosis, wound healing delay or breakdown, late appearing damage in major nerves and vessels, pathological fracture and the rare radiation induced malignant neoplasm. There are several new techniques, which will achieve full coverage of the defined target tissue for each treatment session, but with major reductions in the volume of normal tissues/structures included in the high dose volume. As complications of treatment cannot develop in unirradiated tissues, there is predicted a major lowering of the frequency and severity of treatment related morbidity. These new techniques are principally intensity modulated X-ray therapy, intensity modulated proton beam therapy, on-line diagnostic quality imaging, Monte Carlo based dose calculations, etc. The presentation will assess the impact of these developments on three relatively common clinical problems. These are lesions located in the: (1) medial proximal thigh, (2) thoracic vertebral body, and (3) retroperitoneal region.

### 031 Effects Of Irradiation And Radio-protectant On Rat Growth Plate Morphology

Damron TA, Spadaro JA, Farnum CE, Margulies BS, Strauss J
*Upstate Medical University Department of Orthopedic Surgery, Syracuse, NY 13224, USA*

*Introduction:* Previous work in our laboratory has shown a beneficial effect on rat limb growth from administration of the radioprotectant amifostine. The histological correlates of these quantitative measurements of limb length have not previously been described.
*Purpose:* The purpose of this paper is to detail the histological changes over time in the rat growth plate (GP) following irradiation with and without the radioprotectant amifostine.
*Methods:* Histological specimens were examined from 84 Sprague–Dawley male rats, 4 weeks old, previously included in reports detailing limb length effects. Groups of $n = 6$ included for analysis were 'cage controls' at 6 weeks, irradiation only; (single dose, 12.5 and 17.5 Gy) at 6 weeks, 'radioprotectant pretreatment' (100 and 300 mg/kg amifostine for the 12.5 Gy groups, and 100 mg/kg for the 17.5 Gy groups) at 6 weeks, and 'early timing' (0.5, 1, 2, 3, and 4 weeks post 17.5 Gy with and without 100 mg/kg amifostine). Irradiation was administered to the right distal femur and proximal tibia; the left leg served as the control.
*Results:* Morphology of the irradiated GP changed consistently over time. At 0.5 weeks, a subtle decrease in cellular profiles with maintenance of overall zonal architecture was evident. At 1 week, cellular profiles were notably reduced, and zonal architecture was nearly lost. At 2 weeks, the GP was composed of sparsely situated terminal, hypertrophic-appearing chondrocytes. At 3 weeks, cellular profiles increased via clonal bodies. At 4 and 6 weeks, clonal bodies predominated.
The growth plate cellular profile area following irradiation hit a nadir at 2 weeks and rebounded at the third week toward normal. TRAP staining showed an increase in osteoclasts between the 2 and 3 week time periods that corresponded to an relative decrease in GP height over the same interval.
Epifluorescent growth rates reached a nadir 1 week following irradiation and slowly returned toward normal thereafter.
Growth plate overall height was greater for controls than irradiated specimens at all time periods, but these effects were much greater centrally than peripherally. Amifostine effects, however, were observed more consistently at the periphery rather than in the central GP.
*Discussion:* Irradiation results in a predictable pattern of histologic changes, with the greatest effects observed at 1 week in the rat proximal tibia. Prereatment with amifostine is unable to preserve normal GP morphology despite its maintenance of longitudinal growth, but many parameters are statistically improved compared with the irradiated specimens.

### 032 Bone Density Effects Of Radiation And The Radioprotectant Drug Amifostine In A Rat Model

Morgan H, Damron TA, Margulies B, Spadaro J
*Upstate Medical University Department of Orthopaedic Surgery, Syracuse, NY 13224, USA*

*Purpose:* The purpose of this project was to examine the effects on the bone density measurements of irradiated rat femurs with and without the radioprotective drug amifostine (AMF).
*Methods:* Seventy-two weanling Spague–Dawley rats were randomized into treatment groups. In all treated animals, the right knee (distal femur and proximal tibia) was irradiated with single-fraction 17.5 Gy, while the left leg was used as an internal control. Animals were sacrificed at 0.5, 1, 2, 3, 4, and 6 weeks following irradiation, 12 per time period. One-half of the animals in each of the groups received 100 mg/kg amifostine 20 minutes prior to irradiation. For the bone density analysis, the skeletonized femurs were placed into a cylindrical holder and suspended in deionized water. Bone density (g/cm) was determined using peripheral quantitative computed tomography (pQCT; Norland XCT 2000, Fort Atkinson, WI), resolution 90 microns.
*Results:* The irradiated slices maintained a significantly higher BMD than controls through 6 weeks in the most proximal metaphysis and through 3 weeks in the distal metaphysis (Fig. 1). The slice closest to the metaphysical side of the physis demonstrated a unique early peak BMD at 2 weeks, which decreased dramatically by 3 weeks. The BMD for rats that received amifostine was significantly lower through 3 weeks than for rats that received radiation alone ($p < 0.02$ for 0.5, 2, and 3 weeks).
*Discussion:* Three conclusions are drawn from the current data. First, at early time periods following irradiation, BMD within the irradiation field is greater than control in this animal model Second, a 2-week early peak in BMD occurs in the juxta-physeal metaphysis. Third, amifostine has a significant effect in holding BMD close to normal in the juxta-physeal region. We hypothesize that the sensitive marrow elements, particularly in this case the monocyte precursor of osteoclasts, are more sensitive to irradiation damage than the osteoblasts, given the slower turnover of the latter cells. In addition, we hypothesize that the early 2-week peak in BMD occurs only in the distal metaphysis due to the greater relative contribution of provisional calcification to BMD there, since this calcification is a passive process that is likely to continue despite radiation effects, particularly when unchecked by resorptive activity of chondroclasts and osteoclasts. These hypotheses are supported by histological observations.

**033 Pattern Of Local Recurrence After Conservative Surgery And Radiotherapy For Soft Tissue Sarcoma.**

Cleator SJ, Cottrill C, Harmer CL
*Sarcoma Unit, Royal Marsden Hospital NHS Trust, London SW3 6JJ, UK*

*Purpose:* Over the past two decades, our centre has adopted a policy of conservative surgery followed by adjuvant radical-dose radiotherapy for all medium- and high-grade soft tissue sarcomas. From the patients treated in this manner at the Royal Marsden Hospital over the past 15 years, we analyzed 17 who had relapsed locally. We discuss technical factors that may have contributed to local relapse in some cases, and the geographical relationship between sites of recurrence and the phase 1 and 2 radiotherapy volumes.
*Patients and methods:* We examined those cases recorded on our soft tissue sarcoma database between January 1986 and September 1999 who had recurred locally following surgery and radical post-operative radiotherapy. We excluded patients with residual macroscopic disease following surgery and any patients with metastatic disease at presentation. We recorded details relating to original tumour stage, histological type, grade and surgical margins. The cohort included eight patients with T1 (47%) and nine patients with T2 tumours (53%). The tumours were grade 1, 2 and 3 in two (12%), six (35 %) and nine (53%) number of cases, respectively. The majority of patients were treated with a phase I volume corresponding to the entire muscle compartment that received 50 Gy in 25 fractions over 5 weeks. The phase II was a reduced volume corresponding to the tumour bed and received 10 Gy in five fractions during the sixth week of treatment. Four of the patients were treated according to a hyperfractionated regimen consisting of 72 Gy in 60 fractions twice daily over 6 weeks with volume reduction after 60 Gy.
*Results:* Mean time to local recurrence was 26 months. Four (23%) patients recurred within the phase I volume, nine (53%) recurred within the phase II volume, and three (18%) outside the irradiated volume. One recurrence was marginal. Eight patients who relapsed had positive surgical excision margins originally. In six patients, there had been deviations from our radiotherapy protocol, usually unavoidable, which may have accounted for treatment failure; these included all three out of field recurrences. Of the 11 patients for whom it was possible to adhere strictly to protocol, two recurred in the phase I volume and nine in the phase II volume. With a mean follow-up time since completion of radiotherapy of 63 months, seven patients have died and, of these, six were disease related.
*Conclusion:* A dose greater than 60Gy may be necessary despite initial complete surgical resection. Prospective, multi-centre data collection and ideally a randomized trial are required to formulate an improved treatment policy.

**036 Treatment Outcome In Patients Radiated For Spinal And Paraspinal Tumors**

DeLaney TF, Ancukiewicz M, Hornicek FJ, Spiro IJ, Harmon DC, Suit HD
*Massachusetts General Hospital, Boston, MA 02114, USA*

*Objective:* Tumors of the spine and paraspinal soft tissues can present difficult management challenges because of the proximity of the spinal cord, which limits resection margins and radiation dosage. A retrospective review of treatment outcome in a cohort of these patients managed in a single institution was undertaken to assess prognostic factors and treatment techniques important for favorable treatment outcome.
*Materials and methods:* Fifty-seven patients with spinal and paraspinal tumors treated with radiotherapy between 1971 and 1998 were identified in our sarcoma database. Nine patients with Ewing's sarcoma/PNET were identified for separate analysis, leaving 48 patients for analysis for this study. Outcome was assessed according to the following tumor and treatment variables: site (spinal/paraspinal), tumor grade, surgical margin status, and radiation dose.
*Results:* Twenty-four lesions were in the spine and 24 were in paraspinal soft tissue. Forty-two patients had malignant tumors with the following histologies: neurofibrosarcoma/malignant schwannoma (9), malignant fibrous histiocytoma (7), chondrosarcoma (5), chordoma (5), osteosarcoma (4), fibrosarcoma (3), and others (9). Six patients had benign lesions: desmoid (2), giant cell tumor (2), aneurysmal bone cyst (1), and hemangioma (1). Median tumor size was 8.5 cm (range, 1.2–18 cm). Local treatment consisted of combined surgery and radiotherapy in 45 patients and radiotherapy alone in three patients. Median radiation dose was 60.2 Gy (range, 22.4–78.6 Gy). Five patients received brachytherapy as a component of treatment, with a median implant dose of 28.7 Gy, while two patients received intra-operative radiotherapy. Chemotherapy was administered to 15 patients. Overall 5-year survival was dependent upon tumor grade: 0–1, 89%; 2, 48%; 3–4, 36% ($p = 0.10$). Overall 5-year survival was similar for spinal (72%) and paraspinal (63%) lesions ($p = 0.96$). Local control was 85% for spinal lesions and 66% for paraspinal lesions ($p = 0.59$). There was a trend towards improved local control in patients with negative surgical margins (81%) compared with patients in whom the margins were positive (55%) ($p = 0.30$). No clear relationship could be demonstrated between radiation dose and treatment outcome.
*Conclusions:* Similar to other sites, tumor grade is of prognostic importance in patients with spinal and paraspinal tumors. In spite of the large tumor size (median, 8.5 cm) seen in this patient population and the limits on surgical resection and radiation dose imposed by proximity of the spinal cord, combined modality treatment with surgery and radiotherapy produced local control in the majority of patients. Improved therapeutic strategies are necessary for patients with positive surgical margins and paraspinal tumors, in whom local control remains suboptimal.

**038 Pre-operative Radiotherapy In Adult Soft-tissue Sarcomas**

T. Morysinski, W. Ruka, P. Rutkowski
*Soft Tissue/Bone Sarcomas Department, M. Sklodowska-Curie Memorial Cancer Center Institute, Warsaw, Roentgena 5, Poland*

*Introduction:* Soft-tissue sarcomas are characterized by high local recurrence potential after inadequate treatment. The aim of the study was to analyze the results of the surgical treatment of the soft-tissue sarcomas combined with pre-operative radiotherapy.
*Materials and methods:* Between 1984 and 1999, 167 patients (84 male, 83 female; mean age, 49.2 years) treated in our institution were enrolled into the study. Twenty-five patients (15%) represented stage IA,B (according to UICC/AJCC 1987 classification), and 142 (85%) represented stage IIA,B + IIIA,B. All patients without distant metastases (MO) were included into the study consecutively. Primary tumors represented 64 (38%) cases, and local recurrence or scar after non-radical excision 103 (62%). The tumors were mainly located in lower limbs (121 patients; 72%). Most of the tumors were > 10 cm in diameter (75%). Tumor characteristics according to histological type were: 38 (23%) synovial sarcoma, 33 (20%) malignant fibrous histiocytoma, 27 (16%) liposarcoma, 23 (14%) malignant schwannoma, 20 (12%) fibrosarcoma, and 26 (15%) others. Irradiation fields contained the tumor (primary, recurrent or scar) with drain sites and the margins ~ 5 cm. The patients were divided into two groups: group I, 134 patients underwent pre-operative radiation in total dose of 2000

cGy (400 cGy/fraction) followed by surgical resection 3–5 days later; and group II, 33 patients underwent 'conventional' radiation in total dose of 4000–5000 cGy (200 cGy/fraction) with operation 6 weeks later. Median follow-up time was 36 months for survivals. The Kaplan–Meier method and the log-rank test were used for statistical analyses.
*Results:* The 5-year overall survival in group I was 69% and that in group II was 47% ($p < 0.05$). The 5-year local-recurrence free survival in group I (75%) was significantly better than that in group II (56%) ($p = 0.006$).
*Conclusions:* Pre-operative radiotherapy in adult soft-tissue sarcomas used five fractions to a total dose of 2000 cGy regimen applied 3–5 days before the operation is connected with better tumor local control rates and demonstrates benefit for overall survival in comparison with a conventional pre-operative radiotherapy group.

**098 Malignant Peripheral Nerve Sheat Tumors (Mpnst): A Retrospective Analysis On 125 Patients**

Voican D, Billard C, Terrier P, Bonvalot S, Vanel V, Le Pøchoux C, Missenard G, Le Deley MC, Le Cesne A
*Soft Tissue Sarcoma Unity, Institut Gustave Roussy (IGR), Villejuif 94805, France*

*Purpose:* To evaluate the prognostic factors in adult patients (pts) with initial localized MPNST.
*Patients:* between 1980 and 1997, 125 pts were treated at IGR. Surgery of primary tumor was performed in all cases. A complete resection was achieved in 84% of pts. Adjuvant chemotherapy and radiotherapy were given in 33 and 42% of pts, respectively. The median age of pts was 39 years (range, 15–77 years). Tumor sites were as follows: extremity, 31%; thorax, 21%; abdomen, 22%; head and neck, 18%; and pelvis, 9%. The median tumor size was 9 cm (range, 1–30 cm). Neurofibromatosis type I (NFI) is present in 20% of pts.
*Results:* Median follow-up time was 9 years (range, 3–25 years). Relapses were observed in 69% of pts (local, 56%; distant, 19%; both, 25%). The 5- and 8-year overall survival were 50 and 42%, respectively. High histologic grade, tumor size > 10 cm, NF1 association, non-extremity tumors and primary incomplete resection are unfavorable prognostic factors for overall survival in univariate analysis. High-grade MPNST, non-extremity tumors and NFI phenotype are correlated to overall survival in multivariate analysis. A high treatment-related morbidity was observed in NFI pts with MPNST.
*Conclusion:* Optimal resection seems to be the best predictive parameter for a favorable outcome in localized MPNST. Adjuvant treatments are recommended in pts with high-grade locally advanced MPNST.

**099 Is Adjuvant Radiation Therapy (Rt) Indicated After Optimal Resection For Soft Tissue Sarcoma (Sts) Of The Extremities?**

Le Cesne A, Khanfir K, Terrier P, Alzieu L, Bonvalot S, Vanel D, Le Pøchoux C
*Soft Tissue Sarcoma Unity, Institut Gustave Roussy, Villejuif 94805, France*

*Background:* The impact of adjuvant RT on both local control and distant relapse is not clearly established after wide excision of extremity STS.
*Purpose:* We performed a retrospective analysis on behavior of patients (pts) who underwent a large resection in our institution (first or second resection in case of incomplete surgery) and received or not adjuvant RT. All histological specimens were carefully analyzed and only pts with free tumoral margins (ftm) were retained for analysis. Histopathological classification was as follows: minimal RO (mRO) resection (ftm < 10 mm) and optimal RO (oRO) resection (ftm ≥ 10 mm).
*Patients:* From 1975 to 1996, 133 pts with a median age of 44 years were operated at IGR. The median tumor size was 6 cm (range, 1–18 cm). Ninety-three pts (70%) primary resected in other centers were reoperated, and residual tumor cells were found in 54% of pts. Sixty-nine pts (17 oRO and 52 mRO) received adjuvant RT and 64 pts did not (54 oRO and 10 mRO). Characteristics of pts (age, tumor size, grade, adjuvant chemotherapy) were similar in both RT and no-RT groups.
*Results:* Median follow-up time was 10 years (range, 3–25 years). Thirty-three pts had a local relapse: 11 in the RT group and 22 pts in the control group ($p = 0.01$). Grade and ftm are correlated to overall survival, and adjuvant RT is correlated to relapse free survival in univariate analysis. A positive impact of RT on local control was only seen in pts with a mRO resection ($p = 0.005$) and in pts with residual tumor cells after re-excision ($p = 0.001$). RT has no influence on 5- and 10-year overall survival.
*Conclusion:* Optimal resection seems to be the best predictive parameter for a favorable outcome in terms of local control in localized STS. Adjuvant RT is indicated in mRO resections and in case of residual tumor cells after definitive surgery, but its role after oRO resection has to be validated by a prospective randomized trial

### Posters — Basic Science/Biology

**001 Insulin And Igfs Signaling In Soft Tissue Sarcomas: Role Of Irs-1 And Irs-2 Proteins**

Bogetto L, Cervi M, Spessotto P, Mucignat MT, Canzonieri V, De Paoli A, Perris R, Colombatti A
*Centro di Riferimento Oncologico -National Cancer Institute, Aviano 33081, Italy*

Insulin is a pleiotrophic hormone with both metabolic and mitogenic effects on cells. Insulin receptor substrate 1 and 2 proteins (IRS-1/2) are the principal intracellular substrates of insulin (and IGFs) receptor(s) that undergo tyrosine phosphorylation immediately after ligand stimulation. They act as docking proteins transmitting the receptor signal to downstream effector proteins containing SRC homology 2 domains, such as the p85 regulatory subunit of phosphatidyl-inositol 3-kinase (PI 3K), and the growth factor receptor binding protein 2 (Grb2). Together, these intermediate signals stimulate a variety of different biological effects, including glucose transport, glycogen synthesis, gene expression, cell migration, and cell proliferation. Although IRS-1 and IRS-2 contain common functional units and share some unique signalling pathways, it is not yet clear if they play redundant or selective roles in insulin action in different tissues, even if data provide evidence that they are not functionally interchangeable substrates. These two proteins show differential expression in several carcinoma tissues and cell lines: IRS-1 results highly expressed in hepatocellular and breast carcinomas, whereas pancreatic carcinomas overexpress IRS-2. Little is known about the role of IRS-1 and 2 in soft tissue sarcomas (STS) as well as about the insulin and IGFs signalling in the biology of these tumours. We have analyzed the expression pattern of IRS-1, IRS-2, IR, and IGF-1R in a number of cell lines derived from our patients, and have selected two malignant fibrous histiocytoma-like (MFH) sarcoma cell lines (97441 and 98337) for their differential expression of IRS-1 and IRS-2 molecules and their different behaviour *in vitro* under basal conditions or under stimulation with insulin. From each tumour, three morphologically different sub-lines, named A, B and C, were obtained. 97441 A and 98337 A cells show high expression of IRS-1, whereas IRS-2 predominates in 97441 B cells; 98337 B cells lack both IRS-1 and IRS-2. The C populations of 97441 and 98337 show only a weak IRS-1 expression and no IRS-2. The

IGF-1R expression is almost the same among the different sublines, whereas a slight increase of IR appears only in 97441 B cells. Extending our study on *in vitro* migration and invasion of these cells, we found that insulin does not modify the migration activity of 98337 A and B (on different ECM molecules), whereas it considerably increases the invasive capability of 98337 A. These results indicate a possible role of insulin in dissociating the migration and the invasion signalling pathways. To study the effect of IRS molecules that are differentially expressed, we are transfecting these cell lines with vectors containing sense or antisense IRS-1/2.

**002 Ecm Interactions And Production Of Proteolytic Enzymes In Malignant Fibrous Histiocytomas**

Spessotto P, Mucignat MT, Wasserman B, Burino I, Bogetto L, Cigana A, Frustaci S, Perris R, Colombatti A

*Centro di Riferimento Oncologico, National Cancer Institute, Aviano 33081, Italy*

We have established several novel cell lines from surgical specimens of recurrent metastases of different type of soft tissue sarcomas, focusing our attention on malignant fibrous histiocytomas (MFH), which were found to undergo spontaneous growth behaviour-conversion *in vitro*, loosing progressively their anchorage dependence without incurring into apoptotic processes. From each of two of this type of sarcoma, three sub-lines different for morphological criteria were obtained: we defined large and big spindle cells as 'sub-line A', smaller and less adherent fibroblastic cells as 'sub-line B', and cells growing in suspension as 'sub-line C'. The malignancy of these cells, evaluated by a colony forming unit assay, was higher for sub-lines B and C than A in both cell lines. In order to verify whether these sarcoma cells could represent a useful model to study three different steps in the process of metastasis, we looked for their binding activity to extracellular matrix (ECM) molecules, capacity to invade and to produce proteolytic enzymes. As regards ECM static and dynamic interactions, we found that the three sub-lines differed for the ability to adhere to a variety of ECM substrates and for migratory/invasive behaviour, but these differences were not the same for the two MFHs. The distinct behaviour of the sub-lines was also observed for the production of degradative enzymes. Zymographic analyses were performed for the production of the metalloproteinases 2 and 9 (MMP-2 and MMP-9), and of the urokinase-type activator of plasminogen (uPA), which are highly correlated with malignancy in carcinomas. The sub-lines of the two sarcomas displayed a production pattern different either under normal culture conditions or after activation; it seemed that the induced production of MMP-9 and the increase in uPA secretion were correlated with a more aggressive phenotype. Extending our study on other proteolytic enzymes with a RT-PCR approach, we observed that the MMP expression of MFH sub-lines was identical for MT1-MMP, MMP-3 and MMP-13 but completely different for MMP-7 and MMP-11. In conclusion, our results suggest that interactions with ECM and MMP expression and production could be consistent 'markers' to distinguish apparently similar sarcomas in different categories of biological aggressiveness/malignancy.

**008 Doxorubicin And Valspodar (Psc 833) Combined Therapy In Canine Osteosarcoma According To P-glycoprotein Expression**

Cagliero E[1], Scotlandi K[2], Morello E[3], Manara MC[2], Buracco P[3], Baroetto R[1], Ferracini R[1]

[1]*Istituto per la Ricerca e la Cura del Cancro, Candiolo 10060, Italy,* [2]*Istituti Ortopedici Rizzoli, Bologna 40136, Italy, and* [3]*Dipartimento di Patologia Animale,Torino 10126, Italy*

*Background:* Osteosarcoma of the appendicular skeleton is one of the most life-threatening canine neoplasms. Only 10% of dogs treated with surgery alone survive 1 year after treatment. Even with the current most effective treatment, surgery combined with adjuvant chemotherapy, 65% of dogs die within 1 year and 85% within 2 years. In human osteosarcoma, it has been shown that P-glycoprotein (Pgp), which confers multidrug resistance (MDR) to some anticancer agents, including doxorubicin, might be a major cause of failure of disease control. We evaluated Pgp expression in osteosarcoma of dogs treated with combined administration of doxorubicin and the MDR modulator PSC 833, and examined the pharmacokinetic between the two drugs.
*Patients and methods:* Dogs presenting appendicular osteosarcoma without detectable lung metastaseis at the time of diagnosis underwent surgery consisting of amputation or of limb sparing techniques when applicable. Post-operatively, dogs received doxorubicin alone in the first cycle, and in the following four cycles they received a combination of doxorubicin and PSC 833. According to predictable pharmacokinetic interactions, doxorubicin dose was reduced of 30%. Several blood samples were collected for the determination of doxorubicin and PSC 833 levels. Pgp expression was monitored on tumor tissue samples from surgical excision prior to treatment using immuno-histochemistry with anti-Pgp monoclonal antibodies.
*Results:* All the dogs treated were tested for Pgp expression prior to filing for treatment. Pgp was found to be expressed in 13/15 (87%) of the samples. At the time of doxorubicin administration, adequate blood concentrations (i.e. 2.5 mM) of PSC 833, which are needed to reverse MDR, were achieved at the dose of 7.5 mg/kg/day. Dogs did not show clinical or laboratory findings of toxicity. In order to maintain the same exposure and to control dose-related toxicity of doxorubicin, we verified that similar plasma levels of doxorubicin were achieved after both the standard full dose and with the reduced dose in animals given PSC 833.
*Discussion:* Current treatment of canine osteosarcoma using surgery and adjuvant therapy is not satisfactory. Combination therapy with doxorubicin and PSC 833 allows a decrease in doxorubicin dose infusions with equivalent therapeutic exposure. The high incidence of Pgp expression in tumor samples prior to treatment confers to the study a rationale for downmodulation of MDR. Further investigations are needed to evaluate concentrations of doxorubicin in the tumor tissues and to validate the efficacy of the treatment in ongoing clinical trials.

**104 P16INK4A Gene And Osteosarcoma**

Benassi MS, Molendini L, Gamberi G, Merli M, Ragazzini P, Magagnoli G, Picci P

*Laboratory of Cancer Research, Rizzoli Institute, 40100 Bologna, Italy*

*Introduction:* The p16INK4A putative tumor suppressor gene expresses two different mRNA transcripts, alpha and beta. The alfa transcript p16INK4a, a cyclin D/cdk4 inhibitor, prevents inactivation of the retinoblastoma protein (pRb). The beta transcript p14ARF, protects p53 protein by binding with MDM2, and upregulates the p21 protein.
*Aim:* In order to define the role of the p16INK4A gene in human osteosarcoma (OS), we studied gene status and expression of the molecules involved in p16 regulatory system.
*Materials and methods:* Thirty-five high-grade formalin-fixed, paraffin embedded and frozen OS samples were studied by immunohistochemistry (IHC) and immunoblot. Exons 1 and 2, 5′CpG island methylation of the p16INK4A gene and semiquantitative analysis of the CDK4 gene were performed by specific PCR.
*Results:* IHC analysis revealed an increased expression of p16 protein in 14/35 OS. p16 staining intensity ranged from moderate to strong. Of these, six with pRb positive expression simultaneously showed cdk4 overexpression (6 x) and CDK4 gene amplification (8–10 x). No deletions or mutations were found in p16INK4A gene, but nine p16-negative OS showed 5′CpG methylation. A weak to moderate expression of p14ARF protein was detected in 15/35 OS; of these, 11 had active p53 and 10 immunoreacted to p21protein.
*Conclusions:* These results suggest that two important cell cycle regulatory pathways are involved in OS pathogenesis.

**Intramural Oxygen Status In Soft Tissue Sarcoma: Prognostic Value With Respect To Different Treatment Modalities**

Peter Hohenberger, Mathias Schlafke, Birger Bida
*Divison Of Surgery and Surgical Onocolgy, Charite, Humboldt University of Berlin, Germany*

*Purpose:* Intramural oxygen partial pressure ($pO_2$) was reported to be predictive for the likelihood of distant metastases in human soft tissue sarcoma if $pO_2$ was < 10 mmHg (Brizel, *Cancer Res* 1996; 56:941). However, those patients were treated by 50 Gy irradiation, and it is well known that oxygenation interferes with the efficacy of radiation therapy. We were interested to know whether $pO_2$ is also of predictive value in patients treated surgically and represents an independent prognostic marker.
*Methods:* In 73 patients suffering from soft tissue sarcoma of the limb or trunk, intratumoral oxygen partial pressure was measured polarographically. We used an $O_2$-sensitive fine needle probe (KIMOC, Eppendorf, Germany; $n$ = 41). The mean of $pO_2$, range and proportion of the hypoxic tissue fraction (< 5 mmHg) was evaluated and depicted in histogramas of 200 measurements per tumor. The tissue temperature was recorded along the puncturing canal. Another 32 patients were analysed by use of an intratumoral implant probe $p(ti)O_2$ with a sensitive surface of 7.1 $mm^2$ (LICOX; Med. Systems Corp., Greenvale, NY, USA) during neoadjuvant combined modality therapy. Tumor vascularisation was divided into hyper-, iso-, hypo-, and avascular pattern according to angiogram and compared with the oxygenation status. DFS was measured by the Kaplan–Meier method, and the log-rank technique was used to compare DFS of different groups of patients. Spearman correlation coefficients were calculated to examine the relationship of tumor oxygenation and vascularity.
*Results:* The mean $pO_2$ values were from 0.6 to 69 mmHg. G1 tumors showed a markedly higher $pO_2$ (51.4 mmHg) in contrast to G2 and G3 tumors with 32 mand 29 mmHg, respectively. The proportion of hypoxic fractions was significantly lower in G1 tumors (6.3%) versus G2/3 tumors (15 and 18%, respectively; $p$ = 0.01) Liposarcomas showed the highest $pO_2$ values and there was no correlation to vascularity as determined by angiogram. There were no significant differences in the proportion of patients developing distant metastases whether their primary tumors showed mean $pO_2$ exceeding or being less than 10 mmHg. Measurements of $pO_2$ obtained during neoadjuvant treatment, however, showed considerable differences between histological subtypes but also within the same group.
*Conclusions:* Tumor $pO_2$ is not a general marker of risk of distant metastases in soft tissue sarcoma if patients are treated without radiotherapy. However, extreme differences between tumors of identical type may severely interfere with drug uptake f.e. during neoadjuvant treatment. Intratumoral $pO_2$ might be very useful tool to control for effects of systemic or regional drug treatment of sarcomas.
*Acknowledgement:* Part of this work was supported by grant of Deutsche Forschungsgemeinschaft SFB 273/C 13.

### Posters — Medical Oncology

**010 Results Of A T10-derived Protocol Based On Preoperative High-dose Methotrexate With Individual Dose Adjustment In The Treatment Of Primitive Osteosarcoma**

Duffaud F, Digue L, Volot F, Bouvier C, Curvale G, Poitout D, Favre R
*Hôpital La Timone, Marseille 13385, France*

From January 1987 to December 1997, 42 adolescents and adults with high-grade osteosarcoma were treated at La Timone University Adults Hospital with a T10-derived protocol using high-dose methotrexate (HDMTX) with individual dose adjustment. The male/female ratio was 3:1 and the median age 24 years (range, 14–53 years). Primary tumor sites included extremities (90.4%), axial bones (7.2%), and head and neck (2.4%). Seven patients (16.7%) presented with initial lung metastases. Pre-operative chemotherapy included 4-weekly cycles of HDMTX with individual dose adjustment followed by citrovorum factor rescue. HDMTX was administered in 8-hour perfusion to achieve a serum MTX concentration of 1.000 mmol/l ($C_{max}$) at the end of the infusion. Definitive surgery was performed 10–14 days after the last HDMTX cycle. Twenty-nine patients (72.5%) had conservative surgery and 11 patients (27.5%) underwent amputation. The histologic response to pre-operative chemotherapy evaluated according to the method of Huvos grading system was rated 'good' (≥ 90% tumor necrosis) in 11.2% of patients or 'poor' (< 90% tumor necrosis) in 88.8% of patients. 'Good' responders received post-operative chemotherapy based on HDMTX, pirarubicin and BCD (Bleomycin-Cyclophosphamide-Dactinomycin). whereas 'poor' responders received a 'salvage' regimen including ifosfamide, pirarubicin, cisplatin ± etoposide during 18 weeks. No toxic death occurred and severe toxicities (WHO grade III/IV) due to chemotherapy were moderate. Individual MTX dose adjustment according to the patient's pharmacokinetic profile was optimum (mean $C_{max}$-obtained was 1001 ± 75.4 μmol/l). Mean delivered dose of HDMTX was higher than that recommended by Rosen (13.1 g/$m^2$ versus 8–12 g/$m^2$). With a median follow-up of 55 months (range, 11–139 months), the 5- and 8-year overall survival (OS) rates were 65.7 and 61.3% for the entire group. For the 35 patients with localized disease at diagnosis, the 5- and 8-year OS rates were 75.8 and 70.4%. For the same group, event-free survival (EFS) rates were 64.6 and 55.5% at 5 and 8 years. Fourteen patients (33.3%) relapsed: eight patients developed lung metastases, two developed a local recurrence and four presented both. The difference in OS and EFS rates at 5 years between 'good' and 'poor' responders was not significant. In multivariate analysis, tumor size, dose intensity and pre-operative MTX dose intensity were independent prognostic factors for OS ($p$ = 0.0004, 0.04, 0.007). Our OS and EFS rates are similar to those reported in recent studies despite the low rate of 'good' responders to pre-operative chemotherapy obtained. Our post-operative 'salvage' chemotherapy regimen is effective for 'poor' responders yielding identical EFS and OS rates as those of 'good' responders.

**013 Preliminary Results Of A Phase 2 Trial Of Oral Piritrexim In Patmients With Malignant Fibrous Histiocytoma (Mfh)**

Patel SR, Jenkins J, Hays C, Beech J, Burgess MA, Papadopoulos NE, Plager C, Pisters PWT, Feig BW, Hunt K, Pollock RE, Benjamin RS
*The Sarcoma Center, University of Texas M.D. Anderson Cancer Center, 1515 Holcombe Blvd., Houston, TX 77030, USA*

Piritrexim is a Epid-soluble antifol with a very high affinity for dihydrofolate reductase. It enters the cell by passive diffusion and has been shown to inhibit growth of methotrexate-resistant cells *in vitro*. A phase 1 trial of the intravenous preparation revealed a response in a patient with MFH. We studied the oral preparation in patients with metastatic MFH who had received one prior chemotherapy regimen. Patients were required to have histologic confirmation, measurable/evaluable disease, ECOG PS ≤2 and reasonable organ function. Piritrexim was administered at a starting dose of 25 Ing p.o. TID x 5 consecutive days each week for 3 weeks followed by 1 week rest off treatment. A total of 10 patients have been treated at our center and are evaluable for response and toxicity. The median age is 55 years (range, 29–77 years). There were five males and five females, all with ECOG PS = 1. All patients had one prior chemotherapy regimen, five patients had prior radiation and 10 patients had prior surgery. Two patients have shown a radiographic response. One patient with a primary tumor in the right thigh (and lung metastases) had a 53% reduction in the measurement of the primary tumor, thus qualifying for a partial response. Another patient with metastatic disease in the lungs had a 26% reduction in the size of the lung nodule after 5 months of treatment. Unfortunately, his treatment had to be discontinued since the sponsor terminated their plans for further development of the drug. Follow-up CT scan of the chest on this patient 6 weeks off treatment prior to planned surgery reveals a near complete response with only one tiny residual abnormality. A total of 26 cycles are evaluable for toxicity. One patient each in one cycle each experienced grade 3 nausea, vomiting and fatigue. Overall, the treatment was well tolerated. In conclusion, oral piritrexim does seem to have some biologic activity in patients with MFH. Unfortunately, our trial has been terminated as per the sponsor's request, and therefore no more data will be available to truly assess the activity of this drug.

**014 Extraskeletal, Adult Ewing Family Of Tumors (Eft): Combined Treatment In 42 Patients**

Casali PG, Bertulli R, Santoro A, Lombardi F, Spreafico C, Dileo P, Gandola L, Navarria P, Gronchi A, Quagliuolo V, Valente M, Brega Massone P, Mastore M, Rolfo CD, Rasponi A, Cataldo I, Pilotti S, Azzarelli A
*Istituto Nazionale Tumori, Milan, Italy*

*Background:* Only small series of 'extraskeletal' Ewing's sarcoma or pPNET are available, after first report in 1975. Both skeletal and extraskeletal cases in adults are also rare.
*Intervention:* From 1988, over 10 years, 42 chemoradiotherapy-naive patients in the adult age (range, 16–58 years; median, 24 years) with diagnosis of extraskeletal Ewing's sarcoma or pPNET were prospectively treated with: primary chemotherapy (Ifosfamide + Epirubicin + Vincristine, x 4–7); surgery, if feasible, in non-metastatic patients; radiotherapy ± Cisplatin and Vincristine; consolidation chemotherapy (Ifosfamide + Dacarbazine + ActinomycinD, or Etoposide + Cisplatin or Ifosfamide, or high-dose Ifosfamide, x 2–6).
*Patient characteristics:* Of 42 patients, 36 had only local disease: 31 had visible disease, five were NED at entry after diagnostic surgery; tumor originated from thorax (12), limbs (11), paravertebral region (7), pelvis (3), and head and neck (3). All ED patients are evaluable for response to primary chemotherapy, save for one who received symptomatic concurrent chemo-radiotherapy. Six patients had evaluable metastatic disease (to lungs ± liver ± bone). Tumor size was > 10 cm in 55% of isolated local lesions. Median follow-up for all patients is 86 months.
*Results:* Response to chemotherapy was achieved in 72% of 36 evaluable patients. Among 30 patients with local disease, complete responses were 30% (6/9 were pathologically detected). The 10-year actuarial RFS was 46% in patients with local disease. Patients with limb and head and neck lesions had a RFS of 70%, versus 31% in patients with thoracic, paravertebral and pelvic disease. None of six patients with metastatic disease stayed disease-free.
*Conclusions:* (1) In this series of adult patients with extraskeletal EFT, long-term RFS was superimposible to childhood series of skeletal origin accrued in the same years, particularly if tumor size is considered. (2) The complete response rate seems lower. This might be relevant in mutuating treatments from protocols for skeletal disease. (3) A subgroup of patients with worse prognosis, due to origin from thorax, paravertebral region, and pelvis, was identified in this series. This is worth confirming. If so, these high-risk patients would need, and certainly metastatic patients do need other/new approaches.

**015 Aggressive Fibromatosis: A Conservative Approach To Inoperable Disease Based On Low-dose Chemotherapy With Methotrexate + Vinblastine**

Azzarelli A, Gronchi A, Bertulli R, Casali PG, Lozza L, Baratti D, Pennacchioli E, Rasponi A, Dileo P, Rolfo CD, Pilotti S
*Istituto Nazionale Tumori, Milan, Italy*

*Intervention:* Over a 10-year span from 1989, 30 patients (pts) with inoperable aggressive fibromatosis were conservatively treated with chemotherapy alone, in the framework of a pilot study on Methotrexate + Vinblastine.
*Patients:* Thirty patients (median age, 27 years; M/F, 13/17), with primary (20%) or recurrent (80%) inoperable aggressive fibromatosis (arising from scapular girdle in 12 pts, limbs in eight, head and neck in three, pelvis in three, paravertebral regions in two, chest wall in two), were treated conservatively, by administering Methotrexate 30 mg/m$^2$ + Vinblastine 6 mg/m$^2$, given every 7–10 days for a median interval of 1 year (range, 4–27 months). Only two pts underwent surgery, with a debulking intent in both cases, followed by radiation therapy. Radiotherapy was given to another two pts as well. All the other pts received chemotherapy alone.
*Results:* Eighteen patients (60%) showed stable disease or minor tumor shrinkage with symptom relief. Partial response was detected in 12 patients (40%). While no complete response was observed, no patient had tumor progression during treatment. After a median follow-up of 75 months, the 10-year actuarial progression-free interval is 67%. Toxicity was mild, including neutropenia, increase in transaminases, paraestesias. Subjective intolerance to such a long-term treatment was of major concern: in 15 pts, the decision was shared to stop their treatment after less than 40 courses. However, progression was detected in four out of six pts who stopped after less than 20 courses.
*Conclusions:* A low-dose chemotherapy regimen like Methotrexate + Vinblastine allowed us to conservatively manage aggressive fibromatosis in two-thirds of pts with inoperable disease. Optimal treatment length is left to be determined, although it seems that some threshold for effectiveness does exist. Tolerability might increase with recently proposed similar regimens, like Methotrexate + Vinorelbine. Low-dose chemotherapy stands amongst currently available choices in aggressive fibromatosis, and may constitute a reasonable option in the subset of pts with inoperable disease.

**016 Metastatic Angiosarcoma – Recent Treatment Results**

Harmon DC
*Massachusetts General Hospital, Boston, MA 02114, USA*

Metastatic angiosarcoma is often viewed as an unfavorable form of sarcoma. We were surprised to find two long-term remissions among patients treated aggressively as well as a string of responders to milder chemotherapy, so we reviewed the charts of all patients evaluated at MGH in the past 8 years. Of 20 patients seen in consultation for metastatic angiosarcoma, four declined any therapy, primarily because of older age or complicating medical illnesses. Two patients were treated elsewhere and follow-up is not available. The 14 remaining patients had 17 courses of treatment. There were 11 major responses, seven that were complete or near-complete, with two possible cures. One patient treated with carboplatinum/taxol for undifferentiated tumor got a 4-month near-CR before dying of toxicity shortly after a pathology review at MGH showed angiosarcoma. Of 10 patients who received MAID chemotherapy (mesna, doxorubicin, ifosfamide, dacarbazine), three had CRs (complete responses) including two continuous remissions of over 6 years. One of these long-term survivors was treated with radiation to his partially resected maxillary sinus primary and consolidated with MAID and a carboplatin/cyclophosphamide stem cell transplant after resection of a lymph node metastasis. The other survivor had two brain metastases resected at diagnosis, then radiation to a hard-to-diagnose scalp primary, then MAID for lymph node metastases. The third CR lasted only 4 months after MAID alone, but another patient with a PR (partial response) to MAID alone at 5+ months is approaching CR. Another patient, who got MAID followed by stem cell transplant, relapsed and then got a 1-year near-CR to single-agent vinorelbine, relapsed and got a second 1-year near-CR to vinorelbine. Thereafter, one patient, who had no response to MAID, got a 5-month PR to vinorelbine. Three patients too old to get MAID received vinorelbine alone instead, and one got a near-CR of 6+ months and two had PRs of 13 and 7+ months. Aggressive multi-agent, multi-modality treatment for those who can tolerate it and milder single-agent vinorelbine for those who cannot may be an appropriate policy for dealing with metastatic angiosarcoma.

**078 Expression Of Fas Ligand And Fas In Human Soft Tissue Sarcomas Before And After Hyperthermic Isolated Limb Perfusion With Tnfα And Melphalan**

Komdeur R, Molenaar WM, de Jong S, Hoekstra HJ, Plaat BEC, Van den Berg E, Van der Graaf WTA
*University Hospital Groningen, 9700 RB, The Netherlands*

*Introduction:* Hyperthermic isolated limb perfusion with TNFα and melphalan (HILP-TM) has improved the limb salvage rate for primarily irresectable soft tissue sarcomas (STS) of the extremity to > 75%. Apart from the effect of TNFα on the tumor-associated vasculature, it may also act as a cytotoxic agent either directly or indirectly. TNFα can upregulate the expression of Fas Ligand (FasL) and Fas; FasL is a member of the TNF family and rapidly induces apoptosis after binding to its receptor Fas. TNFα and cytostatics may (partially) exert their anticancer effect via the Fas–FasL pathway.
*Objectives:* (1) To determine whether the expression of FasL and Fas is changed after HILP-TM. (2) To evaluate the relationship between FasL and Fas expression, tumor response and apoptotic index.
*Patients and methods:* Thirty-five patients with a primarily irresectable extremity STS underwent HILP with TNFα (3–4 mg) and melphalan (10–13 mg/l limb volume). The median period between HILP and resection of the tumor remnant was 60 days (range, 12–103 days). Tumor samples were collected from the diagnostic pre-HILP specimen and from the post-HILP resection specimen. For assessment of tumor response and immunohistochemical analysis of FasL and Fas, see Tables 1 and 2. Using the TUNEL method, the apoptotic index was quantified as the percentage of apoptotic tumor cells.
*Results:* Six patients had CR (17%), leaving no viable tumor tissue for immunohistochemistry; 25 patients revealed a PR (71%); four patients had NC (11%). Before HILP, 15/33 (45%) and 15/19 (79%) samples scored positive for FasL and Fas, respectively (Figs. 1 and 3). After HILP, the samples for FasL and Fas stained positively in 11/28 (39%) and 13/15 (87%), respectively (Figs. 2 and 4). For evaluable paired samples taken before and after HILP, no significant changes in FasL or Fas were seen. The mean percentage of apoptotic cells increased from 0.91 to 2.09% after HILP ($p < 0.05$). Statistical analysis revealed no correlation between FasL/Fas expression and tumor response or apoptotic index.
*Conclusions:* No significant changes were found in FasL and Fas expression after HILP-TM. FasL and Fas expression did not predict the tumor response to HILP-TM or apoptosis. However, these results may be influenced by the protracted period between HILP and tumor resection and absence of evaluable tumor tissue after HILP-TM in patients with CR.

**085 Influence Of Locally Advanced And Recurrent Disease On Survival Of Patients With Advanced Soft Tissue Sarcomas. A Retrospective Analysis Of The Eortc Soft Tissue And Bone Sarcoma Group (Stbsg)**

Reichardt P, Van Glabbeke MM, Mouridsen HT, Verweij J, Radford J, Rodenhuis S, Azzarelli A, Le Cesne A, Buesa J, Keizer HJ, van Oosterom A, Kirkpatrick AL, Nielsen OS
*EORTC Soft Tissue and Bone Sarcoma Group and EORTC Data Center, Brussels, Belgium*

To establish the influence of locally advanced, recurrent and metastatic disease on survival of patients with advanced soft tissue sarcomas, a retrospective analysis using the database of the EORTC STBSG was conducted. A total of 1622 patients treated in five different studies with anthracycline based chemotherapy as first-line treatment of advanced soft tissue sarcomas were included in the analysis. Patients were grouped into five categories: locally advanced, recurrent disease, metastatic disease, locally advanced plus metastases, and recurrent disease plus metastases. Information on age, sex, performance status, histology by pannel diagnosis, grade, and time of recurrence or metastases since first diagnosis are available on all patients.
Overall response rates in the five categories were 26, 32, 28, 24, and 19%, respectively, which was significant with a $p$ value of 0.016 (overall chi-square test).
The median overall survival in the five categories was 416, 345, 388, 275, and 303 days, respectively, which was significant with an overall $p$ value of 0.0001 (log-rank test). Univariate comparison of patients with locally advanced disease and patients with recurrent disease showed a significant difference with a $p$ value of 0.0127. Comparing patients with local recurrence alone with patients with metastases ± local or recurrent disease did not reveal a statistically significant difference ($p = 0.8397$). Patients with locally advanced disease versus all other patients had a significantly superior survival with a $p$ value of 0.0001. In the multivariate analysis, using the published model for risk factors (Van Glabbeke *et al.*, *J Clin Oncol* 1999; 17:150–157), the independent prognostic value of three additional variables was tested: presence of locally advanced disease, presence of recurrent disease, and presence of metastases. As expected, age, grade, liver involvement and performance status remained highly significant. Looking at the new variables, only presence of recurrent disease was an independently statistically significant negative factor for survival with a $p$ value of 0.0325.
*Conclusions:* Response rates are the lowest in patients with locally advanced disease plus metastases and especially recurrent disease plus metastases, probabely due to higher tumor burden. Presence of recurrent disease represents an independent risk factor associated with inferior survival and should therefore be considered as a stratification factor in trials where the principal end-point is survival.

**086 Chemotherapy For Metastatic Chondrosarcoma Of Bone: Single Institution Experience In 14 Patients**

Pink D, Tilgner J, Dörken B, Reichardt P
*Department of Hematology, Oncology and Tumorimmunology, Robert-Rössle-Klinik, Universitätsklinikum Charitø, Humboldt-University, 13122 Berlin, Germany*

Chondrosarcoma is a rare malignant tumor of cartilage producing cells and the second most common primary neoplasia of bone after osteosarcoma. The majority of patients are between 30 and 60 years of age. The pelvic girdle is the predominant site of primary disease. Most tumors are well differentiated and surgery is the only established treatment option. High-grade chondrosarcomas, however, tend to develop distant metastases mainly in the lungs. Chemotherapy for metastatic disease has not been investigated to any extent and published data do not exist beyond occasional case reports.

From 1993 to 1999, 14 patients with metastatic chondrosarcoma of bone not amenable to surgical resection of metastases recieved chemotherapy in our institution. The median age of the patients (seven males and seven females) was 54 years (range, 31–71 years). Primary tumors were mainly located in the pelvis (five patients). Other primary locations were equally distributed between thorax, upper and lower limb (three patients each). Histology showed high-grade lesions in nine patients, low grade in two patients and dedifferentiated chondrosarcoma in three patients. Metastases were predominantely located in the lungs (13 cases). One patient presented with liver metastases only. Additional locations were bone, skin, lymph nodes and brain (one case, respectively). Median time interval from first diagnosis to metastases was 10 months (range, 0–32 months).

Chemotherapy consisted of dose-intensive epirubicin/ifosfamide combination in eight patients, high-dose ifosfamide in one patient, doxorubicin, ifosfamide, cisplatin ± methotrexat in four patients and another anthracyclin-based combination in one patient. Six patients recieved second-line chemotherapy with high-dose ifosfamide (three patients) and cisplatin/gemcitabine (three patients). Three patients were treated with oral trofosfamide as maintenance therapy.

Treatment results of first-line chemotherapy were a partial response in three patients, stable disease in five patients and progression in six patients. Response duration was 6, 6 and 12 months. Disease stabilisation lasted from 4 to 12 months. Median overall survival from diagnosis of metastases was 20 months for PR and SD patients (range, 6–80+) and 10.5 months (range, 4–34) for patients with progressive disease.

*Conclusions:* Chemotherapy resulted in a response rate of 21% and an overall progression arrest rate of 57%. Response to treatment resulted in a doubling of the overall survival time from 10.5 to 20 months. Chemotherapy should be considered for patients with metastatic chondrosarcoma with inoperable disease or as part of a multimodality treatment regimen.

**097 Osteosarcoma Over The Age Of 40**

Grimer RJ

*on behalf of the European Musculoskeletal Oncology Society. The Royal Orthopaedic Hospital Oncology Service, Bristol Road South, Birmingham B31 2AP, UK*

*Purpose:* To ascertain whether patients over the age of 40 have a different disease pattern and prognosis to younger patients with osteosarcoma.

*Background:* Osteosarcoma is principally a disease of adolescents, but 13% of all patients with osteosarcoma will be over the age of 40 and, to date, have been excluded from most national trials of treatment of osteosarcoma. A collaborative study from EMSOS has collected data on these patients.

*Material:* Data on 486 patients have been contributed from 13 centres. The ages ranged from 40 to 89 with a mean of 54; 275 were male and 211 female. The most common sites were femur followed by pelvis and tibia, and 84 had a secondary osteosarcoma: 42 related to Paget's disease and 42 secondary to radiation. Thirty patients had 'low grade' osteosarcoma, the remainder having high grade tumours, of which 57 had metastases at presentation. Almost half the patients had chemotherapy. Surgery consisted of amputation in 118 and limb salvage surgery in 252, with 113 having endoprostheses and 15 having allografts. One hundred and eighty-four were felt to have had adequate surgical margins and 102 had close or inadeqaute margins. Eighty-five patients had radiotherapy, of which 41 were palliative.

*Results:* The overall survival rates were 37% at 5 years falling to 27% at 10 years. The 5- and 10-year survival rates for stages 1,2 and 3 turnours were, respectively: 78 and 62%, 41 and 28%, 9 and 9%. Patients over the age of 60 and patients who did not have adequate treatment, not surprisingly, fared worse, as did all patients with secondary osteosarcoma. The median survival time for patients with Paget's osteosarcoma was 7 months and that for radiation sarcoma was 12 months. For patients with stage 2 tumoursn the effect of age was such that the 5-year survival rates were 45% for those younger than 60 and 28% for those over 60. There was little information about effectiveness of chemotherapy but there was a trend for those with a good response to fare better than those without. Local recurrence arose in 7% of those having amputations and 22% of those having limb salvage surgery. It was closely related to margins of excision with 27% of those having inadequate margins developing local recurrence compared to 12% of those with adequate margins.

*Conclusion:* Osteosarcoma over the age of 40 requires just as much care and multidisciplinary working as osteosarcoma in the younger age group. Patients with secondary osteosarcoma have a dismal outlook. Chemotherapy and effective surgery remain the mainstay of treatment.

**110 Feasibility Of Pre Or Post-operative Combined Chemotherapy And Radiation-therapy In Adult Soft Tissue Sarcomas Of Extremities**

Frustaci S[1], Buonadonna A[1], Boz G[1], Berretta M[1], Rupolo M[1], Bertola G[1], Innocente R[1], Gherlinzoni F[2], De Paoli A[1]

*[1]CRO-IRCCS, National Cancer Institute, Aviano, and [2]Orthopedic DivISION, General Hospital, Gorizia*

Adjuvant chemotherapy (CT) impacts favorably on DFS and OS. Pre or post-operative radiation therapy (pre-, post-op RT) and wide excision allow limb preservation with local control success comparable to radical (ablative) surgery. However, the long interval between diagnosis and start of CT could be responsible for the appearance of resistant cellular clones and possibly micro-metastases. Therefore, the anticipation and combination of CT with RT could be worthwhile in terms of prognosis. Based on this hypothesis, we started a pilot study to evaluate the feasibility and toxicity of this integration.

*Methods:* Eligibility: high grade (grade 3–4 Broder), polymorphous, sub-fascial spindle cell sarcomas of extremities and/or girdles, size > 5 cm or any dimension if a relapse; age > 16 and < 65 years; PS < 2 ECOG; no previous CT or RT. Chemotherapy: Epirubicin, 60 mg/m$^2$, days 1–2; Ifosfamide, 3 g/m$^2$, days 1–3, with fractionated equivalent dose of Mesna; G-CSF, 300 mg/die s.c. from day +9 to day +16. Treatment was given every 21 days. Radiation therapy: Conventional treatment divided in two phases in the pre-operative setting: 22 Gy/11 Fr between cycles 1 and 2 of CT; 22 Gy/11 Fr between cycles 2 and 3 of CT; and a third phase of 16 Gy/Fr between cycles 4 and in the post-operative setting.

*Results:* From September 1996, 14 patients have been enrolled, seven as neo-adjuvant therapy and seven as adjuvant therapy. Toxicity: On 41 evaluable cycles of CT: leucopenia grade 3–4, 51%; thrombocytopenia grade 3–4, 14%; anemia grade 3–4, 9%; nonhematological toxicity grade 3–4, 32% (stomatitis 4%); cutaneous (erithema 21%), mucositis (proctitis 7%). All seven patients treated in pre-op setting underwent conservative surgery; one patient had major complication (infected seroma). No complications were noted in post-op group.

*Feasibility:* The average median dose intensity of CT was 84% (60–100%). The RT was performed in foreseen doses and time interval in 13 of 14 patients. In one patient, RT was delayed due to thrombocytopenia (field of RT included great vessels). The median time interval between biopsy and start of chemotherapy in our adjuvant previous study was 92 days (range, 17–288 days), while in this experience it was 9 days (range, 5–34 days).

*Preliminary Conclusions:* Integration of CT with RT allowed a preservation of dose intensity of 84% with a compliant toxic profile. The accrual is ongoing in order to obtain sufficient data on toxicity to define a new neo-adjuvant cooperative randomized trial.

### Posters — Pediatric Oncology

### 081b Strategy Of Treatment In Desmoid Tumors (Aggressive Fibromatosis In Children And Adults): To Avoid Absolutely Radiotherapy

Delepine N, Alkallaf S, Markowska R, Cornifie H, Belarbi L, Delepine G
*Oncologic Paediatric Unit, Avicenne University Hospital, 125 Bd de Stalingrad, 93009 Bobigny Cedex, France*

Desmoid tumor is a histologic benign tumor. Nevertheless, frequent relapses are able to threaten life or conservation of limb. To point out the best indications of treatment, we reviewed our cases. From 1981 to 1998, we treated 50 patients with fibromatosis (mean age, 28 years; range, 1–70 years). Locations were inferior limb (19), superior limb (17), axial (9), head and neck (5). Seventeen patients were seen at first hand, 33 with relapses. Treatment was adapted to each patient, in function of age, history, and risks of spontaneous evolution.

*En bloc* extratumoral resection was performed each time when surgery did not expose to heavy functional sequellaes (22). The other patients were treated by large resection, but never invalidant. Fourteen patients received pre- or/and post-operative chemotherapy. TEn received Interferon cc, and nine received tamoxifen.

*Results:* Mean follow-up is 5 years 3 months. Thirty-four patients are in complete remission, nine in stable disease, six evolutive disease, and one died with thoracic complications. Following repeated surgery, functional sequellaes are numerous: eight nerve palsies, eight articular stiffnesses. Major functional sequellaes came from surgery with radiotherapy. In one case, radiotherapy of a cervical lesion led to impossibility of surgery and to death.

The analysis of our series leads to schematising the indications of treatment.

1. If the desmoid tumor does not threaten life or limb, only surgery is needed as large as possible, avoiding functional sequellaes.
2. When tumoral relapses appear, treatment with Tamoxifen must be discussed.
3. When the tumor invades noble structures that could be hurt by surgery and lead to dramatic sequellaes, pre-operative chemotherapy is useful, in order to soften the tumor and to help surgery.

*Conclusion:* In this unforeseeable illness, backgrounds of indications are respective risks of spontaneous evolution and of treatment. Besides surgery, needed fast all cases but often insufficient, the value of interferon, tamoxifen and/or chemotherapy must be considered.

The most important concept is the necessity to adapt a well-balanced treatment avoiding sequellaes and particularly radiotherapy. This last attitude leads to most heavy sequellaes and therapeutic deadlocks.

### 081 Treatment Of Pelvic Ewing's Sarcoma With Multidisciplinary Treatment

Delepine N[1], Delepine G[1], Delepine F[2], Alkallaf S[1], Cornille H[1], Sokolov T[3]
[1]*Oncologic Paediatric Unit, Avicenne University Hospital, 125 Bd de Stalingrad, 93009 Bobigny Cedex, France, and* [2] *Bone Tumor Center, Sofia, Bulgarie, and* [3]*CHU de Rouen, Orthopaedic Department, France*

*Introduction:* Despite the improved survival of patients with Ewing's sarcoma, pelvic location remains a bad prognostic factor. This retrospective analysis tries to point out the reasons of such a situation, and to evaluate the impact of modem comprehensive approach on prognosis.

*Material and methods:* From February 1977 to June 1998, 53 patients have been treated by our group for Ewing's sarcoma of pelvic bones. Thirty-two were males, 21 females, aged 6–35 years (median, 16.3 years). At first screening, 15 patients had already metastases and 38 presented with localised disease.

Treatment included chemotherapy for all patients according to the current protocol at the time of presentation: four drugs (Vincristine–Dactinomycin–Cyclophosphamide–Doxorubicin), five drugs (Vincristine–Doxorubicin–Cyclophosphamide–Dactinomycine + ifosfamide) or six drugs association (Ifosfamide–Vincristine–Doxorubicin–Cyclophosphamide–Dactinomycine + Etoposide or Cisplatinium). Local treatment used radiotherapy alone for 24 patients, surgery alone in 18 and a combination in 11.

All patients have been followed up every 3 months for 2 years, every 6 months for 2 other years, and then yearly.

*Results:* With a median follow-up of 10 years, the 5-year actuarial event free survival rate for all patients is 31%; 13% for primary metastatic patients and 40% for patients seen with localised disease ($p < 0.001$). In primary localised tumor, the major prognostic factors are the adequacy of surgical resection ($p < 0.01$) and the high dose intensity of chemotherapy, particularly during the induction ($p < 0.05$).

Patients treated by radiotherapy had a 44% risk of local recurrence, 17% life expectancy, and a 13 month median survival compared with an 80% life expectancy and 80 month median survival for patients after wide resection.

*Conclusion:* (1) Primary metastatic patients require a new approach. (2) Early wide resection of the primary and adequate dose intensity of a six-drug chemotherapy gives the best results in pelvic Ewing's despite large tumoral volume or even incomplete response to pre-operative chemotherapy.

### Posters — Diagnostic Imaging/Pathology

### 024 Biological Characterisation Of Soft Tissue Sarcomas

Nielsen MM[1], Hansen O[1], Starklint H[2], Knoop A[1], Rose C[3]
[1]*Department of Oncology, Odense University Hospital, DK-5000 Odense C, Denmark,* [2]*Department of Pathology, Vejle Hospital, and* [3]*Department of Oncology, Lund University Hospital, Sweden*

*Introduction:* In an effort to identify better prognostic factors concerning patients with soft tissue sarcomas (STS), the importance of specific biological characteristics (changes in tumour suppressor genes, oncogene expression, and growth rate) has been addressed in a number of prognostic studies. Unfortunately, no consensus can be drawn from these studies, mainly due to limited number of studies and lack of proper methodology. Therefore, we decided to study the two suppressor gene products, p53 and the pRb1, the oncogene product mdm2 — all three involved in the cell cycle control — and the proliferation marker MIB1.

*Aim:* To analyse the prognostic significance of the clinical factors and the markers p53, pRb1, mdm2 and MIB1 in patients with STS.

*Material:* All patients with primary STS of the extremities, trunc, retroperitoneum, and head and neck were recruited from a predefined geographical area during the period 1 January 1970–31 December 1994. A total of 383 patients were included, of these 28 patients had metastatic disease, leaving 355 patients available for the survival analysis with a median follow-up of 13 years and 9 months; 154 patients had either a relapse or a progression of the tumour. Two hundred and sixteen patients died.

*Methods:* The clinical data was retrospectively collected by review of medical files. The expression of p53, pRb1, mdm2, and MIB1 in the tumours were detected using immuno-histochemistry. The survival analyses were performed using both univariate and multivariate methods with overall survival as end-point.

*Results:* We were able to create a prognostic model based on age at diagnosis, large size, high grade, deep sited tumours, retroperitoneal location that could identify four significantly different risk groups of patients with localised STS, with four different survival probabilities. The immunohistochemical markers MIB1 and pRb1

had independent prognostic value when adjusted for the effect of the clinical prognosticator, while p53 and mdm2 did not. High expression of MIB1 and pRb1 was correlated to poor prognosis. However, none of the indexes including the immunohistochemical markers predicted patient outcome better than the index including only clinical data.

*Conclusion:* The already known clinical prognosticators are still considered to be the best variables in predicting overall survival of patients with STS.

### 017 Good Pathological Response To Neoadjuvant Chemotherapy In Patients Showing Clinical Or Radiological Progression Of Disease – 3 Clincal Cases

Plummer R, Lucraft H, Murray S, Malcolm A, Dildey P, Grainger A, Baudouin C, Verrill M

*North of England Bone and Soft tissue Tumour Service, Freeman Hospital, Newcastle Upon Tyne, NE7 7DN, UK*

Neoadjuvant chemotherapy has an established role in the treatment of head and neck carcinoma, breast cancer and osteosarcoma. As well as an increase in the limb/breast conservation rate, pathological response to neoadjuvant chemotherapy is an independent prognostic marker in osteosarcoma[1] and breast carcinoma.[2] We report three cases of soft tissue sarcoma treated with neoadjuvant chemotherapy.

*Case 1:* A 50-year-old man presented with malignant fibrous histiocytoma of the right thigh. After two cycles of neoadjuvant doxorubicin (75 mg/m$^2$), there was clinical and radiological disease progression, and he proceeded to immediate surgery. There was 90% tumour cell necrosis in the operative tumour specimen. Further doxorubicin was given as adjuvant therapy.

*Case 2:* A 47-year-old woman presented with a high grade liposarcoma of the right thigh. She received one cycle of neoadjuvant doxorubicin (75 mg/m$^2$). There was clinical progression of the primary tumour, and she went forward to surgery immediately. A high degree of tumour necrosis was seen in the pathological specimen.

*Case 3:* A 44-year-old man presented with a right thigh high grade malignant fibrous histiocytoma. He received three cycles of ifosfamide (5 g/m$^2$)/adriamycin (50 mg/m$^2$). MRI scan showed enlargement of the tumour, with central necrosis. The surgical pathological specimen showed evidence of chemotherapeutic damage with scattered tumour cells in a background of reactive collagen and extensive haemorrhage.

Each of these patients received pre-operative chemotherapy with the aim of enabling limb conservation. In each case, there was clinical and/or radiological disease progression within three cycles of chemotherapy, and patients had early surgery. Histologic examination of each operative specimen revealed extensive tumour cell necrosis.

These favourable pathological responses were at odds with the clinical and radiological evidence of progressive disease by standard response criteria. Two of these patients were in clinical trials of chemotherapy and were recorded to have progressive disease when there was clearly an antitumour effect. In cases of early progression of a primary soft tissue sarcoma on chemotherapy, we recommend that investigators should consider either biopsy or dynamic gadolinium enhanced MRI scanning to detect pathological responses, which are unexpected on clinical and conventional radiological assessment

#### References

1. Davis AM, Bell RS, Goodwin PJ. Prognostic factors in osteosarcoma: a critical review. *J Clin Oncol* 1994; 12(2):423–31.
2. Fisher B, Bryant J, Wolmark N, Namounas E, Brown A, Fisher ER, Wickerham DL. Effect of pre-operative chemotherapy on the outcome of women with operable breast cancer. *J Clin Oncol* 1998; 16:2672–85.

### 018 Malignant Fibrous Histiocytoma Arising Within A Solitary Osteochondroma

Carluke I, A Grainger, Murray SA, Malcolm AJ

*The North of England Bone and Soft Tissue Tumour Service, The Freeman Hospital, Newcastle upon Tyne NE7 7DN, UK*

Malignant change of exostoses in patients who have multiple osteochondromas is well recognised. Chondrosarcoma is most commonly seen, although osteosarcoma and other soft tissue sarcomas have been described. Malignant fibrous histiocytoma arising within an exostosis in a patient who had multiple osteochondromas has been reported in the literature. We report an almost unique case of MFH occuring within a solitary osteochondroma arising from the proximal tibia of a 21-year-old male.

This was treated by *en bloc* excision, with allografting of the resultant defect. Pathological examination and immunohistochemical staining showed a focus of hypercellular spindle cell tumour embedded in a collagenous matrix, with marked positivity to SMA, within co-existent osteochondroma and bursa.

### 019 Sacrococcygeal Soft Tissue Ependymoma

Carluke I, Murray SA, Malcolm AJ

*The North of England Bone and Soft Tissue Tumour Service, The Freeman Hospital, Newcastle upon Tyne NE7 7DN, UK*

Ependymomas are glial derived tumours arising mostly intracranially or within the spinal cord, cauda equina or filum terminale.

Extra spinal ependymomas do occur, although rarely, and have been described arising within the pelvis and mediastinum, as well as the sacrococcygeal region. Sacrococcygeal ependymomas represent about 5% of all primary ependymomas. Metastatic behaviour is not predictable either clinically or histologically, although both recurrence and metastasis occur more frequently than in the intradural variety. Recommended treatment is wide excision.

We describe four cases of sacrococcygeal ependymoma treated with wide excision. Histological appearances were typical with immunocytochemical staining for glial fibrillary acidic protein (GFAP) strongly positive.

### 020 Tissue Microarray Blocks: An Efficient Method For Screening Soft Tissue And Bony Sarcomas

Thomas D, Baker L, Giordano T

*University of Michigan Comprehensive Cancer Center, Ann Arbor, MI 48109, USA*

As the human genome project reaches conclusion, the number of oncology-related genes and corresponding antibodies and nucleic add probes will continue to proliferate. Evaluation of these new markers using conventional tissue-based assays will require significant resources. The recently developed tissue microarray technique allows for the efficient screening of large numbers of tumor specimens with multiple probes. To date, no one has applied this technique to the screening of soft tissue and bony sarcomas. Basically, the technique involves the cutting of several core tissue biopsies (diameter, 0.6 mm; height, 3–4 mm) taken from representative 'donor' paraffin embedded tumor blocks and precisely arrayed into a 'recipient' paraffin block. Several tissue microarray blocks containing multiple cores from soft tissue and bony sarcomas were produced containing over 100 sarcoma specimens.

Sections cut from these arrays allow parallel detection of DNA or RNA by nonradioactive *in situ* hybridization and protein by immunohistochemistry. At least 200 consecutive 5 μm thick sections can be cut from each tumor block. This allows consecutive analysis of a large number of molecular markers.

In order to validate this approach of using small representative cores, the original donor blocks were also examined retrospectively for the expression of oncology-related markers such as telomerase reverse transcriptase (by *in situ* hybridization and immunohistochemistry), p53, Mb-l, ErbB2 (by immunohistochemistry) and apoptosis (by TUNEL assay), and were shown to correlate well with the results obtained with the tissue microarray blocks.
This technique represents an effective means of screening large numbers of specimens with multiple probes and antibodies.
*Acknowledgements:* Kindly supported by the Walther Cancer Institute.

### 021 Myofibroblast Modulation In Desmoid Tumors

Baldini N, Barbanti-Brodano G, Zini N, Bertoni F, Giunti A
*Istituti Ortopedici Rizzoli, 40136 Bologna, Italy*

Myofibroblasts play a role in several conditions, including fibromatoses. During granulation tissue contraction, a high proportion of myofibroblasts develop the expression of a-smooth muscle actin. When contraction stops, myofibroblasts containing a-smooth muscle actin disappear, and the scar becomes less cellular and composed of typical fibroblasts. Similarly, during the development of fibrocontractive diseases, fibroblasts acquire contractile features and produce the centripetal force leading to retraction. For this purpose, myofibroblasts develop connections to the surrounding extracellular matrix. The mechanisms leading to the modulation of myofibroblasts remain to be explored. We evaluated a retrospective series of 24 cases of desmoid tumors in order to analyze the expression of a-smooth muscle actin and of CD68 antigen, which identifies reactive mononuclear cells, on paraffin-embedded tissue sections by immunohistochemistry. A positive staining for a-smooth muscle actin, identifying myofibroblasts among the spindle cell component of the lesion, was found in 11/24 cases (46%), whereas the occurrence of CD68-positive mononuclear cells was detected in 19/24 cases (79%). Ultrastructurally, CD68-positive cells were found to correspond to two cell types, i.e. macrophages and mast cells. It may be hypothesized that these elements modulate myofibroblasts through a paracrine mechanism. It is well known that mast cell proliferation is accompanied by fibrotic changes, possibly through the release of heparin, a molecule that is capable to increase a-smooth muscle actin expression. Moreover, macrophages release growth factors, such as GM-CSF, that are implicated in the modulation of myofibroblasts. It is conceivable that therapeutic agents that interfere with the functional status of spindle cells may be used as a tool for the inhibition of growth of desmoid tumors, as previously demonstrated in other fibrocontractive diseases.

### 023 Her2/neu Expression In Osteosarcomas And Other Bony Sarcomas

Thomas D, Giordano T, Sanders D, Arrowsmith P, Baker L
*University of Michigan Comprehensive Cancer Center, Ann Arbor, MI 48109, USA*

Osteosarcoma and skeletal Ewing's sarcoma are the most common primary bone tumors in the pediatric age group. Both are aggressive malignancies with a tendency for early and rapid pulmonary metastasis. Advances in surgical oncology and adjuvant pre-operative chemotherapy have increased the overall 5-year survival to approximately 70%. However, although this represents the majority of patients, those who relapse rarely respond to salvage therapy. Clearly, there is a need for alternate adjuvant chemotherapy for these patients. Recently, there have been conflicting reports about the expression of c-erb B2 proto-oncogene product in osteosarcomas and Ewing's sarcoma. The c-erb B2 proto-oncogene encodes the human epidermal growth factor receptor 2 (Her2/Neu); a membrane bound tyrosine kinase, which when over expressed in rodent fibroblasts causes malignant transformation. Several groups of authors have claimed that a high percentage of osteosarcomas express Her2/Neu and that either this portends a poor prognosis or was associated with a decreased risk of relapse. Archival cases of osteosarcomas ($n$ = 38, including pre- and post-treatment samples; age range, 6–27 years) and Ewing's sarcoma ($n$ = 11; age range, 16 months–22 years) were retrieved and the diagnosis confirmed. Specimens were assessed for Her2/Neu oncogene expression by standard immunohistochemical techniques. Several cases demonstrated cytoplasmic staining, but none showed the membranous staining characteristic of over-expression by breast carcinomas. To validate the negative immunostains, reverse transcriptase polymerase chain reaction of RNA extracted from archival material also failed to demonstrate the presence of mRNA for c-ErbB2, even though appropriate internal controls were positive. Our results demonstrate that ErbB2 expression in either osteosarcomas and Ewing's sarcoma is not common and thus not likely to be an important prognostic factor, and further that therapy with recombinant humanized anti-HER2 monoclonal antibodies may not be appropriate therapy for these patients.
*Acknowledgements:* Kindly supported by the Walther Cancer Institute.

### 074 Matrix Gene Expression And Cellular Phenotyping In Chondroid Chordoma Reveals Focal Maturation Of Neoplastic Chordoid Cells Mimicking Histogenesis Of Developing Nucleus Pulposus

Gottschalk D, Fehn M, Patt S, Saeger W, Kirchner T, Aigner T
*Institute of Pathology, FAU Erlangen-Nürnberg, Germany*

*Introduction:* Beside conventional chordoma (cchor), so-called chondroid chordoma (chon chor) is described as a specific subtype. However, since its first description, conflicting results have been reported on the existence of this cartilaginous tumor variant, and several studies suggested chon chor being in fact low-grade chondrosarcomas. In the present study, we used molecularbiological *in situ* localization techniques for mRNAs of different collagen types and the proteins as marker gene products, indicating different phenotypic or developmental stages of chondrocytes, to elucidate further the origin and histogenesis of the chondroid tumor compartment in chon chor.
*Materials and methods:* Seven specimen of chon chor and 14 of cchor were routinely fixed immediately after removal and embedded in paraffin. Additionally, four samples of fetal vertebral columns with remnants of chorda dorsalis (cd) tissue were included. Histomorphological evaluation was performed with HE, GAGs were visualized by toluidine blue, and the presence of collagens by Masson–Goldner's staining.
*Immunohistochemistry:* Deparaffinized sections were enzymatically pretreated and stained with primary antibodies against collagen type I, II, III, VI and X, aggrecan proteoglycan, S-100, vimentin, EMA, CK-19 and Pan-CK. *In situ* hybridization was performed as described by our group elsewhere.
*Results:* We could clearly demonstrate the multifocal deposition of the cartilage-specific type II collagen protein and mRNA in cchor and chon chor, and thus clearly demonstrate the chondrogenic potential of all chor irrespective the appearance of ouvert cartilage formation. Aggrecan expression was present throughout all chor and, thus, a very characteristic gene product of these neoplasms also in the absence of chondroid differentiation. There was a clear biochemical resemblance of cchor and the chordoid tumor compartment of chon chor to the cd. Respectively, the chondroid tumor compartment of chon chor resembled the adult, matured nucleus pulposus (np). Noteworthy, no type X collagen could be demonstrated in any of our chor, explaining the lack of chondroid calcification in these neoplasms. Further studies will show whether type X-collagen could be used as a marker gene product to differentiate between cartilaginous entities.

*Discussion:* In the present study, we investigated the biochemical composition of the extracellular tumor matrix as well as the matrix gene expression pattern in conventional and chondroid chordomas in comparison with cell and tissue morphology as well as the cytoprotein profile of the neoplastic cells. Herein, the analysis of the matrix gene expression pattern allows one to identify and characterize cartilaginous cell differentiation within the neoplasms, which is not unequivocally possible by histomorphological, ultrastructural or cytoprotein analysis. Using this approach, we could identify and trace the cellular differentiation pattern in chordomas including the chondroid variants and could identify chondroid differentiation *sui generis* as a characteristic facette of a subset of chordomas, here mimicking the histogenesis of the developing np.

**093 Host Immune Response In Osteosarcoma**

Casanova J, Reith JD, Berrey BH, Enneking WF, Scarborough MT
*University of Florida, Gainesville, FL 32610, USA*

*Background:* Few previous studies document the role and significance of the host immune response in high-grade osteosarcoma. This retrospective analysis examines the nature of the inflammatory response found in and around osteosarcomas and the relationship to disease free and overall survival.

*Patients and methods:* All patients had stage IIB osteosarcomas about the knee (distal femur, proximal tibia). Group A included 20 patients who were biopsied and treated with neoadjuvant chemotherapy and surgical resection. Group B included 73 patients who underwent surgical resection of their tumor and were treated with post-operative chemotherapy. All histologic slides examined included tumor and the adjacent reactive zone. Each case was evaluated with antibodies to CD3, CD4, CD8, CD20, and TIA-1 using standard avidin–biotin complex methods. The number of CD3-, CD8-, and CD20-positive inflammatory cells was further quantified by histomorphometry.

Results:

| Antibody | Total | Group A | Group B | *p* value |
|---|---|---|---|---|
| CD3 | 82/93 (88%) | 20/20 (100%) | 62/73 (85%) | 0.04 (group B) |
| | | Mean, 123 cells | Mean, 88 cells | |
| CD4 | 74/93 (80%) | 17/20 (85%) | 57/73 (78%) | < 0.01 |
| CD8 | 75/93 (81%) | 18/20 (90%) | 57/73 (78%) | < 0.002 |
| CD20 | 49/93 (53%) | 10/20 (50%) | 39/73 (53%) | Not significant |
| TIA-1 | 30/93 (32%) | 9/20 (45%) | 21/73 (29%) | Not significant |

*Conclusions:* An increased number of inflammatory reaction around osteosarcomas appears to be related to better survival, particularly when patients are given neoadjuvant chemotherapy. Presence of high numbers of T-cell tumor infiltrating lymphocytes (CD-3, CD4-, CD8-positive cells) correlates with improved survival. B cells and natural killer cells (CD20- and TIA-1-positive, respectively) appear to play a less significant role in the immune response.

**094 Molecular Predictors Of Outcome In Patients With Osteosarcoma**

Reith JD, Casanova J, Berrey BH, Enneking WF, Scarborough MT
*University of Florida, Gainesville, FL 32610, USA*

*Background:* A variety of molecular markers related to survival, including bcl-2, p53, p-glycoprotein, CD95 (Fas), CD95-ligand (Fas-L), CD44, and CD44v, have been studied in a variety of human neoplasms, particularly carcinomas. Their significance in patients with osteosarcoma is largely unknown. The purpose of this archival study was to determine if there is a correlation between expression of these factors and disease free and overall survival for patients with osteosarcoma.

*Patients and methods:* All patients had stage IIB osteosarcomas originating around the knee (distal femur, proximal tibia). Group A included 20 patients who underwent biopsy and were treated with neoadjuvant chemotherapy and surgical resection. Group B included 73 patients who underwent primary surgical resection of their tumor followed by post-operative chemotherapy. Tumors were evaluated with antibodies to bcl-2, p53, Fas, Fas-L, CD44, CD44v6, and *p*-glycoprotein using standard avidin–biotin complex methods. Expression of the various antigens was compared with disease-free and overall survival, accounting for percent necrosis (Group A) and margin status (Groups A & B).

Results:

| Antibody | Total positive | Group A | Group B | *p* value |
|---|---|---|---|---|
| bcl-2 | 0/93 | 0/20 | 0/73 | Not significant |
| p53 | 17/93 (18%) | 6/20 (30%) | 13/73 (15%) | Not significant |
| CD95 | 9/93 (10%) | 1/20 (5%) | 8/73 (11%) | < 0.05 |
| CD95-L | 52/93 (56%) | 16/20 (80%) | 36/73 (49%) | Not significant |
| CD44 | 28/93 (30%) | 11/20 (55%) | 17/73 (23%) | Not significant |
| CD44v6 | 13/93 (14%) | 6/20 (30%) | 7/73 (10%) | < 0.03 |
| *p*-Glycoprotein | 46/93 (49%) | 8/20 (40%) | 38/73 (52%) | 0.10 |

*Conclusions:* CD95 appears to have a ‘protective’ function in patients with osteosarcoma, probably by allowing tumor cells to proceed through apoptosis pathways to cell death. Although CD44v6, a vascular adhesion molecule, was identified in only 14% of the total cases, its expression correlated with subsequent development of metastases and death (11 of 13 patients developed pulmonary metastases, 10 dying of disease). Although *p*-glycoprotein did not reach statistical significance, there was a trend toward death from disease in patients expressing it.

**103 A Case Of Gastro Intestinal Sarcoma Tumor (Gist) Revealed By A Mediterranean Kaposi’s Sarcoma In An Hiv-negative Patient: Casual Association Or Not?**

Vincent Baty[1], Isabelle Ray-Coquard[1], Eric Fontaumard[1], Dominique Ranchere-Vince[2], David Tavan[1], Jean-Yves Blay[2]
[1]*Clinique Eugene Andre, and* [2]*Centre Leon Berard, lyon, France*

Mediterranean Kaposi’s sarcoma (KS) typically runs a chronic course in elderly patients and may be associated with secondary malignancies. HHV-8 has been well documented to be associated with all forms of KS. Gastrointestinal stromal tumors (GIST) are a recently described entity whose etiology remains unknown. The possible role of human herpes-virus 8 (HHV-8) in this disease has not been reported. We hereafter report the first case of an association of a colonic GIST with KS in an HIV-negative patient. A 72-year-old Spanish woman was admitted with a few months history of flat skin lesions and nodules on her left knee. The histopathological study of skin biopsies demonstrated Cutaneous Kaposi’s sarcoma (CKS). Endoscopic explorations of the gastrointestinal tract were performed as a standard staging procedure and revealed a stenotic tumor on the transverse colon. The histopathological and immunochemistry studies of colonic tumor enabled identification of a GIST. A segmental surgical resection was subsequently performed. The tumor size was 4 x 1.5 x 1.5 $cm^3$. The association of Kaposi’s sarcoma and smooth muscle tumors of the gastrointestinal tract is extremely rare since only one case has been reported in an HIV-positive patient. Infection with

HHV-8 is identified in more than 95% of KS and has been demonstrated in other skin cancer in immunosuppressed patients, and for angiosarcoma in a HIV-negative patient. Conversely, latent EBV infection has been demonstrated in low-grade leiomyosarcoma (LS) in HIV-positive patients but neither in adjacent Kaposi s sarcoma lesions nor in LS occuring in immunocompetent hosts. Thus, our case is remarkable since the association of LS and CKS has not been reported previously in a non-HIV patient, and suggests a possible association between HHV-8 infection and GIST. The presence of Epstein–Barr virus and HHV-8 DNA sequences is investigated on both CKS and GIST tissue specimens, and the results will be available for presentation at CTOS.

**Posters — Oncology Research**

**009 Hypoxia In Human Soft Tissue Sarcomas: Adverse Impact On Survival And No Association With P53 Mutations**

Nordsmark M[1,2], Alsner J[1], Keller J[3], Nielsen OS[2], Jensen OM[4], OvergaardJ[1]

*[1]Department of Experimental Clinical Oncology, [2]Department of Oncology, [3]Department of Orthopaedic Surgery and [4]Department of Pathology, Danish Cancer Society, Aarhus University Hospital. Nörrebrogade 44, bldg 5, DK-8000 Aarhus C, Denmark*

Pre-clinical studies have suggested that cells with mutations in the tumour suppressor gene, p53 have a reduced apoptotic potential under hypoxic conditions and that this may lead to progression of neoplastic cells into a more malignant phenotype. The present study correlated tumour oxygenation and p53 status in human soft tissue sarcomas (STS) and compared oxygenation status with treatment outcome after 5 years follow-up. Pretreatment tumour oxygenation measurements were performed in 33 patients with STS using polarographic needle electrodes (Eppendorf, Germany), and status of the p53 gene was determined in 32 of those by PCR using DNA extracted from paraffin-embedded ($n$ = 2) sections or frozen biopsies ($n$ = 30). Overall median of the tumour median $pO_2$ was 15 mmHg (range, 1–58 mmHg). Only six tumours had p53 mutations and no association was found between mutant p53 and hypoxia. Twenty-eight cases could be evaluated by actuarial survival estimates. At a median follow-up of 74 months, 12 patients had died. When stratifying into well-oxygenated and hypoxic tumours by overall median $pO_2$, patients with hypoxic tumours had a poorer disease specific survival probability (log-rank, $p = 0.05$) and a poorer overall survival (log-rank, $p = 0.01$) at 5 years. In conclusion, we demonstrated that hypoxia was a marker for metastatic disease and poorer overall survival in human STS. Moreover, tumour oxygenation and p53 status were not associated.

*Acknowledgements:* Supported by grant form the Danish Cancer Society.

**101 The Functional And Local Results Of Limb Sparing Procedures In Upper Girdle Neoplasms Treatment**

Dardzinski R, Ruka W, Koziol A, Rutkowski P Maria

*Sklodowska–Curie Memorial Cancer center, Warshaw, Poland*

Thirty-one patients (15 females and 16 males) were operated on in Maria Sklodowska-Curie Memorial Cancer Centre from 1980 to 1999 (altogether 33 operations were performed). Mean age at operation was 37.0 years (range, 13–68 years). Fifteen tumors involved the scapula, while in 16 cases neoplasms were located in proximal humerus.

In three cases, soft tissue sarcoma was diagnosed, and all other cases were primary bone tumours. All patients with osteosarcoma received both pre- and post-operative chemotherapy. In cases of soft tissue sarcomas, neoadjuvant chemotherapy was also introduced. The follow-up periods of patients ranged from 6 to 236 months (mean, 42 months).

All surgical procedures were classified according to the system elaborated by Malawer. There were six operations of the V1 type and 11 resections of the V type. Less extensive procedures were performed in 16 patients — three, seven and six operations in types I, II, and III, respectively. Only in one case was internal prosthesis inserted; all other patients were operated without bone length reconstruction. External orthopaedic devices were used instead.

The functional results were assessed according to the MSTS evaluation system including the analysis of six factors. The patient's occupation and recreational abilities were particularly emphasized. Simplified, a three-factor evaluation scale was also introduced and compared with the MSTS system. Analysis of local recurrence as the main drawback of limb sparing surgery was performed. At the moment, 22 patients are alive free of disease. Out of nine patients who died, four deceased with symptoms of local recurrence. In two cases, local recurrence was accompanied or preceeded by systemic dissemination. One patient is free of disease 84 months after reoperation of his recurrence. Together, local recurrence was observed in five out 31 patients (18.5). No evident correlation between local recurrence and the extent of the free margin was found. All failures concerned advanced cases — II B according to the TNM staging system.

Non-oncological complications were infrequent (6/31). None of them resulted in permanent disability. Only one temporary palsy of the radical nerve was observed.

The mean functional score in accordance with MSTS rating system was 72.6% (range, 50–90%). This outcome corresponds to the data published by the others. The lack of prosthetic reconstruction resulted in inferior hand positioning, but due to the application of external devices, does not seem to effect significantly patients' ability.

**102 Lymphopenia (Lyp) As An Independent Prognostic Factor For Survival In Advanced Soft Tissue Sarcomas As Well As In Lymphomas, And Metastatic Breast And Renal Carcinoma**

Blgy J, Ray-Coquard I, van Glabbeke M, Nøgrier S, Saghatchian M, Verweij J, Sebban C, Escudler B, Guastalla J-P, Judson I, Nielsen OS, Biron P, Le Cesne A, Centre L. Børard, H.Ed. Herriot, Lyon, I.G. Roussy, Villejulf, France, and EORTC STSSG

*Bruxelles, Belgium*

Lyp is frequently observed in patients (pts) with advanced cancers and is a powerful predictor of the toxicity of chemotherapy (*J Clin Oncol* 14:636; *J Clin Oncol* 17:2W; *Blood* 92:405), Here, we analysed the prognostic value of lyp on survival after chemotherapy or immunotherapy in four distinct tumor types. The prognostic value of lyp was tested in databases of previously reported multicentric prospective studies of systemic therapies in untreated pts: (1) of CYVADIC in advanced soft tissue sarcomas (ASTS) ($n$ = 191) (*Cancer* 1984; 53:1825); (2) of CEF chemotherapy in metastatic breast carcinoma (MBC) ($n$ = 280) (*Bull Cancer* 1990; 77:941); (3) of IL-2 ± IFNα in metastatic renal cell carcinoma (MPCC) ($n$ = 481) (*N Engl J Med* 1998; 338:1272); and in a prospective monocentric serie of agressive nonHodgkin's lymphomas (NHL) treated in consecutive phase III studies in the CLB between 1987 and 1993 ($n$ = 322). The incidence of lyp < 1000/μl was remarkably constant among these four series, i.e. 240/a, 24%, 20% and 27%, respectively. Lymphopenia was significantly more frequent ($p < 0.05$) in pts with performance status (PS) > l in ASTS, MBC, and MRCC series, and in pts aged > 60 ($p < 0.05$) with NHL. Lyp < 1000/μl was not significantly correlated to response to chemotherapy of inummotherapy in multivariate analysis using logistic regression in any series tested. In contrast, lyp < 1000/μl significantly correlated to overall survival in univariate analysis (median survival (MS)): in ASTS, 6 months versus 12 months ($p < 0.001$); in MBC, MS 10 months versus 14 months ($p < 0.01$); in NHL, MS 11 months versus 94 months ($p < 0.001$)' and in MPCC (lyp < 1500/μl), MS 11 months versus 18 months ($p = 0.01$). In multivariate analysis using Cox model, lyp was found to be an independent prognostic factor for overall survival in ASTS (RR, 1.46; 95% CI, 1.001–2.142) along with liver metastases (mets) and PS; in MBC (RR, 1.75; 95% CI, 1.3–2.4) along with mets and PS; in MPCC (RR, 1.2; 95% CI, 0.97–1.51) along with met free interval, number of met sites, loss > 10% of body weight and PS > O; and in NHL (RR, 1.47; 95% CI, 1.02–2-1, along with prognostic factors of the International Prognostic Index). We conclude that lymphopenia is a general and yet unrecognised prognostic factor for overall survival in wide variety of frequent cancers including advanced soft tissue sarcomas.
